# Whole genomic sequence analysis of *Bacillus infantis*: defining the genetic blueprint of strain NRRL B-14911, an emerging cardiopathogenic microbe

**DOI:** 10.1186/s12864-016-2900-2

**Published:** 2016-08-22

**Authors:** Chandirasegaran Massilamany, Akram Mohammed, John Dustin Loy, Tanya Purvis, Bharathi Krishnan, Rakesh H. Basavalingappa, Christy M. Kelley, Chittibabu Guda, Raúl G. Barletta, Etsuko N. Moriyama, Timothy P. L. Smith, Jay Reddy

**Affiliations:** 1 School of Veterinary Medicine and Biomedical Sciences, University of Nebraska-Lincoln, Lincoln, NE 68583 USA; 2 University of Nebraska Medical Center, Omaha, NE 68198 USA; 3 Kansas State Veterinary Diagnostic Laboratory, Manhattan, KS 66506 USA; 4 Genetics, Breeding and Animal Health Unit, U.S. Meat Animal Research Center, Clay Center, NE 68933 USA; 5 School of Biological Sciences and Center for Plant Science Innovation, University of Nebraska-Lincoln, Lincoln, NE 68588 USA

**Keywords:** *Bacillus* sp. NRRL B-14911, Genome, Heart

## Abstract

**Background:**

We recently reported the identification of *Bacillus* sp. NRRL B-14911 that induces heart autoimmunity by generating cardiac-reactive T cells through molecular mimicry. This marine bacterium was originally isolated from the Gulf of Mexico, but no associations with human diseases were reported. Therefore, to characterize its biological and medical significance, we sought to determine and analyze the complete genome sequence of *Bacillus* sp. NRRL B-14911.

**Results:**

Based on the phylogenetic analysis of 16S ribosomal RNA (rRNA) genes, sequence analysis of the 16S-23S rDNA intergenic transcribed spacers, phenotypic microarray, and matrix-assisted laser desorption ionization time-of-flight mass spectrometry, we propose that this organism belongs to the species *Bacillus infantis*, previously shown to be associated with sepsis in a newborn child. Analysis of the complete genome of *Bacillus* sp. NRRL B-14911 revealed several virulence factors including adhesins, invasins, colonization factors, siderophores and transporters. Likewise, the bacterial genome encodes a wide range of methyl transferases, transporters, enzymatic and biochemical pathways, and insertion sequence elements that are distinct from other closely related bacilli.

**Conclusions:**

The complete genome sequence of *Bacillus* sp. NRRL B-14911 provided in this study may facilitate genetic manipulations to assess gene functions associated with bacterial survival and virulence. Additionally, this bacterium may serve as a useful tool to establish a disease model that permits systematic analysis of autoimmune events in various susceptible rodent strains.

**Electronic supplementary material:**

The online version of this article (doi:10.1186/s12864-016-2900-2) contains supplementary material, which is available to authorized users.

## Background

Heart failure (HF), a condition in which the heart is unable to adequately pump blood to rest of the body, is a leading cause of death worldwide. Estimates indicate that the current prevalence rate of HF is 2.8 %, and 825,000 new cases are reported annually in the United States alone [1]. While HF tends to be more prevalent in men than women in the age group of 40 to 79 years (1.73 to 2.1 %), women 80 years or older are more prone to the disease than men in that age group (1.4 %) [1]. Furthermore, the prevalence of HF is projected to increase from 2.8 % in 2010 to 3.3 % in 2025, and the economic loss resulting from HF is expected to double (~$34.1 billion in 2010 to ~$70 billion in 2025), in spite of continued efforts to contain the disease’s occurrence in the general population [2].

Various cardiovascular disease conditions have been implicated in the development of HF. These include pericardial and valvular diseases, atherosclerosis, hypertension, chronic ischemia, arrhythmia, diabetes, and myocarditis. Among the infectious causes, myocarditis has been identified as one important cause of HF in children and young adults. While most individuals affected with myocarditis may remain asymptomatic, 10 % can develop clinical heart disease. A proportion of these chronically affected individuals may develop dilated cardiomyopathy (DCM), and approximately half of them may undergo heart transplantation due to the lack of effective treatment options.

Furthermore, it is estimated that approximately 2 million Americans appear to have inflammatory heart infiltrates, raising the possibility that a subset of people may have an ongoing silent myocarditis [3]. Two observations support this notion: (1) apparently healthy individuals like athletes can die from sudden death syndrome, and their autopsies suggest the presence of inflammatory infiltrates [4], and (2) a necropsy study involving more than 12,000 victims of accidental deaths not related to any cardiovascular diseases showed evidence of myocarditis in 1.05 % of cases [5]. Thus, identifying the triggers of myocarditis may provide opportunities to treat affected individuals in a timely fashion.

The molecular mimicry hypothesis has been proposed as one major mechanism for the occurrence of autoimmune diseases including myocarditis, whereby the structural similarities between self and foreign antigens lead to recognition of self-antigens by generating cross-reactive immune responses [6, 7]. Numerous examples exist to support this theory in various disease conditions, such as experimental autoimmune encephalomyelitis/multiple sclerosis, experimental autoimmune uveoretinitis/uveitis and experimental autoimmune myocarditis/heart autoimmunity [7–13]. The importance of the molecular mimicry hypothesis can be summarized with two major predictable outcomes. (1) As the genomes of more microbes are sequenced, the search for mimicry sequences in the microbial databases has become relatively easy. These searches may result in the identification of microbes that are either natural pathogens of humans or are otherwise innocuous environmental isolates, but with the potential for them to trigger autoimmune diseases in those exposed. (2) Exposure to microbes carrying the mimicry sequences may result in the generation of cross-reactive immune responses leading to the induction of organ-specific autoimmunity. In our investigations, we identified a mimicry sequence for cardiac myosin heavy chain (Myhc)-α 334-352 contained in *Bacillus* sp. NRRL B-14911; the epitope, termed BAC 25-40, induces myocarditis by active immunization in A/J mice [12].

Because the biological and medical significance of this bacterium was not known, particularly regarding pathogenicity, we sought to analyze the complete genome of the organism to determine its phenotypic and virulence characteristics. By adopting phylogenetic analysis of 16S ribosomal RNA (rRNA) genes, sequence analysis of the 16S-23S rDNA intergenic transcribed spacers (ITS), phenotypic microarray (PM), and matrix-assisted laser desorption ionization time-of-flight (MALDI-TOF) mass spectrometry (MS), we propose the species and strain of *Bacillus* sp. NRRL B-14911 to be *Bacillus infantis* NRRL B-14911. The availability of the complete genome sequence for this bacterium may facilitate genetic manipulations to assess gene functions associated with bacterial survival and virulence. Additionally, this bacterium can serve as a useful tool to establish a novel disease model for autoimmune myocardial damage of infectious origin.

## Methods

### Bacterial strain, culture conditions and isolation of genomic DNA

*Bacillus* sp. NRRL B-14911 was procured as a kind gift from the Agricultural Research Service (ARS) Culture Collection, United States Department of Agriculture (Washington, DC). *Bacillus infantis* sp. nov. (type strain SMC 4352-1 T = KCCM 90025 T = JCM 13438 T) was procured form Japan collection of microorganisms (Koyadai, Japan). For isolation of genomic DNA, bacteria were grown in Luria Bertani (LB) broth (volume to flask ratio of 1:10) at 37 °C with gentle shaking at 200 rpm for 36 h. The genomic DNA was isolated using Qiagen genomic-tip 100 as recommended by the manufacturer (Qiagen, Valencia, CA).

### Genome sequencing, analyses and annotation

The genomic DNA of *Bacillus* sp. NRRL B-14911 was prepared for sequencing on the Pacific Biosciences RSII instrument as detailed in the procedure provided by the manufacturer (Procedure and Checklist - Greater Than 10 kb Template Preparation and Sequencing, Dec. 2012 version). Briefly, DNA was sheared using a Covaris G-tube (Covaris Inc., Woburn MA) to achieve fragments in the 5000-15,000 base pair range. A library was prepared from the DNA with size selection performed only by precipitation of the DNA onto AMPure PB beads, using the DNA Template Prep Kit 2.0 (Pacific Biosciences, Menlo Park CA). Sequencing was performed using XL/C2 chemistry in two SMRT cells, producing 176,090 reads with average read length 5185 bases, and 240,232 subreads (912 Mb total sequence) with average subread length 2972 bases (N50 = 4481 bases). Assembly was performed using PacBioToCA for error correction and Celera Assembler v7 for assembly as described [14]. Two contigs, one representing the bacterial chromosome and one representing a plasmid, were produced. The ends of the chromosomal contig were examined for overlap using nucleotide Basic Local Alignment Search Tool (BLASTN), which identified the most likely position at which the chromosome could be circularized. After removing redundant sequence, the origin was estimated by GenSkew (http://genskew.csb.univie.ac.at) analysis, and the linear contig was reset so that the estimated origin was base 1. The assembly was improved by polishing with Quiver from the SMRTportal software package (Pacific Biosciences), which fixed the insertions common in initial assemblies, and also confirmed the correct positioning during the circularization step. The finished assembly was annotated by NCBI, and annotation anomalies identified were curated and revised in Geneious (Biomatters ltd., New Zealand).

### Phylogenetic analysis

Phylogenetic analysis was done using the 16S ribosomal DNA sequences from 28 selected species (23 from the genus *Bacillus* and 5 from its related genera *Halobacillus*, *Oceanobacillus*, *Geobacillus* and *Paenibacillus*). All sequences were obtained from the GenBank database at the National Center for Biotechnology Information (NCBI). The sequences were aligned using MAFFT (v7.130b) with the L-INS-i algorithm [15]. The maximum likelihood phylogeny was reconstructed using PhyML (version 3.0) [16] with the GTR substitution model, the proportion of invariable sites and gamma shape parameters both estimated, and the option to choose the best tree from the nearest-neighbor interchange tree-rearrangement and subtree-pruning/regrafting. Non-parametric bootstrap analysis was done with 1000 pseudoreplicates.

### Comparison of conserved ITS sequences between *Bacillus* sp. NRRL B-14911 and *B. infantis* based on genomic DNA PCR analysis

Because that 16S-23S rDNA ITS sequences are hypervariable, but conserved within the same species, their sequence analyses have been successfully used for speciation of the genus *Bacillus* [17–19]. Briefly, 16S-23S rDNA ITS regions were amplified from the genomic DNA extracted from *Bacillus* sp. NRRL B-14911 and *B. infantis* JCM 13438 T using the primers specific to *Bacillus* genus as described previously [17, 18]. The primer sequences used were: 5'-GTCGTAACAAGGTAGCCGTA-3'/5'-CAAGGCATCCACCGT-3'; 5'-CCTTGTACACACCGCCCGT-3'/5'-AAAATAGCTTTTTGGTGGAG-3' ; and 5'-AAATTTGTATGGGCCTATAG- 3'/5'-GTGGGTTTCCCCATTCGG-3', and the amplifications were performed using the following conditions: 94^o^ C for 4 min followed by 32 cycles, each consisting of 94^o^ C for 1 min, 54^o^ C for 1 min, 72^o^ C for 2 min with a final extension at 72^o^ C for 10 min. After resolving the PCR products in 1 % agarose gel, the PCR amplicons were excised, purified using gel extraction kit (Qiagen, San Jose, CA) and subjected for sequencing. After excluding the 16S and 23S rDNA sequences from the amplicons, the nucleotide sequences representing only the ITS regions were recovered, and their percent identities were analyzed using William Pearson’s lalign program (http://www.ch.embnet.org/software/LALIGN_form.html).

### Phenotypic and biochemical characterization

Phenotypic analysis was performed using the Biolog GEN III microplate using Omnilog Data collection software (Biolog, Inc., Hayward, CA) [20, 21]. The components in the wells of the 96-well plates were comprised of sources for carbon (C), nitrogen (N), phosphorous (P), sulfur (S) and amino acids. The tests included: utilization of sugars, amino acids and organic acids; tolerance to NaCl; and susceptibility to antibiotics. To perform a comparative analysis, *Bacillus* sp. NRRL B-14911 and *B. infantis* JCM 13438 T were subcultured twice in isolation medium (trypticase soy agar with 5 % sheep blood, Remel, Thermofisher Scientific, KS) and inoculated individually in the wells of microplate. Protocol A was used and analysis was performed at 10 h postincubation, as per the manufacturer’s recommendation for members of the genus *Bacillus*. In addition, testing for Gram-staining, oxidase and catalase activities, and endospore formation were performed by standard microbiological procedures. Carbohydrate fermentation testing results for sorbitol, inulin, and lactose were confirmed using rapid fermentation tablets (Wee-Tabs, Key Scientific, Stamford Texas). Antimicrobial susceptibility testing for vancomycin was performed using Kirby-Bauer disk diffusion susceptibility testing following clinical and laboratories standards institute (CLSI) guidelines. As no interpretive criteria for assessing disk diffusion breakpoints for *Bacillus* sp. have been determined, interpretive criteria from CLSI M100-S22 for *Staphylococcus* sp. was applied to determine *in vitro* susceptibility breakpoints.

### Spore staining

Bacterial smears prepared on glass slides were fixed by methanol and air-dried. The smears were then stained with malachite green solution (5 min) under steam, washed and counterstained with safranin (30 s). After washing, the slides were air-dried and examined under the microscope with an oil immersion lens.

### MALDI-TOF MS analysis

The *Bacillus* sp. NRRL B-14911 and *B. infantis* JCM 13438 T cells grown in LB broth at 37^o^ C were plated onto tryptic soy agar with 5 % sheep blood agar plates. Following overnight incubation, individual colonies were picked and spotted onto the MALDI-TOF target. The spots were overlaid with 1 μl of α-cyano-4-hydroxycinnamic acid (HCCA) matrix (Bruker), and the mass spectra were acquired using MALDI-TOF MS, Microflex LT system in a linear positive mode (Bruker Daltonik, Billerica, MA). Instrument calibration was performed using standard reference BTS *Escherichia coli* (Bruker)*.* For bacterial identification, MALDI Biotyper 3.0, Reference Library 1.0 Version 3.1.2 was used [22, 23]. The cut-off scores used for species identification were: 2.300 to 3.000–highly probable species identification; 2.000 to 2.299–secure genus identification and probable species identification; 1.700 to 1.999–probable genus identification; and 0.000 to 1.699–not reliable for species identification.

### Sequence analysis of Allantoate amidohydrolase gene

Allantoate amidohydrolase (AAH) gene was amplified from the genomic DNA obtained from *Bacillus* sp. NRRL B-14911 and *B. infantis* JCM 13438 T using sequence specific primers (5'-GCTGGCTTGAAAAAAATC-3'/5'-GGAGGCAAATTCATCTGG-3'). The PCR products were resolved by 1 % agarose gel electrophoresis, and the amplified product was purified using gel extraction kit and subjected for sequencing.

### Analysis of *Bacillus* sp. NRRL B-14911 genome for virulence factors

The Virulence Factor Database (VFDB; http://www.mgc.ac.cn/VFs/) is a database constructed by the virulence-guided classification system. The core dataset of VFDB (VFs.faa) consisting of 502 virulence factors (VFs) from 2505 VF-related genes representing 25 genera of pathogenic microbes [24] was downloaded and used as the database for protein sequence similarity search. Using this information, we performed three types of analyses: First, we identified potential virulence factors from the proteins encoded in the *Bacillus* sp. NRRL B-14911 genome using protein Basic Local Alignment Search Tool (BLASTP) (version 2.2.25+) [25]; second, we blasted the proteomes of three pathogenic bacilli [*B. anthracis* str. Ames, GenBank Accn# NC_003997.3; *B. cereus* ATCC 14579, GenBank Accn# NC_004722.1; *B. licheniformis* ATCC 14580, GenBank Accn# NC_006270.3]; and three non-pathogenic bacilli [*B. pseudofirmus* OF4, GenBank Accn# NC_013791.2; *B. amyloliquefaciens* subsp. plantarum UCMB5033, GenBank Accn# NC_022075.1; *B. subtilis* subsp. subtilis str. OH 131.1, GenBank Accn# NZ_CP007409.1] against the protein sequences in the VFDB database to identify the potential virulence factors present in the respective groups. These were then compared with those of *Bacillus* sp. NRRL B-14911 to identify the virulence factors unique to this bacterium; and third, we compared the virulence factors of the pathogenic bacilli as described above with those of *Bacillus* sp. NRRL B-14911 to identify those that are common to both. The thresholds used were E-value of 1×10^-10^ and bit score of 40. CGView server was used to draw the circular map to show the location of potential virulence factor genes in *Bacillus* sp. NRRL B-14911 [26].

### Comparative analysis of the *Bacillus* genomes

For comparative genomic analysis of *Bacillus* sp. NRRL B-14911 with other species within the genus *Bacillus*, we downloaded the complete genome annotations of *B. subtilis* strain 168 [GenBank Accn# 225184640], *B. megaterium* DSM 319 [GenBank Accn# CP001982.1], *B. thuringiensis* serovar kurstaki strain HD73 [GenBank Accn# CP003889.1] and *B. cereus* ATCC 14579 [GenBank Accn# AE016877.1] from the NCBI database. The presence or absence of genes encoding methyltransferases and transporters as well as insertion sequence (IS) elements was determined based on the “product” assignments in each genome annotation. The comparative analysis for enzymes and biochemical pathways was performed using pathway mapping for each genome in the Kyoto Encyclopedia of Genes and Genomes (KEGG) database [27].

## Results and discussion

We report here the complete genome sequence analysis of the bacterium *Bacillus* sp. NRRL B-14911 that has a potential to induce heart autoimmunity by molecular mimicry. *Bacillus* sp. NRRL B-14911 was originally isolated from ocean water at a depth of 10 m in a sea expedition seeking to study the marine microflora in the Gulf of Mexico and around the Bimini Islands. However, the possible significance of this bacterium as a pathogen was unknown [28]. Based on our discovery that *Bacillus* sp. NRRL B-14911 contains a disease-inducing mimicry epitope for cardiac myosin, we sought to determine the biological significance of this organism to humans. To this end, we decided to sequence the complete genome of *Bacillus* sp. NRRL B-14911 and characterize its phenotypic and biochemical features with the expectation that identification of its species may create opportunities to establish a new disease model to study the autoimmune events of bacterial myocarditis in experimental settings.

### Genome sequencing

Morphologically, *Bacillus* sp. NRRL B-14911 was found to be a Gram-positive, rod-shaped, sub-terminal endospore-forming, aerobic bacterium with rounded ends as observed by light microscopy (Additional file 1: Figure S1). For complete genome sequencing, we isolated the genomic DNA and performed sequencing in long-reads by PacBio RS SMRT sequencing technology [29, 30]. The sequencing produced >900 Mb of post-filter sequences, consisting 176 K reads of average >5 100 bases. Initial assembly of the genome produced a circularizable contig of 4,884,884 bases. The assembly was further refined in Quiver to generate a final assembly of 4,884,713 bases; wherein, 84 % of subreads mapped back to the assembly, resulting in consensus calling with an average base coverage of 114X. A GC skew analysis indicated that the origin of replication occurred at the 3,496,945^th^ position. This position was then renumbered as position 1 to indicate the origin of replication. The overall GC content of the genome was estimated to be 46 %, which is relatively higher than the GC content of genomes from *B. subtilis* strain 168*, B. megaterium* DSM 319*, B. thuringiensis* serovar kurstaki strain HD73 and *B. cereus* ATCC 14579 (Table 1). These bacteria were chosen for comparison because their complete genome sequences were available in the NCBI database. Nonetheless, the GC content correlated positively with the percent coding region, but no other major differences were noted, except that the number of tRNAs and rRNAs were relatively low in *Bacillus* sp. NRRL B-14911 (Table 1). The genomic sequence was annotated and submitted to the NCBI [GenBank Accn# CP006643].

**Table 1:** Comparative analysis of the genome of *Bacillus sp.* NRRL B-14911 with the genomes of other *Bacillus* species

Parameter	*Bacillus sp.* NRRL B-14911	*B. subtilis* strain 168	*B. megaterium* DSM 319	*B. thuringiensis* serovar kurstaki strain HD73	*B. cereus* ATCC 14579
Genome size (bp)	4884713	4215606	5097447	5646799	5411809
GC content (%)	46	43.5	38.1	35.2	35.3
Protein coding (bases)	4161286	3694614	4174023	4718007	4364840
Protein coding (%)	85.19	87.65	81.89	83.56	80.66
Number of genes	5179	4421	5272	6334	5501
Gene density (bp per gene)	943	953	967	892	987
tRNAs	85	86	115	104	108
rRNAs	27	30	33	36	39
Plasmids	1	-	-	7	1

Accession numbers: CP006643.1, *Bacillus* sp. NRRL B-14911; 225184640, *B. subtilis* strain 168; CP001982.1, *B. megaterium* DSM 319; CP003889.1, *B. thuringiensis* serovar kurstaki strain HD73; AE016877.1, *B. cereus* ATCC 14579

Analysis of the genomic sequence of *Bacillus* sp. NRRL B-14911 revealed several noteworthy features. (1) The bacterium was found to contain one large plasmid with a size of 144,911 bases [Table 1; KF831061, awaiting confirmation from the GenBank Accn#]. The plasmid encodes several proteins, including component of the type IV secretion system (a conserved large VirB4 domain protein; Additional file 1: Figure S2). (2) Base-modification analysis of the genome revealed two different motifs, one with methylation of adenine at the 6^th^ position to yield N6-methyladenine (m6A) on both the DNA strands, and the other on only one strand. The respective motifs are: CACNNNNNCTNG/CNAGNNNNNGTG (786/815 occurrences = 96.4 %; mean modification QV = 147.7) and GGAGT (4926/5958 occurrences = 82.7 %; mean modification QV = 134.7). We further scanned the chromosome and plasmid sequences using PacBio software to identify the restriction and modification systems that could be responsible for methylation at the specific motifs. While the genome did not reveal any of the above, the plasmid was found to contain a type I restriction-modification system specific for m6A modification. Although such modifications were suspected to have a role in pathway regulation [31], the role of the type I restriction-modification system in *Bacillus* sp. NRRL B-14911 has not been determined. (3) The genome encoded two vancomycin and one kanamycin resistance genes; however, the vancomycin resistance genes were found to be non-functional due to frameshifting insertions.

**Fig. 1:**
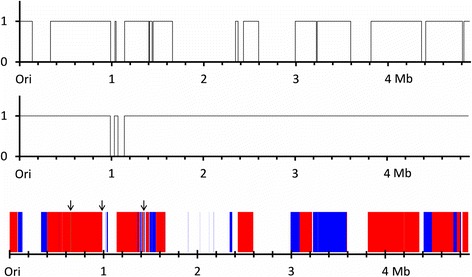
Comparison of assemblies of the *Bacillus sp.* NRRL B-14911 genome based on sequencing long-reads followed by *de novo* assembly as opposed to sequencing short-reads with subsequent scaffold building. Coverage maps depict short-read contigs assembled by scaffolding (top panel) and short-read contigs prior to scaffolding (middle panel) from a previous BioProject assembly of the *Bacillus* sp. NRRL-14911 genome, as aligned against our de novo assembly using long-reads. Alignment of short-read scaffolds and contigs is shown in the bottom panel. Red denotes 1X coverage by scaffolds, and blue denotes 1X coverage by the remaining unscaffolded contigs from the BioProject final assembly. Arrows point to areas with 2X coverage by sequences in their final contig list (visible as green sections in the online version with zoom)

Previous efforts to assemble the *Bacillus* sp. NRRL B-14911 genome using the 454 sequencing technology [Bioproject of Siefert, et al. 2000; GenBank accn# NZ_AAOX00000000] differed from our long-read sequencing approach [28]. The BioProject had sequences for a total of 74 contigs, consisting of 18 large and 56 small contigs (Fig. 1; and Additional file 1: Table S1). The 18 large contigs had coverage of 4,002,278 bases, accounting for 82 % of the total genomic length (Fig. 1). Eight of them, however, showed sequencing errors/ambiguity as denoted by ‘Ns’ in most places. Five of the 56 small contigs did not match our assemblies of either chromosome or plasmid (Additional file 1: Table S1). One of these (4281 bp; NZ_AAOX01000070) failed to match with any of the sequences in the non-redundant nucleotide database at NCBI (using BLASTN similarity search). Eight out of the remaining 51 small contigs matched only partially with our plasmid sequence, with coverage of 8 % (11,415 bases; Additional file 1: Table S1). Thus, the plasmid did not seem to be identified in their data set. Finally, among the remaining 43 small contigs, seven (<1.5 kb) matched our chromosome sequence, and the matching of another contig (NZ_AAOX01000091) was nearly perfect but fragmented in nine locations (Additional file 1: Table S1). Sequences of the remaining 35 small contigs matched with our genomic sequence. Comparison of our genome with the scaffold sequences derived in the bioproject also revealed several alterations in the orientation of the sequences. Overall, the total size of the genomic sequence submitted by Siefert et al. 2000 [28] was estimated to be 5,086,957 bases, 202,244 more bases than in our assembly (4,884,713 bases). This inflated genome size may be caused by many ‘Ns’ included in their sequences. The elimination of these Ns makes the relative coverage of their sequences (4,844,207 bases) to our genome to be 99.2 % (Fig. 1; Additional file 1: Figure S3), suggesting that their sequencing may possibly be near completion, but the sequences were fragmented and not assembled. Taken together, our sequencing approach using PacBio SMRT led to complete genome assembly with no errors, as also reported by others [29, 30]. However, heterogeneity within and across colonies generated from the same samples cannot be ruled out for the differences observed between the two approaches.

### Identification of the species for *Bacillus* sp. NRRL B-14911 as *B. infantis*

To identify the species of *Bacillus* sp. NRRL B-14911, we adopted four approaches: phylogenetic analyses of the 16S rRNA gene sequences, analysis of the 16S-23S rDNA ITS sequences, biochemical, and MALDI-TOF analyses.

#### Phylogenetic analyses of 16S rRNA gene sequences

**Fig. 2:**
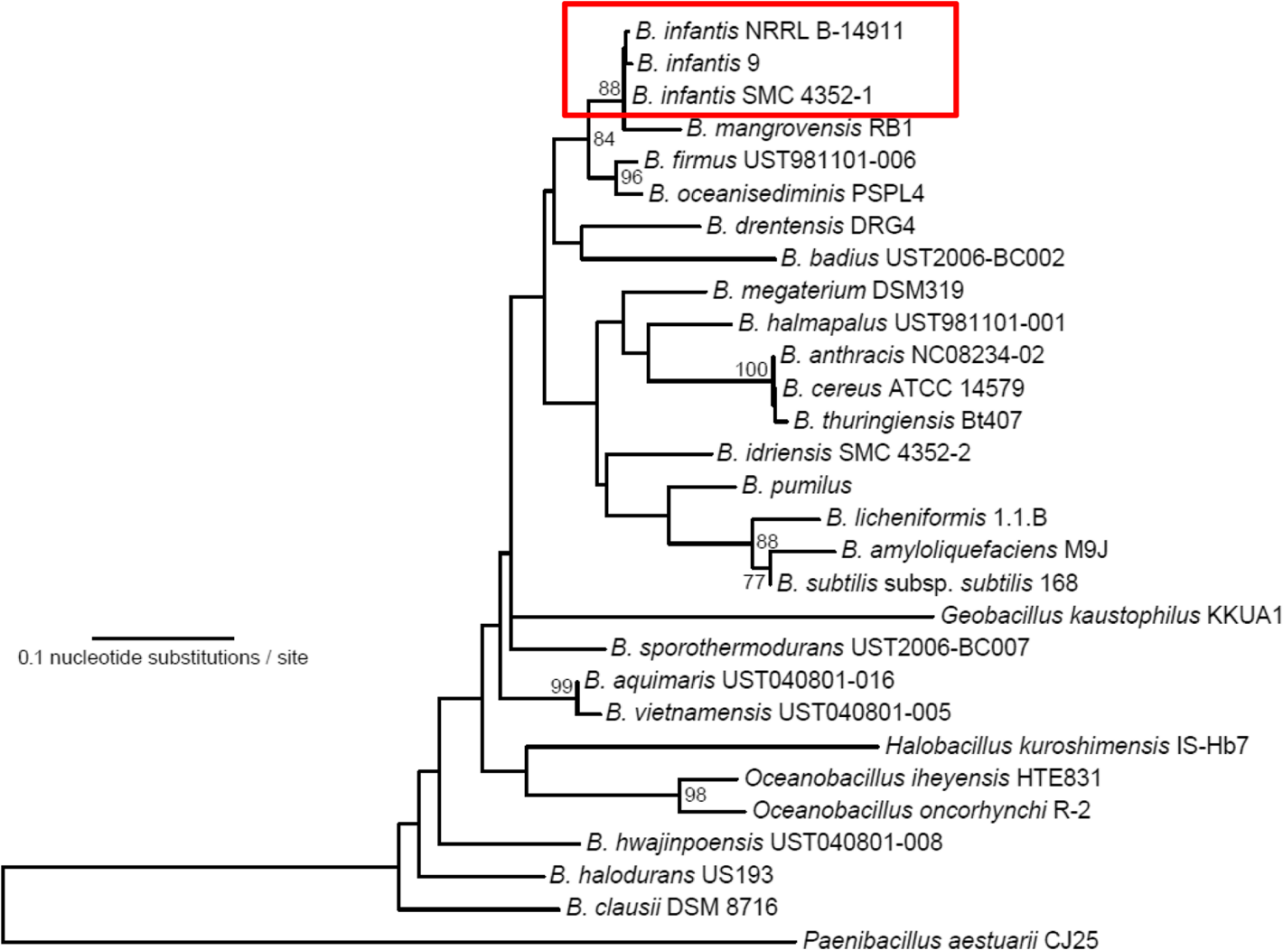
Construction of phylogenetic tree representing the 16S rRNA gene sequences derived from various *Bacillus* species. Bootstrap supporting values (%) are shown at the nodes only when higher than 70 %. Box indicates clustering of *Bacillus* sp. NRRL B-14911, *B. infantis* strain 9 and *B. infantis* strain SMC 43521-1 together in one clade

To determine the species-identity of *Bacillus* sp. NRRL B-14911, we performed phylogenetic analysis of the 16S rRNA gene sequences, a system that has been routinely used for speciating various bacteria [32–34]. We compared the 16S ribosomal DNA sequences of *Bacillus* sp. NRRL B-14911 with those from 28 selected species including *Bacillus* and four other related genera (*Halobacillus*, *Oceanobacillus*, *Geobacillus* and *Paenibacillus*) (Fig. 2). The phylogenetic analysis revealed that Bacillus sp. NRRL B-14911 formed a clade distinct from the soil-dwelling bacilli (Fig. 2). As expected, the marine inhabitants from the genera *Halobacillus*, *Oceanobacillus*, *Geobacillus* and *Paenibacillus* formed separate clades, further validating the reliability of using 16S rRNA gene sequence analysis for species identification (Fig. 2). Within the marine bacilli, *Bacillus* sp. NRRL B-14911 formed a well-supported cluster with two strains of *B. infantis* (*B. infantis* 9 and *B. infantis* SMC 4352-1) as well as *B. mangrovensis* (88 % bootstrap supporting value) indicating their close relationships (Fig. 2). *Bacillus* sp. NRRL B-14911 was particularly close to the two strains of *B. infantis* suggesting a possibility that *Bacillus* sp. NRRL B-14911 is likely to be *B. infantis*.

#### Sequence analysis of ITS regions

**Fig. 3:**
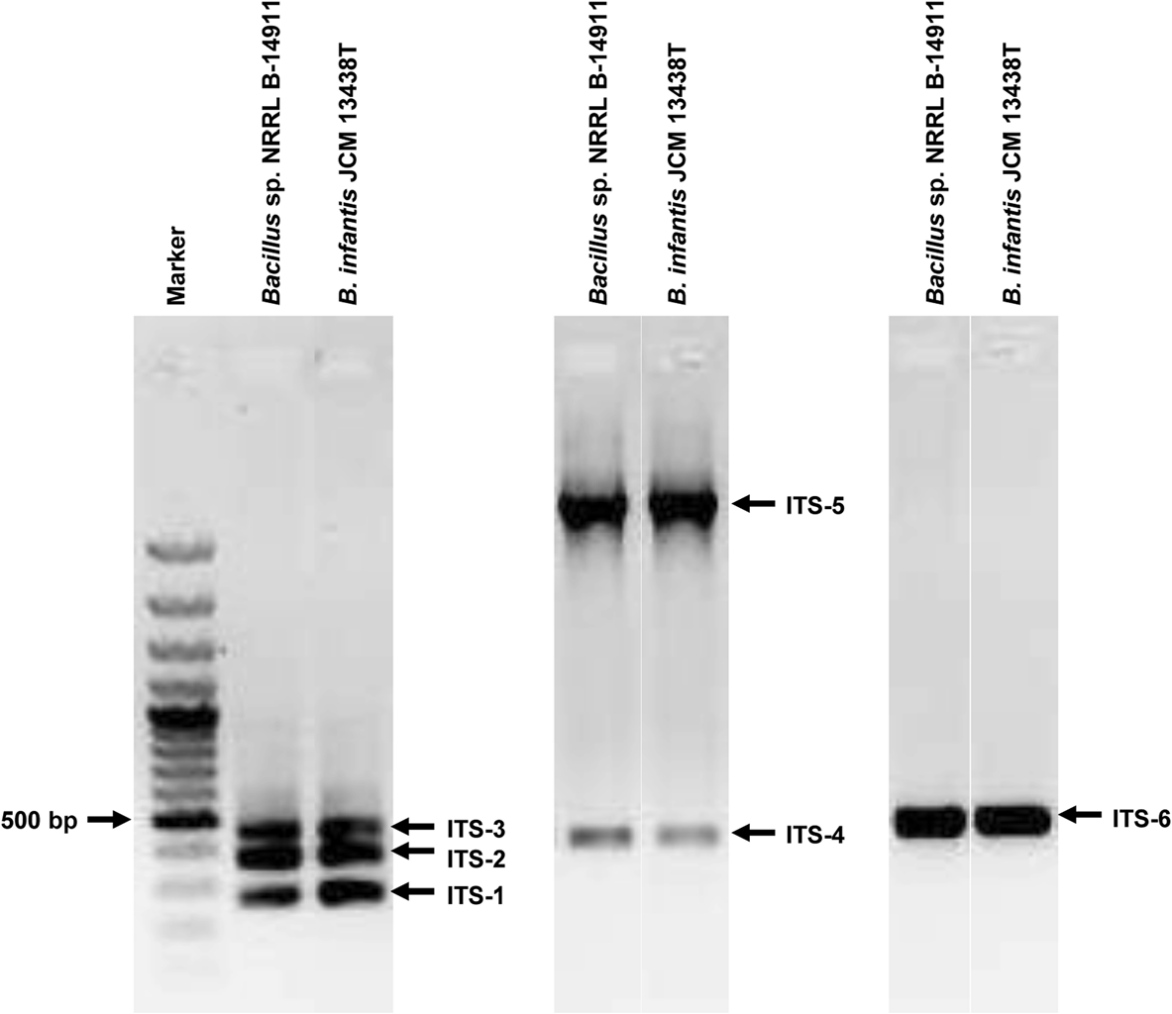
Comparative analysis of 16S-23S rDNA ITS of *Bacillus* sp. NRRL B-14911 and *B. infantis* JCM 13438 T. The ITS regions of *Bacillus* sp. NRRL B-14911 and *B. infantis* JCM 13438 T were amplified by PCR using the genomic DNA as a template as described in the Methods section. The PCR products were resolved in 1 % agarose gel electrophoresis and stained with ethidium bromide

Because that phylogenetic analysis suggested the probable species of *Bacillus* sp. NRRL B-14911 to be *B. infantis* JCM 13438 T, we sought to analyze the sequences of their ITS regions for further analysis. The 16S-23S rDNA ITS regions are considered to be the most variable regions of the ribosomal operon [17]. The sequences of these regions have been proposed to be species/strain-specific for the genus, *Bacillus* [17, 18]. Therefore, we performed a comparative evaluation of the ITS regions of both *Bacillus* sp. NRRL B-14911 and *B. infantis* JCM 13438 T to determine the similarities between the two. Using three sets of primers as previously described [17–19], we performed PCR analysis of genomic DNA from both the microorganisms. We named the resulting six amplicons as ITS1, ITS2, ITS3, ITS4, ITS5 and ITS6, and sequenced them. This analysis yielded the following information: (i) The sizes and patterns of PCR amplicons obtained from both *Bacillus* sp. NRRL B-14911 and *B. infantis* JCM 13438 T were similar (Fig. 3); (ii) By excluding the sequences of 16S and 23S rDNAs, we were able to determine the identities of five ITS-amplicons, ITS1, ITS2, ITS4, ITS5 and ITS6, and their sizes ranged from 151 to 268 bp (Table 2). These sequences also matched with the ITS regions of *Bacillus* sp. NRRL B-14911 [GenBank Accn# CP006643]. However, one amplicon, ITS3 did not yield ITS-specific information; and (iii) Comparisons of sequences of ITS1, ITS2, ITS4, ITS5 and ITS6 between *Bacillus* sp. NRRL B-14911 and *B. infantis* JCM 13438 T revealed their identities ranged from 96.7 to 100 % (Table 2). As the cut-off value for species identification based on ITS sequences has been suggested to be at least 92 % and the identities of both *Bacillus* sp. NRRL B-14911 and *B. infantis* JCM 13438 T meet this criterion, it is likely that both strains belong to the same species.

**Table 2:** Comparison of the sequence identities of the different ITS region of *Bacillus* sp. NRRL B-14911 and *B. infantis* JCM 13438 T

Patterns	Length of ITS (bp)	% Similarity
ITS-1	151	96.7
ITS-2	268	98.9
ITS-4	193	100
ITS-5	167	100
ITS-6	230	100

#### Biochemical analyses

To phenotype and compare the biochemical characteristics between *Bacillus* sp. NRRL B-14911 and *B. infantis* JCM 13438 T, we used Biolog PM (Biolog Omnilog) to analyze parameters, such as utilization of sugars and amino acids and other carbon sources, ability to grow at high salt concentrations, and growth in the presence of acids and antibiotics (Biolog Inc.). Biolog PM involves the reduction of tetrazolium compounds due to the utilization of a specific substrate in query under minimally defined nutrient conditions [20, 21]. PM analysis predicted the probable species of *B. infantis* JCM 13438 T and *Bacillus* sp. NRRL B-14911 to be the same using inoculation protocol A, the recommended protocol for *Bacillus*. The Omnilog software determined a final identification of *B. infantis* with a similarity index value (SIM) of 0.314 and 0.271 for *B. infantis* JCM 13438 T and *Bacillus* sp. NRRL B-14911 respectively. The next closest matches in the Omnilog database were *Brevibacterium otitidis* for *Bacillus* sp. NRRL B-14911; and *Staphylococcus equorum* subsp. *equorum* for *B. infantis* JCM 13438 T with SIM values of 0.314 and 0.126 respectively, which may not be the reliable identification for the genus *Bacillus*. It is to be noted that the SIM values are lower because of the recommend truncated incubation time used for *Bacillus*, where the automated ID software would normally incubate a protocol A 20 h or more. Further, by comparing the biochemical characteristics of *Bacillus* sp. NRRL B-14911 with *B. infantis* JCM 13438 T [35], we noted that most of the parameters of *Bacillus* sp. NRRL B-14911 complemented those of *B. infantis* JCM 13438 T (Table 3). Likewise, by examining metabolism in the GENIII plate containing various antimicrobials, we noted that both the bacteria were likely susceptible to vancomycin, troleandomycin, lincomycin, and a few tetrazolium compounds. Sensitivity to vancomycin was further confirmed with disc diffusion testing and it supports the finding of the genomic sequence analysis which also found the corresponding gene to be non-functional as described above.

**Table 3:** Comparison of biochemical characteristics of *Bacillus* sp. NRRL B-14911 with *B. infantis* JCM 13438 T

Biochemical Test	*Bacillus* sp. NRRL B-14911	*B. infantis* JCM 13438 T^a^
α-D-Glucose	+	+
D-Mannose	+/-	+/-
D-Mannitol	+	+
D-Maltose	+	+
D-Melbiose	+	+
D-Fructose	+	+
D-Trehalose	+	+
D-Galactose	+	+
Sorbitol	**-**	**-**
Inulin	**-**	+/-
Esculin	+/-	+
Glycerol	+/-	+/-
Cellobiose	+/-	+
Gentiobiose	+	+
Sucrose	+	+
Oxidase	-	-
Catalase	+	+
D-Raffinose	+/-	+/-
Gelatin	-	-
Pectin	+	+
p-hydroxy-phenylacetic acid	-	-
Tween 40	-	+/-
Dextrin	+	+
α-D-Lactose	+/-	+
Glylcyl-L-Proline	-	-
Methyl Pyruvate	-	-
γ-amino-butyric acid	-	-
D- Arabitol	-	-
L-Alanine	-	-
D-Lactic Acid Methyl Ester	-	-
α-Hydroxy Butyric Acid	-	+/-
β-Methyl-D-Glucoside	+	+/-
myo-Inositol	-	-
L-Arginine	-	-
D-Glucononic Acid	+	+
β-hyrdoxy D, L, Butyric Acid	-	-
D-Salicin	+/-	+/-
L-Aspartic Acid	-	-
D-Glucuronic Acid	+	+
Citric Acid	-	-
α-Keto-Butyric Acid	-	-
D-Fucose	+/-	+
D-Glucose-6-PO4	+	+
Glucuronamide	+	+
α-Keto Glutaric Acid	-	-
Acetoacetic Acid	+	+/-
N-Acetyl-β-D Mannosamine	-	-
L-Fucose	+	+
D-Fructose-6-PO4	+	+
L-Histidine	-	-
Mucic Acid	+/-	+/-
D-Malic Acid	-	-
Propionic Acid	+/-	-
D-Turanose	+/-	+
N-Acetyl-D-Galactosamine	-	-
L-Rhamnose	+/-	+
D-Aspartic Acid	-	-
L-Pyroglutamic Acid	-	-
Quinic Acid	-	-
L-Malic Acid	-	-
Acetic Acid	+/-	+/-
N-Aceytl Neuraminic Acid	-	-
Inosine	-	+/-
D-serine	-	-
L-Serine	-	-
D-saccharic acid	-	-
Bromo-Succinic Acid	-	-
Formic Acid	-	-
1 % NaCl	+	+
1 % Sodium Lactate	+	+
Troleandomycin	-	-
Lincomycin	-	-
Vancomycin	-	-
Nalidixic Acid	+/-	-
Aztreonam	+	+
ph 6.0	+	+
4 % NaCl	+	+
Fusidic Acid	-	-
Rifamycin SV	-	-
Guanidine HCL	+/-	+/-
Tetrazolium Violet	+/-	+/-
Lithium Chloride	+	+
Sodium Butyrate	+	+
pH 5.0	-	-
8 % NaCl	+/-	+/-
Minocycline	-	-
Niaproof 4	-	-
Tetrazolium Blue	+/-	+/-
Potassium Tellurite	+	+
Sodium Bromate	-	-

+, present; -, absent; ^a^,reference organism

#### MALDI-TOF analysis

MALDI-TOF analysis has been widely used to discriminate bacteria at genus, species, subspecies and strain levels [23, 36–38]. MALDI-TOF analysis was conducted on the *Bacillus* sp. NRRL B-14911 proteome using Bruker Daltonik MALDI Biotyper. The analysis predicted the bacterium to be *B. infantis* after four independent analysis with scores 2.137, 2.139, 2.228, 2.097. The second closest matches in the database were *B. nealsonii* and *B. firmus*. To further validate this finding, we repeated the MALDI-TOF analysis using proteomes from both *Bacillus* sp. NRRL B-14911 and *B. infantis* JCM 13438 T. These analyses predicted both the bacteria to be *B. infantis* with scores ranging from 1.883 to 2.065 for *Bacillus* sp. NRRL B-14911, and 1.809 to 2.024 for *B. infantis* JCM 13438 T suggesting that their proteomic profiles are similar.

Little is known about the diversity within the species of *B. infantis* as only a very small number of isolates have been described. As the data generated with both biochemical and MALDI-TOF analyses agreed with the phylogenetic analysis, but the sequence analyses of ITS regions revealed identities in the range of 96.7 % to 100 %, we believe that *Bacillus* sp. NRRL B-14911 may represent strain variation within the species *B. infantis.* Thus, we suggest the species and strain of *Bacillus* sp. NRRL B-14911 as *B. infantis* NRRL B-14911.

### Analysis of virulence factors

**Fig. 4:**
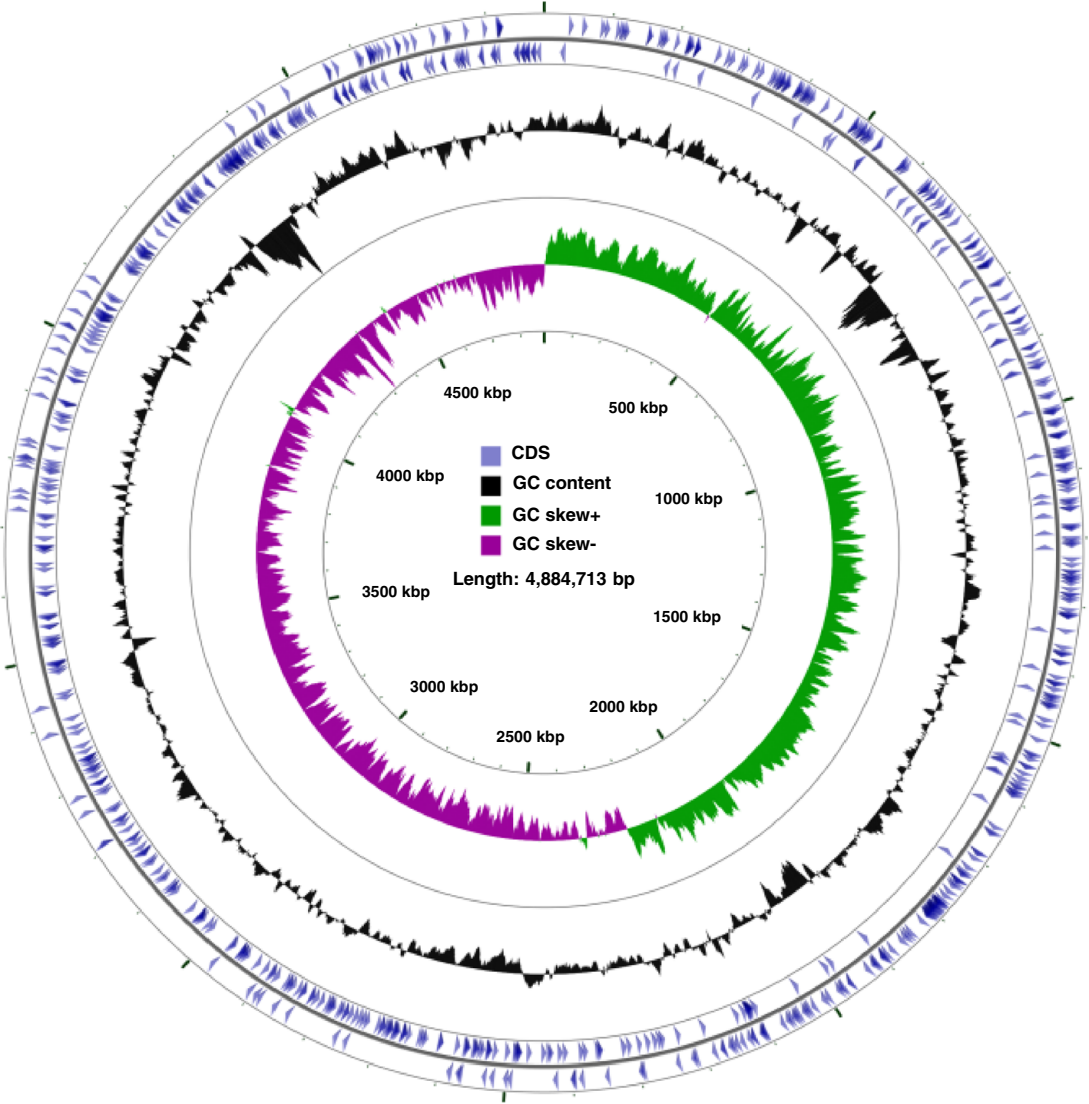
Circular genome map of *Bacillus* sp. NRRL B-14911 showing the location of genes for virulence factors. The map shows the locations of 623 genes of *Bacillus* sp. NRRL B-14911 potentially encoding virulence factors. The two outer blue circles represent the genes for virulence factors shown in forward and reverse directions. The innermost circle represents the GC skewness, and the second innermost circle represents the GC content of the *Bacillus* sp. NRRL B-14911 genome. The coordinates of each gene are listed in Additional file 1: Table S2. The start and end positions in Additional file 1: Table S2 match with the location of genes in Fig. 4

The ability of bacteria to cause disease in susceptible hosts is determined largely by their virulence factors. We attempted to identify the genes from *B. infantis* NRRL B-14911 that encode for various virulence factors based on the sequence similarities with virulence factor proteins found in the VFDB database. A total of 623 proteins from *Bacillus* sp. NRRL B-14911 were identified to be potential virulence factors. A list of these proteins and their gene locations are shown in Additional file 1: Table S2 and Fig. 4. Among these, 18 proteins were found to be unique to *Bacillus* sp. NRRL B-14911 when compared with those from pathogenic (*B. anthracis* str. Ames, *B. cereus* ATCC 14579, and *B. licheniformis* ATCC 14580) and non-pathogenic bacilli (*B. pseudofirmus* OF4, *B. amyloliquefaciens* subsp. plantarum UCMB5033, *B. subtilis* subsp. subtilis str. OH 131.1) (Additional file 1: Table S3 and Additional file 1: Figure S4). Similarly, by comparing with the pathogenic bacilli alone, we noted that 225 genes to be common for both *Bacillus* sp. NRRL B-14911 and the pathogenic bacilli (Additional file 1: Table S4 and Additional file 1: Figure S5). The notable virulence factor genes include intercellular adhesion protein, invasion-associated protein, accessory colonization factor, laminin-binding surface protein, toxin co-regulated pilus biosynthesis protein, transporters, and regulatory proteins PhoP/PhoQ.

### Other pathways

We evaluated the genome of *Bacillus* sp. NRRL B-14911 for the presence or absence of methyltransferases, transporters, enzymatic and biochemical pathways and IS elements and compared the results with those from four other bacilli: *B. subtilis* strain 168, *B. megaterium* DSM 319, *B. thuringiensis* serovar kurstaki strain HD73 and *B. cereus* ATCC 14579.

#### Methyl transferases and transporters

In prokaryotes, DNA-methylation controls a number of physiological processes, including transcription, DNA mismatch repair and initiation of replication. Three classes of methyltransferases have been identified in bacteria: the first two classes transfer a methyl group from S-adenosyl-L-methionine (SAM) to adenine and cytosine to yield m6A and N4-methylcytosine (m4C), respectively; and the third class transfers a methyl group from SAM to cytosine to generate 5-methylcytosine (m5C) [39]. Five methyl transferases were found to be present uniquely in *B. infantis* NRRL B-14911. These include 50S rRNA methyltransferase, lysine methyltransferase, N5-glutamine SAM-dependent methyltransferase, protein-L-isoaspartate O-methyltransferase, and SAM-dependent methyltransferase (Additional file 1: Table S5). It is reported that rRNA methyltransferases confer antibiotic resistance to the bacteria by adding methyl groups specifically to the 23S rRNA, and prevent binding of drugs/antibiotics to the large subunit of the ribosome [40]. Thus, bacteria like *B. infantis* NRRL B-14911 that possess 50S rRNA methytransferases may have a survival advantage under antibiotic selection pressure. Similarly, lysine methyltransferases are known to mediate methylation of lysine residues in ribosomal and flagellar proteins and have a role in the posttranslational modification processes [41].

Two main superfamilies of transporters have been identified in bacteria. These include ion-coupled transporters and the ABC solute ATPases, which maintain in- and out-flow of nutrients and wastes. We noted a number of transporters present in *Bacillus* sp. NRRL B-14911 (Additional file 1: Table S6). A few unique transporters include (1) antibiotic ABC transporter ATP-binding protein and arsenic transporter ATPase/arsenite efflux transporter, which determine resistance to antibiotics and arsenic by extrusion [42, 43]; (2) C4-dicarboxylate ABC transporter, a tripartite ATP-independent periplasmic transporter that transports organic acids like succinate, malate fumarate, keto-acids and N-acetyl neuraminic acid [44]; (3) corrinoid ABC transporter that facilitates the intake of complex cyclic tetrapyrrole molecules such as hemes, chlorophylls and coenzyme F430 [45], (4) macrolide transporter, an efflux transporter of macrolide drugs like erythromycin and azithromycin, which determines resistance to antibiotics [46], (5) nicotinamide riboside transporter that aids in the uptake of nicotinamide riboside into the cytoplasm [47, 48]; (6) nitrate ABC transporter that mediates uptake of nitrate into the cell [49]; (7) peptide ABC transporter, which is often present in firmicutes, that determines resistance to antimicrobial peptides by substrate extrusion from the cell [50]; and (8) riboflavin transporter fmnp, which is involved in the uptake of riboflavin into the cell and a frequently occurring transporter in firmicutes [51].

#### Enzymes and biochemical pathways

**Fig. 5:**
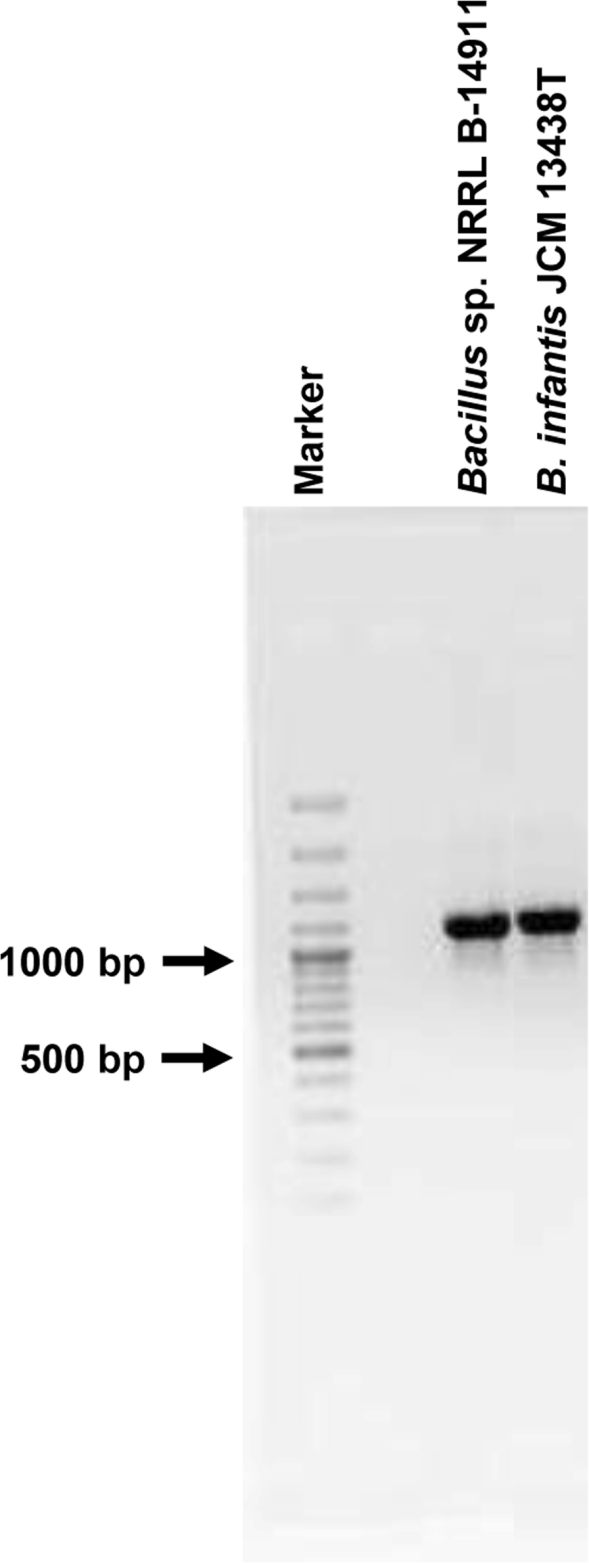
Conservation of AAH gene in *Bacillus* sp. NRRL B-14911 and *B. infantis* JCM 13438 T. AAH gene was amplified from *Bacillus* sp. NRRL B-14911 and *B. infantis* JCM 13438 T using genomic DNA as a template by PCR. The PCR amplicons were resolved in 1 % agarose gel electrophoresis and stained with ethidium bromide

As described above, *Bacillus* sp. NRRL B-14911 contains a mimicry epitope (BAC 25-40; EGFTRLSFTAEEKAAH) for cardiac myosin peptide (Myhc-α 334-352; DSA**F**DV**LSFTAEEKA**GVYK) (identical residues are bolded), with allantoate amidohydrolase (AAH) as the source protein [12]. As expected, the annotated gene sequence of AAH also contains the exact amino acid sequence of the mimicry epitope as indicated above [GenBank protein ID: AGX06322]. To further confirm whether *B. infantis* JCM 13438 T also contains the gene for AAH, and if so, whether the sequence for mimicry epitope BAC 25-40 is conserved, we amplified the AAH gene from both *Bacillus* sp. NRRL B-14911 and *B. infantis* JCM 13438 T using the genomic DNA as a template, and sequenced the PCR products. These analyses revealed the presence of AAH gene in *B. infantis* JCM 13438 T (Fig. 5), and the amino acid sequence of the mimicry epitope, BAC 25-40 was also conserved except one silent mutation (GAG in place of GAA for glutamic acid, E; Additional file 1: Table S7). Previously, we had reported the conservation of the mimicry epitope, BAC 25-40 in various other *Bacillus* species [12]. Functionally, the biochemical reaction carried out by AAH is a two-step conversion of allantoate to ureidoglycolate and ammonia [52], and AAH functionality has been detected in both plants and bacteria. It is possible that the AAH gene may have been laterally transferred between plants and bacteria for recycling nitrogen [52, 53]. We also noted that *B. infantis* NRRL B-14911 is capable of biosynthesizing LPS and steroids (Additional file 1: Table S8). Additionally, as reported by others [54], our sequence analysis revealed the presence of a novel class of extracellular poly (3-hydroxybutyrate) (PHB) depolymerase. This enzyme is required for degradation of PHB to produce 3-hydroxybutyrate as an intracellular carbon and energy source under conditions of limited or unbalanced nutrient-availability [54]. We speculate that the PHB depolymerase may be critical for bacterial survival in the environment.

#### IS elements

The IS elements are recombinationally active, mobile, genetic segments of bacterial DNA (600 to 3000 bp) that move from one position to another within the same chromosome or to a different chromosome. One consequence could be inactivation of gene expression if the insertion of IS elements takes place within the coding sequence [55, 56]. We noted that IS*1*, IS*1380*/IS*942*, IS*5*/IS*903* and Tn*3* are uniquely present in the genome of *B. infantis* NRRL B-14911 (Additional file 1: Table S9) compared to other bacilli. Previously, it was demonstrated that the transposon Tn*3* (4957 bp) carries the enzyme β-lactamase, in addition to transposase and resolvase, and confers resistance to β-lactam antibiotics [57, 58]. Whether the Tn*3* present in *B. infantis* NRRL B-14911 perform similar functions requires additional studies.

## Conclusions

In summary, we have described the complete genome sequence analysis of a marine microbe called *Bacillus* sp. NRRL B-14911. The bacterial genome sequence analysis allowed us to identify genes for a wide range of virulence factors and enzymatic and biochemical pathways, including IS elements that are distinct from other closely related bacilli. The availability of the complete genomic sequence of *Bacillus* sp. NRRL B-14911 may thus provide opportunities to genetically manipulate its genome to study the genes in bacterial survival and virulence. Furthermore, phylogenetic and 16S-23S rDNA ITS sequence analyses including biochemical and phenotypic characterizations suggested a close-association with *B. infantis,* and as such, we have proposed the species and strain of *Bacillus* NRRL B-14911 as *B. infantis* NRRL B-14911. Of note, *B. infantis* was previously identified as one of the six bacterial isolates from a newborn child with sepsis, but its pathological significance was unknown [35]. Similarly, a variety of pathogens have been implicated in the causation of heart autoimmunity, but their direct causal links remain tenous clinically. Thus, it becomes difficult to explain the persistent inflammation in the absence of detectable infectious particles. In these circumstances, autoimmunity is suspected with a challenge being able to prove the cause and effect relationship. Mechanistically, break in self-tolerance as a result of exposure to microbes carrying mimicry epitopes for self-antigens like cardiac myosin can lead to heart autoimmunity through the generation of cross-reactive T cells as we have demonstrated for BAC 25-40 present in *Bacillus* spp. NRRL B-14911 ([12], Additional file 1: Table S7 and Fig. 5). This bacterium, may thus serve as a useful tool to establish a disease model that permits systematic analysis of autoimmune events with respect to the appearance, disappearance, persistence, and/or reappearance of cross-reactive T cells and their functionalities experimentally in susceptible rodent strains.

## Abbreviations

AAH, allantoate amidohydrolase; BLASTN, Basic Local Alignment Search Tool; BLASTP, Basic Local Alignment Search Tool; DCM, dilated cardiomyopathy; HF, heart failure; IS, insertion sequence; ITS, intergenic transcribed spacers; m4C, N4-methylcytosine; m5C, 5-methylcytosine; MALDI-TOF, matrix-assisted laser desorption ionization time-of-flight; MS, mass spectrometry; Myhc, cardiac myosin heavy chain; PHB, poly (3-hydroxybutyrate); PM, phenotypic microarray; rRNA, ribosomal RNA; SAM, S-adenosyl-L-methionine; VFDB, Virulence Factor Database

## Additional file

Additional file 1: Figure S1. Identification of Bacillus sp. NRRL B-14911 spores. Bacterial smear was stained with malachite green and safranin as described in the Methods section and examined under oil immersion microscope. Arrows indicate round and symmetrical endospores present both within and outside the bacteria. Original magnification: 100x. **Figure S2.** The genes encoded by the plasmid of Bacillus sp. NRRL B-14911. The plasmid sequence was generated from the sequence data obtained by sequencing of the genomic DNA from Bacillus sp. NRRL B-14911 in long-reads. The genes encoded by the plasmid were annotated as described for the bacterial chromosome (see methods).**Figure S3.** Alignment of contigs previously reported for Bacillus sp. NRRL B-14911 to the new long-read-based finished assembly. The inner ring (blue) represents contigs assembled from short-reads without any scaffolding (middle panel in Figure 1) as aligned to our de novo assembly based on long-reads. The outer ring (pink and black) represents alignment of contigs after scaffolding (top and bottom panels in Fig. 1). Note, white lines and blocks show large areas without any coverage in the prior assembly. Designations for each scaffold and contig are derived from GenBank accession numbers, which are abbreviated for convenience in display. Scaffolds are shown in pink and have full accessions with the format CH6723XX, where XX is the number shown on the figure following “CH”. Black- and blue-shaded regions represent un-scaffolded contigs, which have full accessions with the format AAOX01000XYY, where X is either 0 (for YY between 23 and 99) or 1 (for YY between 00 and 09). Only contigs with ≥ 99.900 % identity are shown. The scale in the middle of the circle is based on the finished de novo assembly, made using long-read sequencing. **Figure S4.** Circular genome map of Bacillus sp. NRRL B-14911 showing the location of genes for virulence factors that are unique to this bacterium in relation to other Bacillus. The circular map of Bacillus sp. NRRL B-14911 shows the locations of 18 virulence factor genes that are unique to this bacterium as determined by comparing the genomes of three pathogenic, and three non-pathogenic bacteria as described in the ‘Methods’ section. The two outer blue circles represent the genes for virulence factors present in forward and reverse directions. The innermost circle represents the GC skewness, and the second innermost circle represents the GC content of the Bacillus sp. NRRL B-14911 genome. The coordinates of each gene are listed in Additional file 1: Table S3. The start and end positions in Additional file 1: Table S3 match with the location of genes in Additional file 1: Figure S4. **Figure S5.** Circular genome map showing the location of virulence factor genes common to both Bacillus sp. NRRL B-14911 and other pathogenic bacilli. The circular map shows the locations of 225 virulence factor genes that are common to both Bacillus sp. NRRL B-14911 and three other pathogenic bacilli. Their genome comparisons were made as described in the ‘Methods’ section. The two outer blue circles represent the genes for virulence factors shown in forward and reverse directions. The innermost circle represents the GC skewness, and the second innermost circle represents the GC content of the Bacillus sp. NRRL B-14911 genome. The coordinates of each gene are listed in Additional file 1: Table S4. The start and end positions in Additional file 1: Table S4 match with the location of genes in Additional file 1: Figure S5. **Table S1.** Comparison of the issues with sequencing by scaffolds in the bioproject in relation to our method of sequencing by long-reads. **Table S2.** List of genes for virulence factor encoded in the genome of Bacillus sp. NRRL B-14911. **Table S3.** List of unique genes for virulence factor encoded in the genome of Bacillus sp. NRRL B-14911. **Table S4.** List of genes for virulence factor encoded in the genome of Bacillus sp. NRRL B-14911 that are in common with pathogenic Bacilli. **Table S5.** Comparative analysis of the presence or absence of common methyltransferases between Bacillussp. NRRL B-14911 and other Bacillus species. **Table S6.** Comparative analysis of the presence or absence of transporters between Bacillus sp. NRRL B-14911 and other Bacillus species. **Table S7.** Comparison of the nucleotide sequences and amino acid sequences corresponding to the epitope BAC 25-40 from Bacillus sp. NRRL B-14911and B. infantis JCM 13438 T. **Table S8.** Comparative analysis of the presence or absence of enzymes and biochemical pathways of Bacillus sp. NRRL B-14911 in relation to other Bacillus species. **Table S9.** Comparison of insertion sequence elements between Bacillus sp. NRRL B-14911 and other Bacillus species. (PDF 2089 kb)
