# Supplementary material for: Whole genomic sequence analysis of Bacillus infantis: defining the genetic blueprint of strain NRRL B-14911, an emerging cardiopathogenic microbe
Source: BMC Genomics. 2016 Aug 22;17(Suppl 7):511. doi: 10.1186/s12864-016-2900-2 (PMC5001198; doi:10.1186/s12864-016-2900-2)
Supplement: Additional file 1: Figure S1. — Identification of Bacillus sp. NRRL B-14911 spores. Bacterial smear was stained with malachite green and safranin as described in the Methods section and examined under oil immersion microscope. Arrows indicate round and symmetrical endospores present both within and outside the bacteria. Original magnification: 100x. Figure S2. The genes encoded by the plasmid of Bacillus sp. NRRL B-14911. The plasmid sequence was generated from the sequence data obtained by sequencing of the genomic DNA from Bacillus sp. NRRL B-14911 in long-reads. The genes encoded by the plasmid were annotated as described for the bacterial chromosome (see methods).Figure S3. Alignment of contigs previously reported for Bacillus sp. NRRL B-14911 to the new long-read-based finished assembly. The inner ring (blue) represents contigs assembled from short-reads without any scaffolding (middle panel in Figure 1) as aligned to our de novo assembly based on long-reads. The outer ring (pink and black) represents alignment of contigs after scaffolding (top and bottom panels in Fig. 1). Note, white lines and blocks show large areas without any coverage in the prior assembly. Designations for each scaffold and contig are derived from GenBank accession numbers, which are abbreviated for convenience in display. Scaffolds are shown in pink and have full accessions with the format CH6723XX, where XX is the number shown on the figure following “CH”. Black- and blue-shaded regions represent un-scaffolded contigs, which have full accessions with the format AAOX01000XYY, where X is either 0 (for YY between 23 and 99) or 1 (for YY between 00 and 09). Only contigs with ≥ 99.900 % identity are shown. The scale in the middle of the circle is based on the finished de novo assembly, made using long-read sequencing. Figure S4. Circular genome map of Bacillus sp. NRRL B-14911 showing the location of genes for virulence factors that are unique to this bacterium in relation to other Bacillus. The circular map of Bacillus sp [file 12864_2016_2900_MOESM1_ESM.pdf]

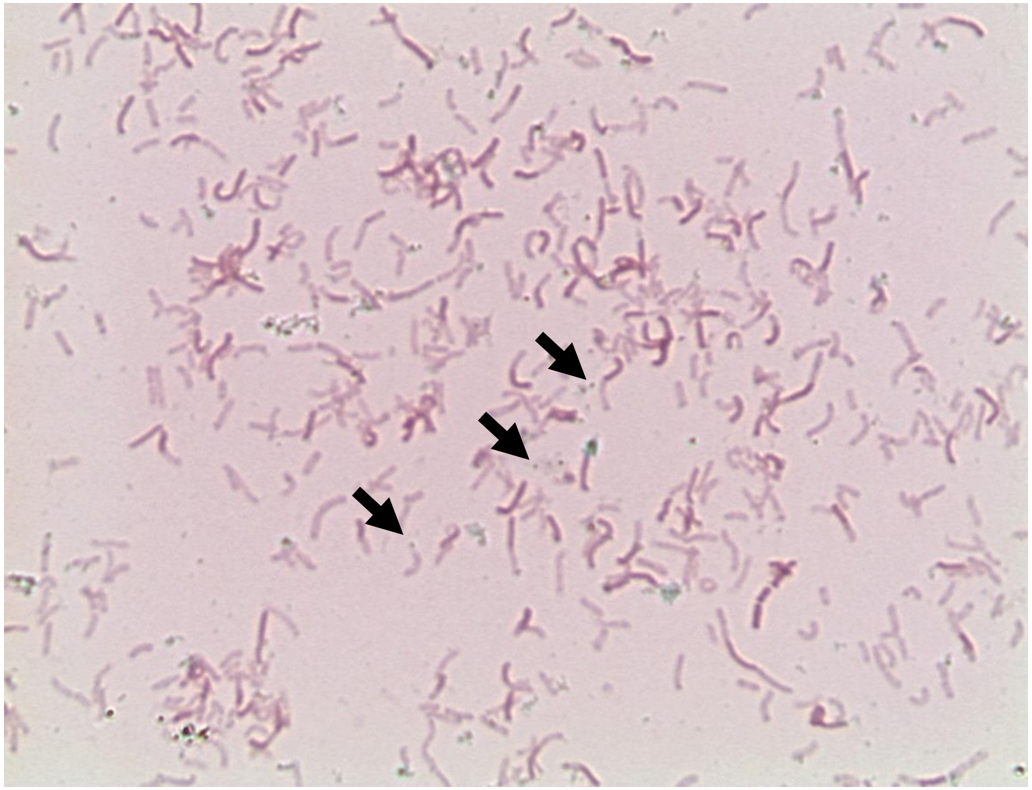

Fig. S1

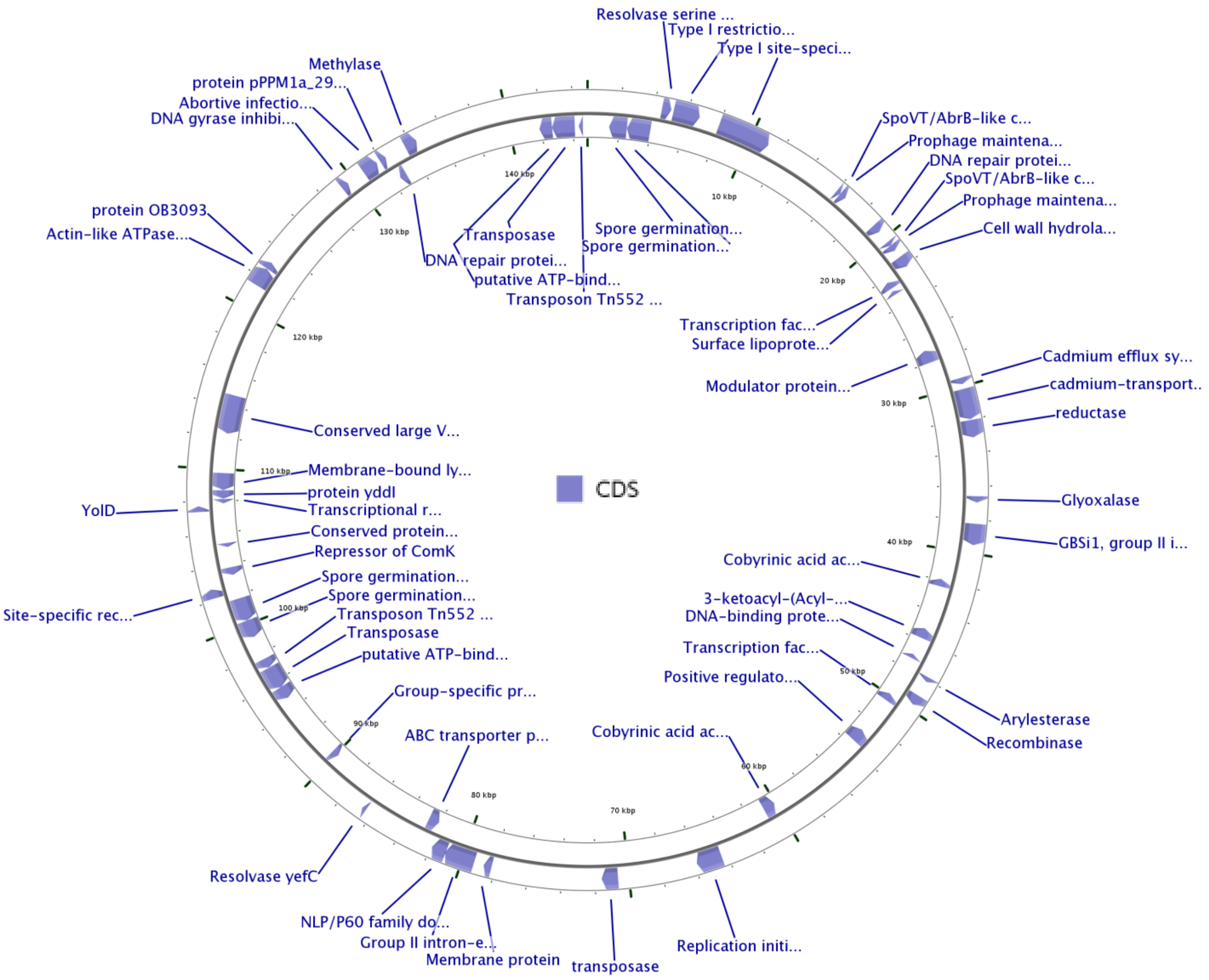

Resolvase serine ...
Type I restrictio...
Type I site-speci...
SpoVT/AbrB-like c...
Prophage maintena...
DNA repair protei...
SpoVT/AbrB-like c...
Prophage maintena...
Cell wall hydrola...
Cadmium efflux sy...
cadmium-transport..
reductase
Glyoxalase
GBSi1, group II i...
Arylesterase
Recombinase
Replication initi...
transposase
Membrane protein
Group II intron-e...
NLP/P60 family do...
Resolvase yefC
Site-specific rec...
YolD
Actin-like ATPase...
protein OB3093
DNA gyrase inhibi...
Abortive infectio...
protein pPPM1a_29...
Methylase
Transposase
DNA repair protei...
putative ATP-bind...
Transposon Tn552 ...
Spore germination...
Spore germination...
Transcription fac...
Surface lipoprote...
Modulator protein...
Cobyrinic acid ac...
3-ketoacyl-(Acyl-...
DNA-binding prote...
Transcription fac...
Positive regulato...
Cobyrinic acid ac...
ABC transporter p...
Group-specific pr...
putative ATP-bind...
Transposase
Transposon Tn552 ...
Spore germination...
Spore germination...
Repressor of ComK
Conserved protein...
Transcriptional r...
protein yddl
Membrane-bound ly...
Conserved large V...
CDS
10 kbp
20 kbp
30 kbp
40 kbp
50 kbp
60 kbp
70 kbp
80 kbp
90 kbp
100 kbp
110 kbp
120 kbp
130 kbp
140 kbp

Fig. S2

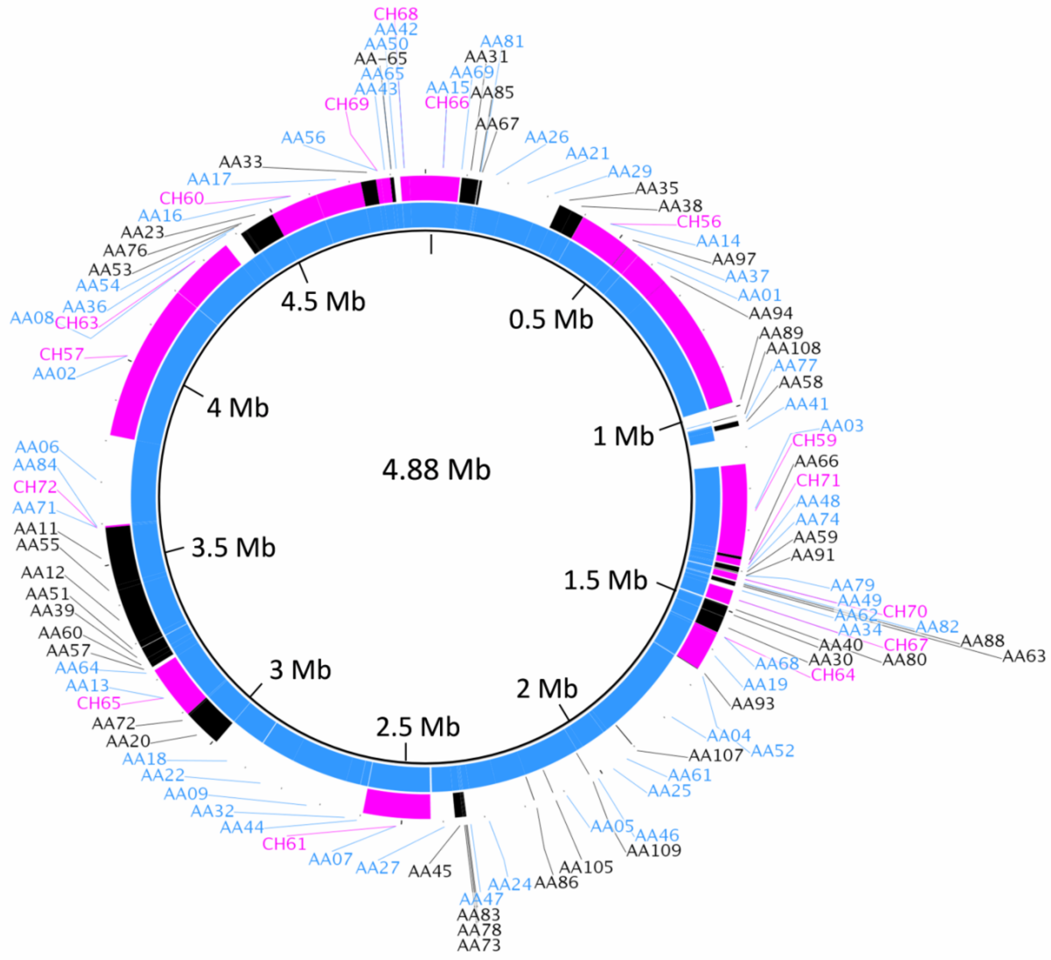

Fig. S3

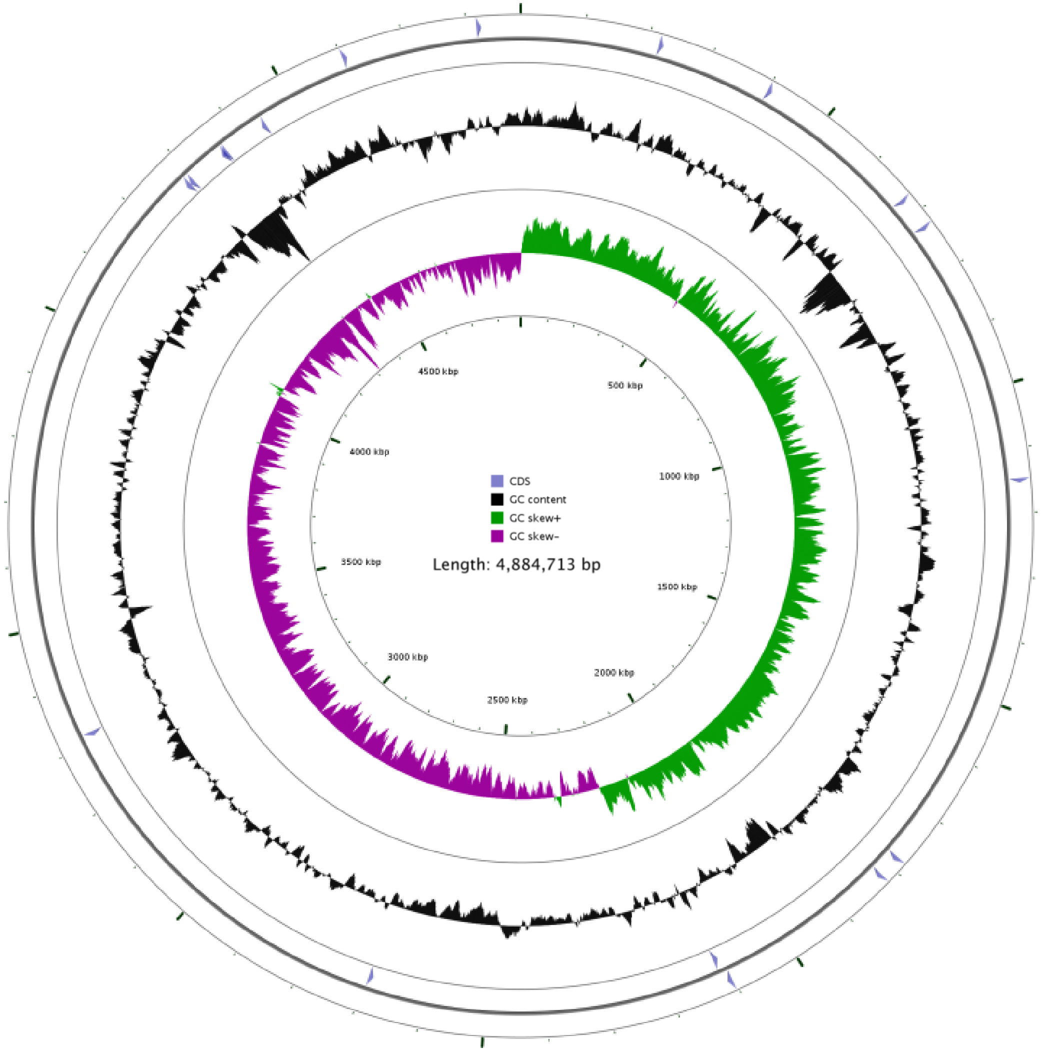
CDS
GC content
GC skew+
GC skew-
Length: 4,884,713 bp
500 kbp
1000 kbp
1500 kbp
2000 kbp
2500 kbp
3000 kbp
3500 kbp
4000 kbp
4500 kbp

Fig. S4

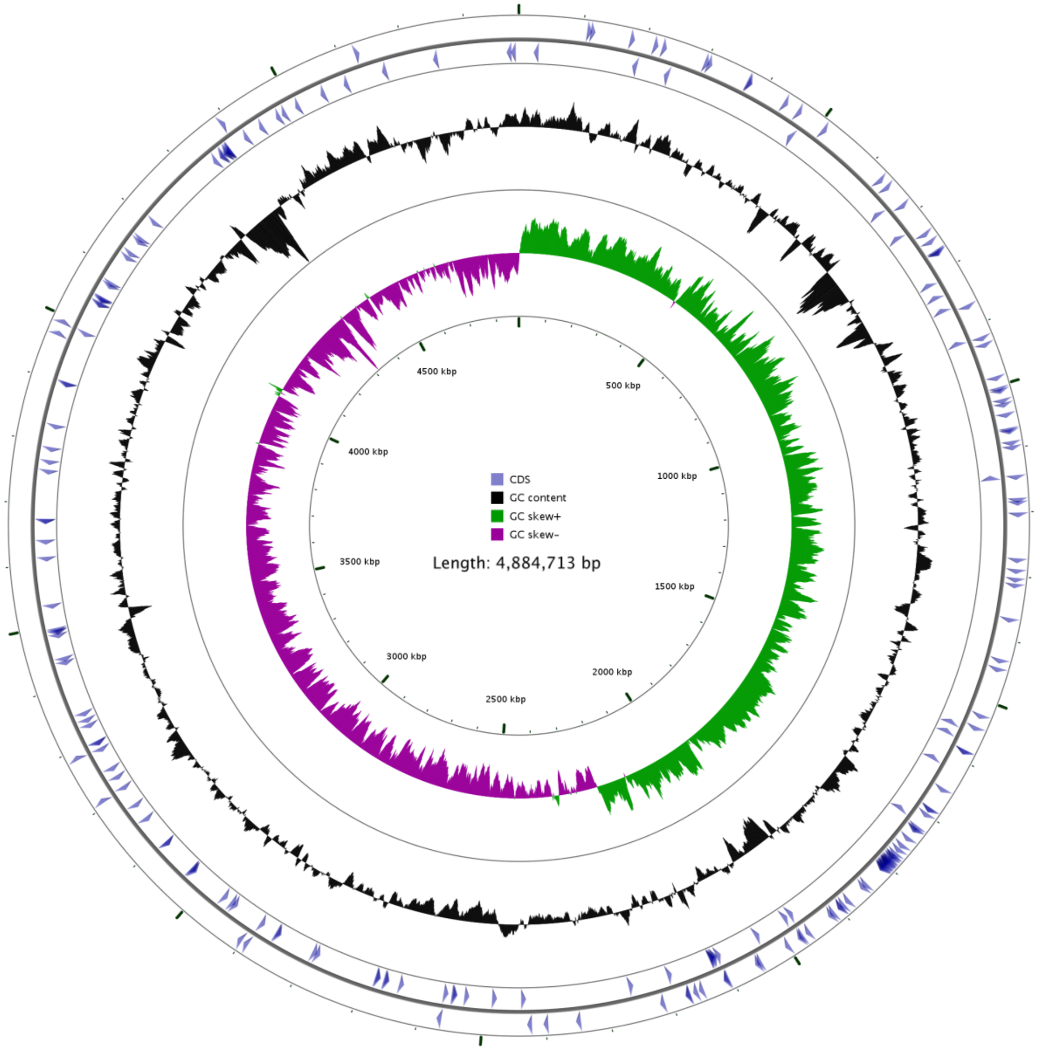
CDS
GC content
GC skew+
GC skew-
Length: 4,884,713 bp
500 kbp
1000 kbp
1500 kbp
2000 kbp
2500 kbp
3000 kbp
3500 kbp
4000 kbp
4500 kbp

Fig. S5

Table S1: Comparison of the issues with sequencing by scaffolds in the bioproject in relation to our method of sequencing by long-reads.

	Accession number	Issue in relation to sequencing by long-reads	Length of contigs
6288	CH672372	inversion noted	684932
19130	CH672371	subcontig segments ordered incorrectly	635020
22021	CH672370	inversion noted	606964
34806	CH672369		395516
37930	CH672368	subcontig segments ordered incorrectly	319574
46932	CH672367	subcontig segments ordered incorrectly	227880
127381	CH672366		222899
143074	CH672365		209159
149968	CH672364	50 kb on 3' end no match plasmid or chromosome or GenBank	208649
208649	CH672363		149968
209159	CH672362		146055
222899	CH672361		143074
227880	CH672360		142379
319574	CH672359		134330
395516	CH672358	two inversions	127381
606964	CH672357		91393
635020	CH672356	inversion noted	72665
684932	CH672355		46932
1004	AAOX01000109	match CH55	39783
1029	AAOX01000108	match CH56	39271
1033	AAOX01000107	match CH55	37930
1039	AAOX01000106	no match	37188
1041	AAOX01000105	match CH55	34806
1077	AAOX01000104	no match	32962
1086	AAOX01000103	match CH56	30649
1102	AAOX01000102	no match	28014
1107	AAOX01000101	plasmid	24909
1124	AAOX01000100	plasmid	22021
1141	AAOX01000099	plasmid	19130
1161	AAOX01000098	no match	19032
1164	AAOX01000097	within CH56 but not matching	14891
1173	AAOX01000096	plasmid	13654
1178	AAOX01000095	plasmid	12667
1218	AAOX01000094	match CH56	12571
1225	AAOX01000093		12506
1231	AAOX01000092	plasmid	12335
1266	AAOX01000091	repetitive	12263
1299	AAOX01000090	plasmid	10215
1349	AAOX01000089		9092
1455	AAOX01000088		6789
1473	AAOX01000087	split match 800kb apart	6364
1491	AAOX01000086	match CH55	6288
1793	AAOX01000085		4029
1969	AAOX01000083		3765
2724	AAOX01000080		3070
2841	AAOX01000078		2841
3070	AAOX01000076		2724
3162	AAOX01000075	plasmid	1969
3765	AAOX01000073		1793
4029	AAOX01000072		1473
4281	AAOX01000070	no match	1455
6364	AAOX01000067	split match 400kb apart	1349
6789	AAOX01000066		1225
9092	AAOX01000065		1164
10215	AAOX01000063		
12263	AAOX01000060		
12335	AAOX01000059		
12506	AAOX01000058		
12571	AAOX01000057		
12667	AAOX01000055		
13654	AAOX01000053		
14891	AAOX01000051		
19032	AAOX01000045		
24909	AAOX01000040		
28014	AAOX01000039		
30649	AAOX01000038		
32962	AAOX01000035		
37188	AAOX01000033		
39271	AAOX01000031		
39783	AAOX01000030		
72665	AAOX01000023		
91393	AAOX01000097		
134330	AAOX01000097		
142379	AAOX01000097		
146055	AAOX01000097		

Table S2: List of genes for virulence factor encoded in the genome of *Bacillus* sp. NRRL B-14911.

NRRLB14911 GI ID	NRRL B-14911 protein ID	NRRL B-14911 protein description	Length (aa)	Start position (genome)	End position (genome)	Strand (genome)	VFDB protein ID	VFDB protein description	VF length (aa)	% positive	Aligned length	E-value	Bit score
549474315	YP_008607974.1	ribonuclease yfkH	286	1149702	1150562	+	VFG0028	serum resistance protein [Bordetella pertussis Tohama I]	296	25.85	294	6.00E-15	70.1
549475378	YP_008609047.1	glycosyl transferase family 1	331	2215049	2216044	+	VFG0031	putative glycosyl transferase [Bordetella pertussis Tohama I]	390	29.2	137	3.00E-11	60.1
549476369	YP_008610050.1	N-acetyl-alpha-D-glucosaminyl L-malate synthase	378	3096145	3097281	-	VFG0031	putative glycosyl transferase [Bordetella pertussis Tohama I]	390	23.29	322	1.00E-12	65.1
549475286	YP_008608954.1	aminotransferase DegT	373	2116523	2117644	-	VFG0033	lipopolysaccharide biosynthesis protein [Bordetella pertussis Tohama I]	410	24.75	396	3.00E-24	99.4
549477593	YP_008611289.1	aminotransferase DegT	376	4357186	4358316	-	VFG0036	lipopolysaccharide biosynthesis protein [Bordetella pertussis Tohama I]	366	41.21	381	4.00E-90	276
549474760	YP_008608421.1	2,3,4,5-tetrahydropyridine-2,6-carboxylate N-succinyltransferase	236	1587357	1588067	+	VFG0037	probable acetyltransferase [Bordetella pertussis Tohama I]	191	30.52	154	6.00E-14	64.3
549477594	YP_008611290.1	acetyltransferase	187	4358334	4358897	-	VFG0037	probable acetyltransferase [Bordetella pertussis Tohama I]	191	52.46	183	5.00E-61	188
549474218	YP_008607876.1	NADH-dependent dehydrogenase	342	1047655	1048683	+	VFG0038	probable oxidoreductase [Bordetella pertussis Tohama I]	350	26.36	349	4.00E-28	109
549474219	YP_008607877.1	NADH-dependent dehydrogenase	346	1048695	1049735	+	VFG0038	probable oxidoreductase [Bordetella pertussis Tohama I]	350	26.7	367	1.00E-25	102
549475233	YP_008608901.1	hypothetical protein	315	2063798	2064745	+	VFG0038	probable oxidoreductase [Bordetella pertussis Tohama I]	350	23.29	249	2.00E-16	75.5
549475997	YP_008609671.1	NADH-dependent dehydrogenase	347	2741274	2742317	-	VFG0038	probable oxidoreductase [Bordetella pertussis Tohama I]	350	28.2	344	9.00E-33	123
549477071	YP_008610765.1	oxidoreductase	328	3791865	3792851	-	VFG0038	probable oxidoreductase [Bordetella pertussis Tohama I]	350	26.53	245	8.00E-15	70.9
549477592	YP_008611288.1	oxidoreductase	337	4356140	4357153	-	VFG0038	probable oxidoreductase [Bordetella pertussis Tohama I]	350	36.67	330	2.00E-76	239
549474625	YP_008608284.1	peptidase	309	1448499	1449428	+	VFG0070	P60 extracellular protein, invasion associated protein Iap [Listeria monocytogenes (serovar 1/2a) EGD-e]	482	35.79	95	4.00E-17	78.2
549475202	YP_008608870.1	hypothetical protein	329	2034061	2035050	+	VFG0070	P60 extracellular protein, invasion associated protein Iap [Listeria monocytogenes (serovar 1/2a) EGD-e]	482	42.2	109	2.00E-22	94
549475209	YP_008608877.1	peptidase P60	148	2041501	2041947	+	VFG0070	P60 extracellular protein, invasion associated protein Iap [Listeria monocytogenes (serovar 1/2a) EGD-e]	482	41.59	113	6.00E-22	88.6
549475444	YP_008609113.1	hypothetical protein	281	2279622	2280467	-	VFG0070	P60 extracellular protein, invasion associated protein Iap [Listeria monocytogenes (serovar 1/2a) EGD-e]	482	39.25	107	2.00E-12	63.5
549476049	YP_008609723.1	peptidase	433	2784510	2785811	-	VFG0070	P60 extracellular protein, invasion associated protein Iap [Listeria monocytogenes (serovar 1/2a) EGD-e]	482	38.94	113	1.00E-16	78.2
549477624	YP_008611320.1	hypothetical protein	333	4409260	4410261	-	VFG0070	P60 extracellular protein, invasion associated protein Iap [Listeria monocytogenes (serovar 1/2a) EGD-e]	482	46.15	117	2.00E-25	102
549477693	YP_008611391.1	hypothetical protein	171	4487703	4488218	+	VFG0070	P60 extracellular protein, invasion associated protein Iap [Listeria monocytogenes (serovar 1/2a) EGD-e]	482	38.79	116	3.00E-16	72.8
549477271	YP_008610965.1	ATP-dependent Clp protease proteolytic subunit	201	3971231	3971836	+	VFG0077	ATP-dependent Clp protease proteolytic subunit [Listeria monocytogenes (serovar 1/2a) EGD-e]	198	77.89	190	6.00E-111	316
549477775	YP_008611474.1	ATP-dependent Clp protease proteolytic subunit	193	4564393	4564974	-	VFG0077	ATP-dependent Clp protease proteolytic subunit [Listeria monocytogenes (serovar 1/2a) EGD-e]	198	69.27	192	8.00E-98	283
549473237	YP_008606860.1	ATP-dependent Clp protease ATP-binding protein	817	109202	111655	+	VFG0079	endopeptidase Clp ATP-binding chain C [Listeria monocytogenes (serovar 1/2a) EGD-e]	820	79.23	809	0	1313
549474534	YP_008608193.1	ATP-dependent Clp protease ATP-binding protein	866	1361133	1363733	+	VFG0079	endopeptidase Clp ATP-binding chain C [Listeria monocytogenes (serovar 1/2a) EGD-e]	820	46.31	868	0	733
549474716	YP_008608376.1	ATP-dependent Clp protease ATP-binding protein	708	1550832	1552958	-	VFG0080	ATP-dependent protease [Listeria monocytogenes (serovar 1/2a) EGD-e]	724	65.41	714	0	928
549473420	YP_008607067.1	1-pyrroline-5-carboxylate dehydrogenase	515	299210	300757	+	VFG0082	aldehyde dehydrogenase [Vibrio cholerae N16961]	541	31.57	472	1.00E-59	204
549473537	YP_008607184.1	aldehyde dehydrogenase	492	410856	412334	+	VFG0082	aldehyde dehydrogenase [Vibrio cholerae N16961]	541	28.9	481	1.00E-62	212
549473638	YP_008607286.1	hypothetical protein	465	498956	500353	+	VFG0082	aldehyde dehydrogenase [Vibrio cholerae N16961]	541	27.64	416	5.00E-34	130
549473913	YP_008607567.1	aldehyde dehydrogenase	431	759832	761127	+	VFG0082	aldehyde dehydrogenase [Vibrio cholerae N16961]	541	30.05	366	1.00E-38	144
549475396	YP_008609065.1	methylmalonate-semialdehyde dehydrogenase	487	2230861	2232324	-	VFG0082	aldehyde dehydrogenase [Vibrio cholerae N16961]	541	29.47	475	2.00E-54	189
549475405	YP_008609074.1	aldehyde dehydrogenase	466	2241994	2243394	+	VFG0082	aldehyde dehydrogenase [Vibrio cholerae N16961]	541	26.91	457	2.00E-40	149
549475461	YP_008609130.1	betaine-aldehyde dehydrogenase	494	2294836	2296320	+	VFG0082	aldehyde dehydrogenase [Vibrio cholerae N16961]	541	44.79	480	2.00E-141	418
549475779	YP_008609451.1	methylmalonate-semialdehyde dehydrogenase	485	2560359	2561816	-	VFG0082	aldehyde dehydrogenase [Vibrio cholerae N16961]	541	30.21	470	1.00E-56	196
549475990	YP_008609664.1	aldehyde dehydrogenase	488	2733524	2734990	-	VFG0082	aldehyde dehydrogenase [Vibrio cholerae N16961]	541	27.37	486	2.00E-51	181
549476228	YP_008609907.1	aldehyde dehydrogenase	473	2964119	2965540	-	VFG0082	aldehyde dehydrogenase [Vibrio cholerae N16961]	541	33.33	474	5.00E-73	239
549477525	YP_008611221.1	aldehyde dehydrogenase	498	4271931	4273427	-	VFG0082	aldehyde dehydrogenase [Vibrio cholerae N16961]	541	34.45	476	6.00E-80	259
549476928	YP_008610618.1	lipid hydroperoxide peroxidase	166	3636104	3636604	-	VFG0087	tagD protein [Vibrio cholerae N16961]	164	36.94	157	3.00E-31	110
549473998	YP_008607652.1	hypothetical protein	402	831171	832379	-	VFG0088	toxin co-regulated pilus biosynthesis protein I [Vibrio cholerae N16961]	620	33.96	159	4.00E-15	73.6
549475423	YP_008609092.1	hypothetical protein	434	2260794	2262098	-	VFG0088	toxin co-regulated pilus biosynthesis protein I [Vibrio cholerae N16961]	620	40.98	122	5.00E-20	89
549476552	YP_008610237.1	hypothetical protein	493	3261535	3263016	+	VFG0088	toxin co-regulated pilus biosynthesis protein I [Vibrio cholerae N16961]	620	30.34	267	2.00E-18	84.7
549477403	YP_008611098.1	hypothetical protein	426	4125264	4126544	-	VFG0088	toxin co-regulated pilus biosynthesis protein I [Vibrio cholerae N16961]	620	28.17	355	2.00E-25	105
549475146	YP_008608813.1	chemotaxis protein	561	1973569	1975254	+	VFG0104	accessory colonization factor AcfB [Vibrio cholerae N16961]	626	32.51	323	2.00E-35	137
549475227	YP_008608895.1	methyl-accepting chemotaxis protein	311	2057618	2058553	+	VFG0104	accessory colonization factor AcfB [Vibrio cholerae N16961]	626	36.63	172	4.00E-18	81.3
549476235	YP_008609914.1	hypothetical protein	572	2970727	2972445	+	VFG0104	accessory colonization factor AcfB [Vibrio cholerae N16961]	626	30.88	272	3.00E-26	109
549476631	YP_008610316.1	methyl-accepting chemotaxis protein	586	3328129	3329889	-	VFG0104	accessory colonization factor AcfB [Vibrio cholerae N16961]	626	28.57	252	2.00E-22	97.8
549476797	YP_008610486.1	type II secretion system protein F	401	3487491	3488696	-	VFG0113	still frameshift type 4 fimbrial biogenesis protein PilC [Pseudomonas aeruginosa PAO1]	374	38.67	375	1.00E-85	265
549474310	YP_008607969.1	LuxR family transcriptional regulator	233	1142656	1143357	-	VFG0119	alginate biosynthesis regulatory protein AlgR [Pseudomonas aeruginosa PAO1]	248	24.03	233	1.00E-19	81.6
549475529	YP_008609199.1	hypothetical protein	160	2353935	2354417	-	VFG0119	alginate biosynthesis regulatory protein AlgR [Pseudomonas aeruginosa PAO1]	248	38.26	115	2.00E-21	85.1
549477506	YP_008611202.1	hypothetical protein	511	4253046	4254581	-	VFG0119	alginate biosynthesis regulatory protein AlgR [Pseudomonas aeruginosa PAO1]	248	38.02	121	6.00E-15	71.2
549477553	YP_008611249.1	hypothetical protein	520	4309007	4310569	-	VFG0119	alginate biosynthesis regulatory protein AlgR [Pseudomonas aeruginosa PAO1]	248	34.75	118	8.00E-17	77
549478088	YP_008611792.1	LytTR family transcriptional regulator	242	4840358	4841086	-	VFG0119	alginate biosynthesis regulatory protein AlgR [Pseudomonas aeruginosa PAO1]	248	25.6	250	3.00E-19	80.9
549473501	YP_008607148.1	membrane protein	581	373576	375321	+	VFG0120	sigma factor AlgU [Pseudomonas aeruginosa PAO1]	358	26.01	296	7.00E-22	94
549475530	YP_008609200.1	histidine kinase	578	2354419	2356155	-	VFG0120	sigma factor AlgU [Pseudomonas aeruginosa PAO1]	358	36.79	193	1.00E-27	111
549477507	YP_008611203.1	hypothetical protein	586	4254583	4256343	-	VFG0120	sigma factor AlgU [Pseudomonas aeruginosa PAO1]	358	26.76	213	3.00E-12	64.7
549477554	YP_008611250.1	hypothetical protein	588	4310562	4312328	-	VFG0120	sigma factor AlgU [Pseudomonas aeruginosa PAO1]	358	26.02	196	3.00E-22	95.1
549477812	YP_008611511.1	hypothetical protein	489	4602378	4603847	-	VFG0120	sigma factor AlgU [Pseudomonas aeruginosa PAO1]	358	28.37	215	3.00E-21	91.7
549478089	YP_008611793.1	histidine kinase	555	4841083	4842750	-	VFG0120	sigma factor AlgU [Pseudomonas aeruginosa PAO1]	358	40.18	112	7.00E-21	90.9
549473374	YP_008607016.1	RNA polymerase sigma70 factor	183	245380	245931	-	VFG0121	alginate biosynthesis protein AlgZ/FimS [Pseudomonas aeruginosa PAO1]	193	24.6	187	2.00E-11	56.6
549473447	YP_008607094.1	DNA-directed RNA polymerase subunit sigma	191	323358	323933	+	VFG0121	alginate biosynthesis protein AlgZ/FimS [Pseudomonas aeruginosa PAO1]	193	32.95	173	2.00E-20	82.4
549473477	YP_008607124.1	RNA polymerase sigma factor	177	349058	349591	+	VFG0121	alginate biosynthesis protein AlgZ/FimS [Pseudomonas aeruginosa PAO1]	193	32.04	181	2.00E-23	90.5
549473481	YP_008607128.1	RNA polymerase sigma70	173	353117	353638	+	VFG0121	alginate biosynthesis protein AlgZ/FimS [Pseudomonas aeruginosa PAO1]	193	20.22	178	7.00E-14	63.2
549473514	YP_008607161.1	RNA polymerase sigma-70 factor	188	391658	392224	+	VFG0121	alginate biosynthesis protein AlgZ/FimS [Pseudomonas aeruginosa PAO1]	193	25.28	178	2.00E-14	65.5
549473657	YP_008607306.1	hypothetical protein	169	514750	515259	+	VFG0121	alginate biosynthesis protein AlgZ/FimS [Pseudomonas aeruginosa PAO1]	193	27.59	174	4.00E-18	75.5
549474111	YP_008607767.1	RNA polymerase sigma-70 factor	191	941638	942213	+	VFG0121	alginate biosynthesis protein AlgZ/FimS [Pseudomonas aeruginosa PAO1]	193	26.35	167	3.00E-17	73.2
549474656	YP_008608315.1	RNA polymerase sigma-70 factor	175	1480962	1481489	+	VFG0121	alginate biosynthesis protein AlgZ/FimS [Pseudomonas aeruginosa PAO1]	193	28.57	168	3.00E-17	73.2
549475594	YP_008609266.1	RNA polymerase sigma70 factor	186	2409362	2409922	-	VFG0121	alginate biosynthesis protein AlgZ/FimS [Pseudomonas aeruginosa PAO1]	193	27.01	174	1.00E-16	71.6
549475673	YP_008609345.1	hypothetical protein	184	2480298	2480852	-	VFG0121	alginate biosynthesis protein AlgZ/FimS [Pseudomonas aeruginosa PAO1]	193	27.92	154	6.00E-15	66.6
549475808	YP_008609482.1	RNA polymerase subunit sigma	186	2583479	2584039	+	VFG0121	alginate biosynthesis protein AlgZ/FimS [Pseudomonas aeruginosa PAO1]	193	29.59	169	2.00E-18	76.6
549475939	YP_008609613.1	RNA polymerase sigma-70 factor	188	2696749	2697315	-	VFG0121	alginate biosynthesis protein AlgZ/FimS [Pseudomonas aeruginosa PAO1]	193	30.34	178	1.00E-27	102
549477619	YP_008611315.1	RNA polymerase subunit sigma	183	4395254	4395805	-	VFG0121	alginate biosynthesis protein AlgZ/FimS [Pseudomonas aeruginosa PAO1]	193	31.61	155	3.00E-21	84.7
549475738	YP_008609410.1	nucleotide sugar dehydrogenase	435	2527240	2528547	-	VFG0122	GDP-mannose 6-dehydrogenase AlgD [Pseudomonas aeruginosa PAO1]	436	31.64	414	3.00E-60	202
549477787	YP_008611486.1	UDP-glucose 6-dehydrogenase	438	4574999	4576315	-	VFG0122	GDP-mannose 6-dehydrogenase AlgD [Pseudomonas aeruginosa PAO1]	436	33.33	357	9.00E-56	190
549477375	YP_008611070.1	acetyltransferase	481	4077756	4079201	-	VFG0130	alginate o-acetyltransferase AlgI [Pseudomonas aeruginosa PAO1]	520	37.69	398	1.00E-76	249
549478111	YP_008611817.1	glycosyl transferase	383	4867768	4868919	-	VFG0139	B-band O-antigen polymerase [Pseudomonas aeruginosa PAO1]	373	28.8	250	4.00E-17	78.6
549477814	YP_008611513.1	AraC family transcriptional regulator	298	4605066	4605962	+	VFG0167	transcriptional regulator PchR [Pseudomonas aeruginosa PAO1]	296	32.73	110	9.00E-12	61.2
549473798	YP_008607449.1	hypothetical protein	86	644094	644354	+	VFG0168	pyochelin biosynthesis protein PchD [Pseudomonas aeruginosa PAO1]	547	41.67	72	1.00E-11	57
549473799	YP_008607450.1	O-succinylbenzoate-CoA ligase	412	644373	645611	+	VFG0168	pyochelin biosynthesis protein PchD [Pseudomonas aeruginosa PAO1]	547	30.06	346	2.00E-36	137
549474374	YP_008608033.1	long-chain fatty acid--CoA ligase	538	1207061	1208677	+	VFG0168	pyochelin biosynthesis protein PchD [Pseudomonas aeruginosa PAO1]	547	27.09	502	6.00E-35	134
549474437	YP_008608096.1	long-chain fatty acid--CoA ligase	519	1261518	1263077	+	VFG0168	pyochelin biosynthesis protein PchD [Pseudomonas aeruginosa PAO1]	547	29.48	502	3.00E-55	193
549474592	YP_008608251.1	long-chain fatty acid--CoA ligase	538	1415992	1417608	+	VFG0168	pyochelin biosynthesis protein PchD [Pseudomonas aeruginosa PAO1]	547	26.1	521	2.00E-41	153
549475487	YP_008609156.1	AMP-binding protein	554	2317003	2318667	-	VFG0168	pyochelin biosynthesis protein PchD [Pseudomonas aeruginosa PAO1]	547	25.14	529	5.00E-42	155
549476859	YP_008610548.1	long-chain fatty acid--CoA ligase	567	3561075	3562778	-	VFG0168	pyochelin biosynthesis protein PchD [Pseudomonas aeruginosa PAO1]	547	26.54	535	8.00E-44	161
549476936	YP_008610626.1	acyl--CoA ligase	528	3643583	3645169	-	VFG0168	pyochelin biosynthesis protein PchD [Pseudomonas aeruginosa PAO1]	547	25.05	519	8.00E-38	142
549477051	YP_008610745.1	O-succinylbenzoic acid--CoA ligase	495	3761656	3763143	-	VFG0168	pyochelin biosynthesis protein PchD [Pseudomonas aeruginosa PAO1]	547	26.72	494	1.00E-27	112
549473224	YP_008606847.1	para-aminobenzoate synthase	471	87062	88477	+	VFG0171	salicylate biosynthesis isochorismate synthase [Pseudomonas aeruginosa PAO1]	476	29.33	283	5.00E-34	130
549477055	YP_008610749.1	isochorismate synthase	468	3766629	3768035	-	VFG0171	salicylate biosynthesis isochorismate synthase [Pseudomonas aeruginosa PAO1]	476	35.19	466	5.00E-83	264
549476799	YP_008610488.1	type II secretion system protein E	553	3489749	3491410	-	VFG0182	general secretion pathway protein E [Pseudomonas aeruginosa PAO1]	502	51.04	384	5.00E-132	395
549477678	YP_008611375.1	F0F1 ATP synthase subunit alpha	502	4472276	4473784	-	VFG0192	ATP synthase in type III secretion system [Pseudomonas aeruginosa PAO1]	440	29.79	386	4.00E-43	156
549476798	YP_008610487.1	twitching motility protein PilT	346	3488698	3489738	-	VFG0232	twitching motility protein PilT [Neisseria meningitidis MC58 (serogroup B)]	347	46.67	330	4.00E-108	320
549477616	YP_008611312.1	glycosyl transferase family 1	390	4390660	4391832	+	VFG0242	alpha-1,2-N-acetylglucosamine transferase [Neisseria meningitidis MC58 (serogroup B)]	354	30.07	143	2.00E-12	64.3
549473331	YP_008606973.1	general stress protein	143	204953	205384	-	VFG0307	neutrophil activating protein (napA) (bacterioferritin) [Helicobacter pylori 26695]	144	37.59	133	1.00E-24	91.7
549477039	YP_008610733.1	general stress protein	145	3751262	3751699	-	VFG0307	neutrophil activating protein (napA) (bacterioferritin) [Helicobacter pylori 26695]	144	34.59	133	6.00E-22	84.3
549475283	YP_008608951.1	spore coat protein	318	2114001	2114957	-	VFG0314	UDP-glucose 4-epimerase [Helicobacter pylori 26695]	344	25.36	351	1.00E-21	90.1
549474832	YP_008608494.1	phosphopantetheine adenylyltransferase	162	1655265	1655753	+	VFG0320	lipopolysaccharide core biosynthesis protein (kdtB) [Helicobacter pylori 26695]	157	45.16	155	4.00E-44	143
549473097	YP_008607709.1	ABC transporter	242	881061	881789	-	VFG0344	iron(III) ABC transporter, ATP-binding protein [Haemophilus influenzae Rd]	351	34.73	239	8.00E-44	150
549473652	YP_008607301.1	hypothetical protein	289	511264	512133	+	VFG0344	iron(III) ABC transporter, ATP-binding protein [Haemophilus influenzae Rd]	351	25.57	219	9.00E-19	81.6
549473663	YP_008607312.1	ABC transporter ATP-binding protein	301	518293	519198	+	VFG0344	iron(III) ABC transporter, ATP-binding protein [Haemophilus influenzae Rd]	351	28.5	214	2.00E-22	92
549474053	YP_008607708.1	elongation factor 3	543	879237	880868	+	VFG0344	iron(III) ABC transporter, ATP-binding protein [Haemophilus influenzae Rd]	351	25.89	197	2.00E-11	62
549474158	YP_008607815.1	ABC transporter ATP-binding protein	359	984819	985898	+	VFG0344	iron(III) ABC transporter, ATP-binding protein [Haemophilus influenzae Rd]	351	38.52	244	1.00E-57	191
549474191	YP_008607849.1	peptide ABC transporter ATP-binding protein	336	1011004	1012014	+	VFG0344	iron(III) ABC transporter, ATP-binding protein [Haemophilus influenzae Rd]	351	28.29	251	3.00E-26	103
549474192	YP_008607850.1	peptide ABC transporter substrate-binding protein	328	1011995	1012981	+	VFG0344	iron(III) ABC transporter, ATP-binding protein [Haemophilus influenzae Rd]	351	29.5	278	1.00E-34	128
549474403	YP_008608062.1	ABC transporter ATP-binding protein	252	1231215	1231973	+	VFG0344	iron(III) ABC transporter, ATP-binding protein [Haemophilus influenzae Rd]	351	29.02	224	3.00E-24	96.7
549474460	YP_008608119.1	branched-chain amino acid ABC transporter ATP-binding protein	235	1284106	1284813	+	VFG0344	iron(III) ABC transporter, ATP-binding protein [Haemophilus influenzae Rd]	351	25.79	221	1.00E-17	77
549474545	YP_008608204.1	peptide ABC transporter substrate-binding protein	337	1372356	1373369	+	VFG0344	iron(III) ABC transporter, ATP-binding protein [Haemophilus influenzae Rd]	351	29.84	248	8.00E-29	111
549474555	YP_008608214.1	peptide ABC transporter ATP-binding protein	355	1384171	1385238	+	VFG0344	iron(III) ABC transporter, ATP-binding protein [Haemophilus influenzae Rd]	351	28.16	277	3.00E-27	107
549474556	YP_008608215.1	peptide ABC transporter substrate-binding protein	311	1385240	1386175	+	VFG0344	iron(III) ABC transporter, ATP-binding protein [Haemophilus influenzae Rd]	351	33.2	256	2.00E-35	130
549474621	YP_008608280.1	peptide ABC transporter ATP-binding protein	333	1443660	1444661	+	VFG0344	iron(III) ABC transporter, ATP-binding protein [Haemophilus influenzae Rd]	351	28.06	253	7.00E-27	105
549476117	YP_008609792.1	sugar ABC transporter ATPase	262	2850296	2851084	-	VFG0344	iron(III) ABC transporter, ATP-binding protein [Haemophilus influenzae Rd]	351	36.8	231	5.00E-45	154
549476278	YP_008609958.1	multidrug ABC transporter ATP-binding protein	626	3011281	3013161	-	VFG0344	iron(III) ABC transporter, ATP-binding protein [Haemophilus influenzae Rd]	351	28.44	218	1.00E-19	87
549476620	YP_008610305.1	phosphate ABC transporter ATP-binding protein	275	3316201	3317028	-	VFG0344	iron(III) ABC transporter, ATP-binding protein [Haemophilus influenzae Rd]	351	30.04	243	2.00E-28	108
549477176	YP_008610870.1	methionine ABC transporter ATP-binding protein	341	3879188	3880213	-	VFG0344	iron(III) ABC transporter, ATP-binding protein [Haemophilus influenzae Rd]	351	26.75	329	3.00E-32	121
549477767	YP_008611466.1	ABC transporter ATP-binding protein	243	4556070	4556801	+	VFG0344	iron(III) ABC transporter, ATP-binding protein [Haemophilus influenzae Rd]	351	34.31	239	1.00E-37	134
549477832	YP_008611531.1	bacitracin ABC transporter ATP-binding protein	253	4623920	4624681	+	VFG0344	iron(III) ABC transporter, ATP-binding protein [Haemophilus influenzae Rd]	351	31.6	212	2.00E-30	114
549477960	YP_008611662.1	glycosyl transferase family 8	257	4726215	4726988	-	VFG0344	iron(III) ABC transporter, ATP-binding protein [Haemophilus influenzae Rd]	351	27.27	231	2.00E-22	90.9
549478001	YP_008611703.1	ABC transporter ATP-binding protein	308	4750819	4751745	-	VFG0344	iron(III) ABC transporter, ATP-binding protein [Haemophilus influenzae Rd]	351	27.15	221	3.00E-23	94.7
549474469	YP_008608128.1	long-chain fatty acid--CoA ligase	502	1292093	1293601	+	VFG0359	yersiniabactin siderophore biosynthetic protein [Yersinia pestis CO92]	525	28.48	495	9.00E-45	162
549475762	YP_008609434.1	ABC transporter	609	2546361	2548190	-	VFG0366	inner membrane ABC-transporter YbtQ [Yersinia pestis CO92]	600	31.14	533	4.00E-71	240
549476124	YP_008609799.1	anthranilate synthase subunit I	467	2856282	2857685	-	VFG0368	putative salicylate synthetase [Yersinia pestis CO92]	434	33.73	255	2.00E-33	127
549477676	YP_008611373.1	F0F1 ATP synthase subunit beta	473	4469664	4471085	-	VFG0390	putative Yops secretion ATP synthase [Yersinia pestis CO92]	439	29.53	430	2.00E-44	160
549477708	YP_008611406.1	transcription termination factor Rho	424	4500054	4501328	-	VFG0390	putative Yops secretion ATP synthase [Yersinia pestis CO92]	439	31.72	268	7.00E-19	85.1
549475335	YP_008609004.1	UDP-glucose 4-epimerase	316	2173673	2174623	+	VFG0430	Vi polysaccharide biosynthesis protein, epimerase [Salmonella enterica (serovar typhi) CT18]	348	27.63	257	7.00E-12	62
549475737	YP_008609409.1	NAD-dependent epimerase	335	2526207	2527214	-	VFG0430	Vi polysaccharide biosynthesis protein, epimerase [Salmonella enterica (serovar typhi) CT18]	348	30.96	323	6.00E-42	148
549478097	YP_008611801.1	sensor histidine kinase	609	4848572	4850401	-	VFG0472	sensory kinase in two-component regulatory system with BasR [Salmonella enterica (serovar typhimurium) LT2]	356	29.01	262	3.00E-23	98.2
549475903	YP_008609577.1	transcriptional regulator	232	2663034	2663732	-	VFG0475	response regulator in two-component regulatory system with PhoQ, transcribes genes expressed under low Mg+ co	224	28.82	229	2.00E-30	111
549474870	YP_008608532.1	RNA polymerase sigma 70	239	1691736	1692455	+	VFG0477	sigma S (sigma 38) factor of RNA polymerase, major sigmafactor during stationary phase [Salmonella enterica (serov	330	28.91	256	1.00E-21	88.6
549476705	YP_008610393.1	hypothetical protein	240	3400627	3401349	-	VFG0477	sigma S (sigma 38) factor of RNA polymerase, major sigmafactor during stationary phase [Salmonella enterica (serov	330	26.12	245	1.00E-16	74.3
549474208	YP_008607866.1	Fur family transcriptional regulator	145	1031667	1032104	+	VFG0478	transcriptional repressor of iron-responsive genes (Fur family) (ferric uptake regulator) [Salmonella enterica (serova	150	30.88	136	4.00E-17	71.6
549476479	YP_008610162.1	Fur family transcriptional regulator	154	3191740	3192204	-	VFG0478	transcriptional repressor of iron-responsive genes (Fur family) (ferric uptake regulator) [Salmonella enterica (serova	150	35.42	144	8.00E-22	84.3
549476636	YP_008610321.1	Fur family transcriptional regulator	137	3332949	3333362	-	VFG0478	transcriptional repressor of iron-responsive genes (Fur family) (ferric uptake regulator) [Salmonella enterica (serova	150	31.78	129	6.00E-15	65.1
549476901	YP_008610591.1	pyruvate kinase	586	3607602	3609362	-	VFG0479	pyruvate kinase I (formerly F), fructose stimulated [Salmonella enterica (serovar typhimurium) LT2]	470	52.42	475	3.00E-165	480
549475931	YP_008609605.1	hypothetical protein	277	2690664	2691497	-	VFG0481	putative hydrolase or acyltransferase [Salmonella enterica (serovar typhimurium) LT2]	297	30.77	104	3.00E-11	59.3
549477413	YP_008611108.1	fructokinase	318	4137103	4138059	-	VFG0483	putative regulatory protein, deoR family [Salmonella enterica (serovar typhimurium) LT2]	408	25.19	258	2.00E-11	60.8
549476421	YP_008610104.1	MerR family transcriptional regulator	301	3143725	3144630	-	VFG0491	putative regulatory proteins, merR family [Salmonella enterica (serovar typhimurium) LT2]	242	36.99	73	1.00E-12	62.8
549474257	YP_008607915.1	hypothetical protein	457	1091936	1093309	+	VFG0493	Secretion system regulator:Sensor component [Salmonella enterica (serovar typhimurium) LT2]	920	27.42	423	1.00E-31	125

549475617	YP_008609289.1	hypothetical protein	1722	2428909	2434077	-	VFG0493	Secretion system regulator:Sensor component [Salmonella enterica (serovar typhimurium) LT2]	920	33.08	266	2.00E-26	114
549476533	YP_008610218.1	ATPase AAA	692	3243235	3245313	-	VFG0524	formate hydrogen-lyase transcriptional activator for fdhF, hyc and hyp operons (EBP family) [Salmonella enterica (se	692	40.91	330	2.00E-69	239
549477002	YP_008610695.1	ATPase AAA	454	3719257	3720621	-	VFG0524	formate hydrogen-lyase transcriptional activator for fdhF, hyc and hyp operons (EBP family) [Salmonella enterica (se	692	44.83	319	2.00E-80	263
549476295	YP_008609975.1	manganese ABC transporter ATP-binding protein	249	3030488	3031237	-	VFG0526	Salmonella iron transporter: fur regulated [Salmonella enterica (serovar typhimurium) LT2]	273	44.12	238	2.00E-70	217
549477238	YP_008610932.1	ABC transporter ATP-binding protein	313	3934923	3935864	-	VFG0526	Salmonella iron transporter: fur regulated [Salmonella enterica (serovar typhimurium) LT2]	273	28.93	242	2.00E-28	108
549476293	YP_008609973.1	iron ABC transporter	293	3028286	3029167	-	VFG0527	Salmonella iron transporter: fur regulated [Salmonella enterica (serovar typhimurium) LT2]	286	28.57	287	6.00E-34	124
549476294	YP_008609974.1	manganese ABC transporter permease	437	3029164	3030477	-	VFG0527	Salmonella iron transporter: fur regulated [Salmonella enterica (serovar typhimurium) LT2]	286	33.77	302	2.00E-34	127
549476637	YP_008610322.1	metal ABC transporter permease	287	3333419	3334282	-	VFG0528	Salmonella iron transporter: fur regulated [Salmonella enterica (serovar typhimurium) LT2]	282	29.12	261	3.00E-27	105
549475055	YP_008608720.1	DNA mismatch repair protein MutS	883	1882508	1885159	+	VFG0562	methyl-directed mismatch repair, recognize exocyclic adducts of guanosine [Salmonella enterica (serovar typhimuri	855	38.89	882	0	610
549475460	YP_008609129.1	DNA mismatch repair protein	642	2292373	2294301	-	VFG0562	methyl-directed mismatch repair, recognize exocyclic adducts of guanosine [Salmonella enterica (serovar typhimuri	855	27.33	322	1.00E-22	99.4
549476861	YP_008610550.1	DNA mismatch repair protein MutS	784	3563497	3565851	-	VFG0562	methyl-directed mismatch repair, recognize exocyclic adducts of guanosine [Salmonella enterica (serovar typhimuri	855	26.22	347	8.00E-22	97.4
549473873	YP_008607526.1	hypothetical protein	443	725814	727145	+	VFG0564	ATP binding protein [Salmonella enterica (serovar typhimurium) LT2]	396	41	100	6.00E-15	72.8
549473818	YP_008607469.1	ATPase	639	663854	665773	-	VFG0574	Mg<up>2+</up> transport protein [Salmonella enterica (serovar typhimurium) LT2]	908	28.57	189	7.00E-13	68.2
549474895	YP_008608557.1	ATPase	892	1717118	1719796	+	VFG0574	Mg<up>2+</up> transport protein [Salmonella enterica (serovar typhimurium) LT2]	908	27.71	877	2.00E-92	311
549476062	YP_008609736.1	ATPase P	804	2798885	2801299	-	VFG0574	Mg<up>2+</up> transport protein [Salmonella enterica (serovar typhimurium) LT2]	908	29	231	6.00E-20	91.7
549478014	YP_008611716.1	potassium-transporting ATPase subunit B	713	4762369	4764510	-	VFG0574	Mg<up>2+</up> transport protein [Salmonella enterica (serovar typhimurium) LT2]	908	23.64	643	3.00E-20	92
549477067	YP_008610761.1	methyltransferase	233	3788396	3789097	-	VFG0575	Mg<up>2+</up> transport protein [Salmonella enterica (serovar typhimurium) LT2]	231	38.21	123	3.00E-18	77.4
549473138	YP_008611336.1	single-stranded DNA-binding protein	121	4437988	4438353	-	VFG0576	ssDNA-binding protein controls activity of RecBCD nuclease [Salmonella enterica (serovar typhimurium) LT2]	176	36.08	97	4.00E-17	71.2
549473147	YP_008611815.1	single-stranded DNA-binding protein	173	4866735	4867256	-	VFG0576	ssDNA-binding protein controls activity of RecBCD nuclease [Salmonella enterica (serovar typhimurium) LT2]	176	38.14	97	2.00E-17	73.2
549473602	YP_008607250.1	histidine kinase	744	462483	464717	+	VFG0584	putative diguanylate cyclase/phosphodiesterase [Salmonella enterica (serovar typhimurium) LT2]	533	32.39	247	1.00E-36	142
549473804	YP_008607455.1	hypothetical protein	1102	650695	654003	-	VFG0584	putative diguanylate cyclase/phosphodiesterase [Salmonella enterica (serovar typhimurium) LT2]	533	33.46	257	3.00E-38	148
549473959	YP_008607613.1	hypothetical protein	242	805989	806717	+	VFG0584	putative diguanylate cyclase/phosphodiesterase [Salmonella enterica (serovar typhimurium) LT2]	533	22.32	224	3.00E-17	77.4
549474109	YP_008607765.1	hypothetical protein	681	939052	941097	+	VFG0584	putative diguanylate cyclase/phosphodiesterase [Salmonella enterica (serovar typhimurium) LT2]	533	32.3	257	5.00E-34	133
549474487	YP_008608146.1	diguanylate cyclase	974	1310746	1313670	+	VFG0584	putative diguanylate cyclase/phosphodiesterase [Salmonella enterica (serovar typhimurium) LT2]	533	34.14	249	5.00E-36	140
549474629	YP_008608288.1	hypothetical protein	673	1451755	1453776	+	VFG0584	putative diguanylate cyclase/phosphodiesterase [Salmonella enterica (serovar typhimurium) LT2]	533	30.71	241	1.00E-34	135
549474676	YP_008608335.1	signal peptide protein	307	1502946	1503869	+	VFG0584	putative diguanylate cyclase/phosphodiesterase [Salmonella enterica (serovar typhimurium) LT2]	533	25	232	8.00E-18	80.1
549476104	YP_008609778.1	hypothetical protein	793	2836070	2838451	+	VFG0584	putative diguanylate cyclase/phosphodiesterase [Salmonella enterica (serovar typhimurium) LT2]	533	35.56	239	3.00E-36	140
549477151	YP_008610845.1	diguanylate cyclase	434	3856740	3858044	-	VFG0584	putative diguanylate cyclase/phosphodiesterase [Salmonella enterica (serovar typhimurium) LT2]	533	23.93	234	2.00E-19	87.4
549473962	YP_008607616.1	AraC family transcriptional regulator	311	808252	809187	-	VFG0585	transcriptional activator of superoxide response regulon (AraC/XylS family) [Salmonella enterica (serovar typhimuriu	107	31	100	7.00E-12	58.9
549475151	YP_008608818.1	hypothetical protein	740	1980629	1982851	+	VFG0585	transcriptional activator of superoxide response regulon (AraC/XylS family) [Salmonella enterica (serovar typhimuriu	107	29.7	101	3.00E-11	58.9
549475913	YP_008609587.1	AraC family transcriptional regulator	283	2672047	2672898	-	VFG0585	transcriptional activator of superoxide response regulon (AraC/XylS family) [Salmonella enterica (serovar typhimuriu	107	35.85	106	3.00E-21	84.3
549473529	YP_008607176.1	regulator	224	403870	404544	+	VFG0596	Copper resistance; transcriptional regulatory protein [Salmonella enterica (serovar typhimurium) LT2]	248	38.84	224	9.00E-47	155
549478008	YP_008611710.1	heme transporter CcmC	223	4756324	4756995	-	VFG0596	Copper resistance; transcriptional regulatory protein [Salmonella enterica (serovar typhimurium) LT2]	248	35.29	221	2.00E-40	138
549473806	YP_008607457.1	lysine/ornithine N-monooxygenase	439	655088	656407	+	VFG0618	lysine 6-monooxygenase IucD [imported] [Shigella flexneri (serotype 2a) 301]	445	31.97	416	8.00E-72	233
549475001	YP_008608664.1	transposase	370	1821987	1823099	+	VFG0628	orf, partial conserved hypothetical protein [Shigella flexneri (serotype 2a) 301]	121	49.02	102	1.00E-21	86.7
549473574	YP_008607222.1	RNA-directed DNA polymerase	427	437743	439026	+	VFG0639	orf, conserved hypothetical protein [Shigella flexneri (serotype 2a) 301]	502	30.89	382	3.00E-48	171
549473099	YP_008607819.1	bactoprenol glucosyl transferase	323	988376	989347	+	VFG0670	bactoprenol glucosyl transferase [Shigella flexneri (serotype 2a) 301]	309	40.79	304	1.00E-78	243
549473112	YP_008609237.1	bactoprenol glucosyl transferase	345	2384114	2385151	-	VFG0670	bactoprenol glucosyl transferase [Shigella flexneri (serotype 2a) 301]	309	38.06	310	2.00E-64	207
549473837	YP_008607489.1	gamma-glutamyltranspeptidase	562	680721	682409	+	VFG0679	<gamma>-glutamyltranspeptidase [Bacillus anthracis]	466	30.65	522	2.00E-59	204
549475976	YP_008609650.1	gamma-glutamyltranspeptidase	591	2721765	2723540	-	VFG0679	<gamma>-glutamyltranspeptidase [Bacillus anthracis]	466	32.58	528	7.00E-70	233
549477656	YP_008611353.1	gamma-glutamyltransferase	535	4449581	4451188	-	VFG0679	<gamma>-glutamyltranspeptidase [Bacillus anthracis]	466	28.14	501	6.00E-40	148
549473241	YP_008606864.1	2-C-methyl-D-erythritol 4-phosphate cytidylyltransferase	230	115654	116346	+	VFG0700	bifunctional; ribulose 5-phosphate reductase; CDP-ribitol pyrophosphorylase [Haemophilus influenzae 1007]	474	29.79	235	8.00E-26	101
549474352	YP_008608011.1	multidrug ABC transporter permease	669	1185322	1187331	+	VFG0841	hemolysin transport protein [Escherichia coli O157:H7 EDL933]	706	31.96	510	1.00E-72	248
549474764	YP_008608425.1	multidrug ABC transporter ATP-binding protein	605	1592225	1594042	+	VFG0841	hemolysin transport protein [Escherichia coli O157:H7 EDL933]	706	35.03	491	4.00E-83	275
549475901	YP_008609575.1	bacitracin ABC transporter ATP-binding protein	256	2661175	2661945	-	VFG0869	AatC ATB binding protein of ABC transporter [Escherichia coli 042]	209	38.04	184	3.00E-32	116
549473316	YP_008606958.1	hypothetical protein	185	191229	191786	-	VFG0872	Type 1 fimbriae Regulatory protein fimE [Escherichia coli CFT073]	198	34	150	8.00E-17	72
549475149	YP_008608816.1	ABC transporter	587	1977850	1979613	-	VFG0907	Hemolysin B [Escherichia coli CFT073]	707	28.68	551	3.00E-68	234
549475307	YP_008608976.1	ABC transporter ATP-binding protein	264	2142584	2143378	+	VFG0907	Hemolysin B [Escherichia coli CFT073]	707	29.67	246	1.00E-23	97.4
549473341	YP_008606983.1	iron ABC transporter substrate-binding protein	323	215963	216934	+	VFG0918	Putative Periplasmic binding protein [Escherichia coli CFT073]	330	27.8	259	1.00E-25	101
549474372	YP_008608031.1	coproporphyrinogen III oxidase	501	1203717	1205222	+	VFG0919	Putative oxygen independent coproporphyrinogen III oxidase [Escherichia coli CFT073]	445	26.47	170	2.00E-13	68.6
549476679	YP_008610366.1	coproporphyrinogen III oxidase	379	3376858	3377997	-	VFG0919	Putative oxygen independent coproporphyrinogen III oxidase [Escherichia coli CFT073]	445	27.86	384	5.00E-41	148
549475584	YP_008609255.1	NAD dependent epimerase/dehydratase	214	2400032	2400676	+	VFG0921	Hypothetical protein [Escherichia coli CFT073]	207	28.44	211	2.00E-12	60.1
549473342	YP_008606984.1	corrinoid ABC transporter permease	353	216900	217961	+	VFG0922	Putative permease of iron compound ABC transport system [Escherichia coli CFT073]	330	41.97	305	1.00E-63	205
549473924	YP_008607578.1	iron ABC transporter permease	318	770107	771063	+	VFG0922	Putative permease of iron compound ABC transport system [Escherichia coli CFT073]	330	28.39	310	1.00E-27	107
549473925	YP_008607579.1	iron ABC transporter permease	318	771050	772006	+	VFG0922	Putative permease of iron compound ABC transport system [Escherichia coli CFT073]	330	24.73	283	2.00E-12	63.5
549474199	YP_008607857.1	daunorubicin ABC transporter ATP-binding protein	335	1025124	1026131	+	VFG0925	Ferric enterobactin transport ATP-binding protein fepC [Escherichia coli CFT073]	271	28.31	219	1.00E-21	89.7
549474459	YP_008608118.1	amino acid ABC transporter ATP-binding protein	255	1283355	1284122	+	VFG0925	Ferric enterobactin transport ATP-binding protein fepC [Escherichia coli CFT073]	271	28.45	239	2.00E-29	110
549475383	YP_008609052.1	phosphonate ABC transporter ATP-binding protein	250	2218946	2219698	+	VFG0925	Ferric enterobactin transport ATP-binding protein fepC [Escherichia coli CFT073]	271	31.56	225	9.00E-36	127
549477194	YP_008610888.1	iron-enterobactin transporter ATP-binding protein	275	3896219	3897046	-	VFG0925	Ferric enterobactin transport ATP-binding protein fepC [Escherichia coli CFT073]	271	50.59	253	2.00E-96	285
549477921	YP_008611623.1	sodium ABC transporter ATP-binding protein	288	4698748	4699614	-	VFG0925	Ferric enterobactin transport ATP-binding protein fepC [Escherichia coli CFT073]	271	23.44	209	7.00E-17	75.1
549478072	YP_008611776.1	iron-dicitrate ABC transporter ATP-binding protein	272	4819983	4820801	+	VFG0925	Ferric enterobactin transport ATP-binding protein fepC [Escherichia coli CFT073]	271	51.95	256	7.00E-99	291
549477196	YP_008610890.1	siderophore ABC transporter permease	333	3898153	3899154	-	VFG0926	Ferric enterobactin transport system permease protein fepD [Escherichia coli CFT073]	338	43.09	304	6.00E-64	206
549478070	YP_008611774.1	iron ABC transporter	344	4817921	4818955	+	VFG0926	Ferric enterobactin transport system permease protein fepD [Escherichia coli CFT073]	338	42.29	279	7.00E-54	180
549478071	YP_008611775.1	ABC transporter permease	343	4818952	4819983	+	VFG0928	Ferric enterobactin transport system permease protein fepG [Escherichia coli CFT073]	330	34.98	303	1.00E-43	152
549474481	YP_008608140.1	AMP-dependent synthetase	530	1305608	1307200	+	VFG0932	Enterobactin synthetase component E [Escherichia coli CFT073]	536	26.3	540	9.00E-36	136
549473166	YP_008606788.1	isochorismatase	187	27911	28474	-	VFG0933	Isochorismatase [Escherichia coli CFT073]	285	30.82	159	6.00E-14	65.1
549473644	YP_008607292.1	oxidoreductase	264	505065	505859	+	VFG0934	2,3-dihydro-2,3-dihydroxybenzoate dehydrogenase [Escherichia coli CFT073]	248	26.25	259	2.00E-15	70.1
549474092	YP_008607748.1	short-chain dehydrogenase	261	922632	923417	+	VFG0934	2,3-dihydro-2,3-dihydroxybenzoate dehydrogenase [Escherichia coli CFT073]	248	25.97	181	8.00E-11	57.4
549474466	YP_008608125.1	short-chain dehydrogenase	246	1288836	1289576	+	VFG0934	2,3-dihydro-2,3-dihydroxybenzoate dehydrogenase [Escherichia coli CFT073]	248	30.04	253	1.00E-30	112
549474509	YP_008608168.1	short-chain dehydrogenase	290	1338892	1339764	-	VFG0934	2,3-dihydro-2,3-dihydroxybenzoate dehydrogenase [Escherichia coli CFT073]	248	27.45	255	3.00E-22	90.1
549474591	YP_008608250.1	gluconate 2-dehydrogenase	257	1415117	1415890	+	VFG0934	2,3-dihydro-2,3-dihydroxybenzoate dehydrogenase [Escherichia coli CFT073]	248	27.52	258	1.00E-19	82
549474723	YP_008608383.1	3-ketoacyl-ACP reductase	246	1557222	1557962	+	VFG0934	2,3-dihydro-2,3-dihydroxybenzoate dehydrogenase [Escherichia coli CFT073]	248	27.84	255	4.00E-26	100
549474732	YP_008608392.1	3-oxoacyl-ACP reductase	260	1565308	1566090	+	VFG0934	2,3-dihydro-2,3-dihydroxybenzoate dehydrogenase [Escherichia coli CFT073]	248	27.95	254	1.00E-23	94
549474748	YP_008608408.1	short-chain dehydrogenase	253	1578461	1579222	+	VFG0934	2,3-dihydro-2,3-dihydroxybenzoate dehydrogenase [Escherichia coli CFT073]	248	27.52	258	1.00E-19	82
549474919	YP_008608581.1	3-ketoacyl-ACP reductase	247	1742700	1743443	+	VFG0934	2,3-dihydro-2,3-dihydroxybenzoate dehydrogenase [Escherichia coli CFT073]	248	31.78	258	1.00E-32	118
549475038	YP_008608702.1	3-ketoacyl-ACP reductase	248	1863417	1864163	+	VFG0934	2,3-dihydro-2,3-dihydroxybenzoate dehydrogenase [Escherichia coli CFT073]	248	26.77	254	1.00E-19	81.6
549475105	YP_008608771.1	dehydrogenase	424	1928888	1930162	+	VFG0934	2,3-dihydro-2,3-dihydroxybenzoate dehydrogenase [Escherichia coli CFT073]	248	25.97	258	4.00E-15	71.2
549475174	YP_008608841.1	2-deoxy-D-gluconate 3-dehydrogenase	260	2006941	2007723	+	VFG0934	2,3-dihydro-2,3-dihydroxybenzoate dehydrogenase [Escherichia coli CFT073]	248	28.4	250	6.00E-24	94.7
549475321	YP_008608990.1	3-hydroxy-2-methylbutyryl-CoA dehydrogenase	255	2157223	2157990	+	VFG0934	2,3-dihydro-2,3-dihydroxybenzoate dehydrogenase [Escherichia coli CFT073]	248	26.96	230	1.00E-14	68.2
549475605	YP_008609277.1	short-chain dehydrogenase	283	2417102	2417953	-	VFG0934	2,3-dihydro-2,3-dihydroxybenzoate dehydrogenase [Escherichia coli CFT073]	248	28.27	191	4.00E-14	67
549475677	YP_008609349.1	glucose-1-dehydrogenase	273	2484394	2485215	-	VFG0934	2,3-dihydro-2,3-dihydroxybenzoate dehydrogenase [Escherichia coli CFT073]	248	28.91	256	1.00E-24	97.4
549475723	YP_008609395.1	oxidoreductase	295	2517622	2518509	-	VFG0934	2,3-dihydro-2,3-dihydroxybenzoate dehydrogenase [Escherichia coli CFT073]	248	27.97	261	1.00E-19	82.8
549475788	YP_008609460.1	short-chain dehydrogenase	251	2570099	2570854	+	VFG0934	2,3-dihydro-2,3-dihydroxybenzoate dehydrogenase [Escherichia coli CFT073]	248	26.88	253	2.00E-21	87.4
549475952	YP_008609626.1	3-oxoacyl-ACP reductase	256	2703796	2704566	-	VFG0934	2,3-dihydro-2,3-dihydroxybenzoate dehydrogenase [Escherichia coli CFT073]	248	26.04	265	4.00E-17	74.7
549476317	YP_008609998.1	2-deoxy-D-gluconate 3-dehydrogenase	273	3048917	3049738	-	VFG0934	2,3-dihydro-2,3-dihydroxybenzoate dehydrogenase [Escherichia coli CFT073]	248	29.53	254	1.00E-20	85.1
549477000	YP_008610693.1	3-hydroxybutyrate dehydrogenase	258	3716776	3717552	-	VFG0934	2,3-dihydro-2,3-dihydroxybenzoate dehydrogenase [Escherichia coli CFT073]	248	26.85	257	3.00E-20	84
549477072	YP_008610766.1	3-ketoacyl-ACP reductase	262	3792990	3793778	+	VFG0934	2,3-dihydro-2,3-dihydroxybenzoate dehydrogenase [Escherichia coli CFT073]	248	29.21	267	6.00E-27	103
549477113	YP_008610807.1	gluconate 2-dehydrogenase	257	3823549	3824322	+	VFG0934	2,3-dihydro-2,3-dihydroxybenzoate dehydrogenase [Escherichia coli CFT073]	248	27.03	259	1.00E-22	91.3
549477225	YP_008610919.1	3-ketoacyl-ACP reductase	238	3925225	3925941	+	VFG0934	2,3-dihydro-2,3-dihydroxybenzoate dehydrogenase [Escherichia coli CFT073]	248	27.72	202	9.00E-23	90.9
549477426	YP_008611121.1	dioxygenase	278	4155566	4156402	-	VFG0934	2,3-dihydro-2,3-dihydroxybenzoate dehydrogenase [Escherichia coli CFT073]	248	24.04	208	4.00E-16	72.4
549477489	YP_008611185.1	sorbitol-6-phosphate 2-dehydrogenase	266	4232232	4233032	-	VFG0934	2,3-dihydro-2,3-dihydroxybenzoate dehydrogenase [Escherichia coli CFT073]	248	28.46	253	1.00E-20	85.1
549477777	YP_008611476.1	dehydrogenase	299	4565927	4566826	-	VFG0934	2,3-dihydro-2,3-dihydroxybenzoate dehydrogenase [Escherichia coli CFT073]	248	26.25	259	2.00E-18	79.3
549477842	YP_008611541.1	short-chain dehydrogenase	255	4634383	4635150	+	VFG0934	2,3-dihydro-2,3-dihydroxybenzoate dehydrogenase [Escherichia coli CFT073]	248	29.57	186	2.00E-12	61.6
549477848	YP_008611549.1	short-chain dehydrogenase	289	4640222	4641091	+	VFG0934	2,3-dihydro-2,3-dihydroxybenzoate dehydrogenase [Escherichia coli CFT073]	248	26.67	255	6.00E-23	92
549477864	YP_008611566.1	cytochrome C553	253	4651023	4651784	+	VFG0934	2,3-dihydro-2,3-dihydroxybenzoate dehydrogenase [Escherichia coli CFT073]	248	27.75	191	1.00E-14	67.8
549477952	YP_008611654.1	3-ketoacyl-ACP reductase	251	4719331	4720086	+	VFG0934	2,3-dihydro-2,3-dihydroxybenzoate dehydrogenase [Escherichia coli CFT073]	248	26.15	195	1.00E-11	59.3
549475284	YP_008608952.1	glucose-1-phosphate thymidylyltransferase	295	2114959	2115846	-	VFG0964	UDP-glucose pyrophosphorylase [Streptococcus pyogenes SF370]	304	27.04	270	2.00E-14	68.9
549475568	YP_008609239.1	UTP--glucose-1-phosphate uridylyltransferase	293	2387008	2387889	+	VFG0964	UDP-glucose pyrophosphorylase [Streptococcus pyogenes SF370]	304	51.04	288	4.00E-100	296
549475739	YP_008609411.1	UTP--glucose-1-phosphate uridylyltransferase	294	2528659	2529543	-	VFG0964	UDP-glucose pyrophosphorylase [Streptococcus pyogenes SF370]	304	53.95	291	2.00E-110	323
549477788	YP_008611487.1	UTP--glucose-1-phosphate uridylyltransferase	295	4576312	4577199	-	VFG0964	UDP-glucose pyrophosphorylase [Streptococcus pyogenes SF370]	304	46.18	288	1.00E-90	272
549475201	YP_008608869.1	aminopeptidase	464	2032249	2033643	-	VFG0974	C5a peptidase precursor [Streptococcus pyogenes SF370]	1181	37.19	121	1.00E-11	63.5
549477627	YP_008611323.1	hypothetical protein	1466	4414259	4418659	-	VFG0974	C5a peptidase precursor [Streptococcus pyogenes SF370]	1181	30.09	884	9.00E-90	315
549477638	YP_008611334.1	hypothetical protein	1298	4433651	4437547	-	VFG0974	C5a peptidase precursor [Streptococcus pyogenes SF370]	1181	32.49	671	2.00E-81	288
549477685	YP_008611382.1	peptidase S8	746	4477044	4479284	-	VFG0974	C5a peptidase precursor [Streptococcus pyogenes SF370]	1181	28.27	527	2.00E-41	159
549475148	YP_008608815.1	racemase	234	1976808	1977512	+	VFG1024	unknown [Shigella flexneri (serotype 2a) YSH6000]	229	34.81	135	3.00E-19	80.5
549474952	YP_008608614.1	tyrosine recombinase XerC	260	1779415	1780197	+	VFG1028	Tn21 integrase IntI1 [Shigella flexneri (serotype 2a) YSH6000]	337	28.2	266	3.00E-22	90.5
549476476	YP_008610159.1	tyrosine recombinase XerD	297	3190468	3191361	-	VFG1028	Tn21 integrase IntI1 [Shigella flexneri (serotype 2a) YSH6000]	337	28.28	297	2.00E-24	98.2
549474313	YP_008607972.1	MFS transporter	403	1145585	1146796	+	VFG1036	tetracycline resistance protein TetA(B) [Shigella flexneri (serotype 2a) YSH6000]	401	25.14	362	1.00E-12	65.5
549476127	YP_008609802.1	MFS transporter	423	2859172	2860443	+	VFG1036	tetracycline resistance protein TetA(B) [Shigella flexneri (serotype 2a) YSH6000]	401	23.43	414	1.00E-11	62
549477837	YP_008611536.1	multidrug MFS transporter	399	4629893	4631092	-	VFG1036	tetracycline resistance protein TetA(B) [Shigella flexneri (serotype 2a) YSH6000]	401	26	400	4.00E-26	104
549473486	YP_008607133.1	adenosine deaminase	189	360206	360775	+	VFG1038	putative transcriptional regulator TetD [Shigella flexneri (serotype 2a) YSH6000]	138	34.31	102	2.00E-14	64.3
549474103	YP_008607759.1	transcriptional regulator	294	933783	934667	+	VFG1038	putative transcriptional regulator TetD [Shigella flexneri (serotype 2a) YSH6000]	138	40.78	103	5.00E-23	90.1
549475756	YP_008609428.1	AraC family transcriptional regulator	295	2541621	2542508	-	VFG1038	putative transcriptional regulator TetD [Shigella flexneri (serotype 2a) YSH6000]	138	35.35	99	1.00E-16	72
549477935	YP_008611637.1	AraC family transcriptional regulator	282	4706406	4707254	-	VFG1038	putative transcriptional regulator TetD [Shigella flexneri (serotype 2a) YSH6000]	138	36.54	104	2.00E-17	74.3
549473343	YP_008606985.1	iron ABC transporter ATPase	487	217961	219424	+	VFG1042	ATP-binding protein FecE [Shigella flexneri (serotype 2a) YSH6000]	255	38.43	255	6.00E-55	184
549473627	YP_008607275.1	ABC transporter ATP-binding protein	307	487849	488772	+	VFG1042	ATP-binding protein FecE [Shigella flexneri (serotype 2a) YSH6000]	255	31.47	251	1.00E-30	114
549473728	YP_008607377.1	ABC transporter ATP-binding protein	627	582770	584653	+	VFG1042	ATP-binding protein FecE [Shigella flexneri (serotype 2a) YSH6000]	255	29.28	222	1.00E-14	70.9
549473926	YP_008607580.1	iron ABC transporter ATP-binding protein	255	772003	772770	+	VFG1042	ATP-binding protein FecE [Shigella flexneri (serotype 2a) YSH6000]	255	41.67	252	7.00E-62	195
549474926	YP_008608588.1	ABC transporter	311	1752383	1753318	+	VFG1042	ATP-binding protein FecE [Shigella flexneri (serotype 2a) YSH6000]	255	26.82	220	3.00E-23	93.6
549477899	YP_008611601.1	ABC transporter	258	4679498	4680274	+	VFG1042	ATP-binding protein FecE [Shigella flexneri (serotype 2a) YSH6000]	255	27.73	238	2.00E-24	96.3
549477195	YP_008610889.1	iron ABC transporter permease	351	3897098	3898153	-	VFG1043	FecD [Shigella flexneri (serotype 2a) YSH6000]	318	38.67	300	5.00E-58	190
549473927	YP_008607581.1	ferrichrome ABC transporter substrate-binding protein	342	772967	773995	+	VFG1045	FecB [Shigella flexneri (serotype 2a) YSH6000]	300	25.38	264	1.00E-15	72.8
549477197	YP_008610891.1	iron siderophore-binding protein	323	3899354	3900325	+	VFG1045	FecB [Shigella flexneri (serotype 2a) YSH6000]	300	28.16	277	3.00E-25	100
549476788	YP_008610477.1	hypothetical protein	229	3480606	3481295	-	VFG1066	unknown [Shigella flexneri (serotype 2a) YSH6000]	163	42.11	114	6.00E-29	105
549473485	YP_008607132.1	helicase SNF	1065	356809	360006	+	VFG1093	helicase, putative [Vibrio cholerae N16961]	940	27.1	524	2.00E-31	129
549476581	YP_008610266.1	ATP-dependent helicase	562	3286352	3288040	+	VFG1093	helicase, putative [Vibrio cholerae N16961]	940	24.51	506	4.00E-20	90.9
549477635	YP_008611331.1	ATP-dependent helicase	942	4429661	4432489	+	VFG1093	helicase, putative [Vibrio cholerae N16961]	940	29.21	534	9.00E-53	196
549474086	YP_008607742.1	transcriptional regulator	241	915051	915776	-	VFG1108	conserved hypothetical protein [Vibrio cholerae N16961]	278	29.59	196	1.00E-17	76.3
549474262	YP_008607921.1	RpiR family transcriptional regulator	282	1098111	1098959	-	VFG1108	conserved hypothetical protein [Vibrio cholerae N16961]	278	28.74	254	3.00E-31	116
549476095	YP_008609769.1	RpiR family transcriptional regulator	292	2830940	2831818	-	VFG1108	conserved hypothetical protein [Vibrio cholerae N16961]	278	31.02	245	5.00E-31	115
549477539	YP_008611235.1	hypothetical protein	559	4287125	4288804	-	VFG1108	conserved hypothetical protein [Vibrio cholerae N16961]	278	27.63	257	2.00E-18	82.4
549475023	YP_008608687.1	dihydrodipicolinate synthase	289	1846291	1847160	+	VFG1109	N-acetylneuraminate lyase, putative [Vibrio cholerae N16961]	298	28.93	280	9.00E-29	109
549474611	YP_008608270.1	C4-dicarboxylate ABC transporter	653	1433974	1435935	+	VFG1110	conserved hypothetical protein [Vibrio cholerae N16961]	427	29.45	163	1.00E-11	63.2
549477430	YP_008611125.1	C4-dicarboxylate ABC transporter permease	423	4159150	4160421	-	VFG1110	conserved hypothetical protein [Vibrio cholerae N16961]	427	46.28	417	5.00E-108	326
549477876	YP_008611578.1	membrane protein	431	4659026	4660321	-	VFG1110	conserved hypothetical protein [Vibrio cholerae N16961]	427	39.43	421	4.00E-86	270
549477877	YP_008611579.1	C4-dicarboxylate ABC transporter permease	181	4660318	4660863	-	VFG1111	conserved hypothetical protein [Vibrio cholerae N16961]	173	26.9	145	2.00E-12	59.3
549477432	YP_008611127.1	C4-dicarboxylate ABC transporter	333	4161008	4162009	-	VFG1112	C4-dicarboxylate-binding periplasmic protein [Vibrio cholerae N16961]	321	26.67	315	1.00E-37	136
549477878	YP_008611580.1	C4-dicarboxylate ABC transporter substrate-binding protein	334	4660878	4661882	-	VFG1112	C4-dicarboxylate-binding periplasmic protein [Vibrio cholerae N16961]	321	31.27	291	2.00E-42	148
549477536	YP_008611232.1	N-acetylmannosamine-6-phosphate 2-epimerase	227	4283874	4284557	-	VFG1114	conserved hypothetical protein [Vibrio cholerae N16961]	240	35.65	216	5.00E-35	124
549474030	YP_008607685.1	xylose repressor	399	855037	856236	-	VFG1115	ROK family protein [Vibrio cholerae N16961]	287	29.8	151	3.00E-12	63.2

549475831	YP_008609505.1	ROK family transcriptional regulator	307	2603144	2604067	-	VFG1115	ROK family protein [Vibrio cholerae N16961]	287	25.33	300	5.00E-13	64.7
549476605	YP_008610290.1	glucokinase	321	3303681	3304646	-	VFG1115	ROK family protein [Vibrio cholerae N16961]	287	28.43	313	5.00E-17	76.3
549477292	YP_008610986.1	N-acetylglucosamine-6-phosphate deacetylase	391	3990388	3991563	+	VFG1116	N-acetylglucosamine-6-phosphate deacetylase [Vibrio cholerae N16961]	378	32.45	379	7.00E-40	144
549477515	YP_008611211.1	N-acetylglucosamine-6-phosphate deacetylase	387	4262339	4263502	-	VFG1116	N-acetylglucosamine-6-phosphate deacetylase [Vibrio cholerae N16961]	378	28.9	346	4.00E-23	95.9
549473098	YP_008607789.1	ABC transporter	248	962673	963419	+	VFG1206	iron(III) ABC transporter, ATP-binding protein [Neisseria meningitidis MC58 (serogroup B)]	352	35.66	244	7.00E-42	145
549473126	YP_008610203.1	ABC transporter	240	3225806	3226528	-	VFG1206	iron(III) ABC transporter, ATP-binding protein [Neisseria meningitidis MC58 (serogroup B)]	352	36.44	236	2.00E-47	159
549473145	YP_008611754.1	ABC transporter	231	4796891	4797586	+	VFG1206	iron(III) ABC transporter, ATP-binding protein [Neisseria meningitidis MC58 (serogroup B)]	352	32.35	204	3.00E-23	93.2
549473279	YP_008606919.1	cobalt ABC transporter ATP-binding protein	279	156629	157468	+	VFG1206	iron(III) ABC transporter, ATP-binding protein [Neisseria meningitidis MC58 (serogroup B)]	352	34.05	232	1.00E-32	121
549473280	YP_008606920.1	cobalt ABC transporter ATP-binding protein	289	157444	158313	+	VFG1206	iron(III) ABC transporter, ATP-binding protein [Neisseria meningitidis MC58 (serogroup B)]	352	33.04	227	2.00E-32	120
549473516	YP_008607163.1	multidrug ABC transporter ATPase	292	392975	393853	+	VFG1206	iron(III) ABC transporter, ATP-binding protein [Neisseria meningitidis MC58 (serogroup B)]	352	28.94	235	1.00E-19	84
549473849	YP_008607501.1	ABC transporter	511	694480	696015	+	VFG1206	iron(III) ABC transporter, ATP-binding protein [Neisseria meningitidis MC58 (serogroup B)]	352	31.25	288	4.00E-25	102
549473855	YP_008607507.1	heme ABC transporter ATP-binding protein	517	700373	701926	-	VFG1206	iron(III) ABC transporter, ATP-binding protein [Neisseria meningitidis MC58 (serogroup B)]	352	29.54	237	2.00E-17	80.5
549473885	YP_008607539.1	bacitracin ABC transporter ATP-binding protein	307	737668	738591	+	VFG1206	iron(III) ABC transporter, ATP-binding protein [Neisseria meningitidis MC58 (serogroup B)]	352	29.07	227	4.00E-24	97.1
549474311	YP_008607970.1	ABC transporter ATP-binding protein	249	1143523	1144272	+	VFG1206	iron(III) ABC transporter, ATP-binding protein [Neisseria meningitidis MC58 (serogroup B)]	352	30.42	240	7.00E-28	107
549474356	YP_008608015.1	sugar ABC transporter ATP-binding protein	369	1189982	1191091	-	VFG1206	iron(III) ABC transporter, ATP-binding protein [Neisseria meningitidis MC58 (serogroup B)]	352	35.56	239	7.00E-47	162
549474377	YP_008608036.1	sodium ABC transporter ATP-binding protein	299	1210092	1210991	+	VFG1206	iron(III) ABC transporter, ATP-binding protein [Neisseria meningitidis MC58 (serogroup B)]	352	29.65	226	5.00E-26	102
549474491	YP_008608150.1	antibiotic ABC transporter ATP-binding protein	310	1315942	1316874	+	VFG1206	iron(III) ABC transporter, ATP-binding protein [Neisseria meningitidis MC58 (serogroup B)]	352	27.5	240	1.00E-25	101
549474544	YP_008608203.1	peptide ABC transporter ATP-binding protein	341	1371331	1372356	+	VFG1206	iron(III) ABC transporter, ATP-binding protein [Neisseria meningitidis MC58 (serogroup B)]	352	28.09	235	9.00E-25	99.8
549474613	YP_008608272.1	hypothetical protein	275	1436504	1437331	+	VFG1206	iron(III) ABC transporter, ATP-binding protein [Neisseria meningitidis MC58 (serogroup B)]	352	28.84	215	2.00E-17	77.4
549474626	YP_008608285.1	peptide ABC transporter ATP-binding protein	337	1449425	1450438	+	VFG1206	iron(III) ABC transporter, ATP-binding protein [Neisseria meningitidis MC58 (serogroup B)]	352	36.63	243	7.00E-36	131
549474663	YP_008608322.1	ABC transporter ATP-binding protein	248	1488090	1488836	-	VFG1206	iron(III) ABC transporter, ATP-binding protein [Neisseria meningitidis MC58 (serogroup B)]	352	36.19	210	1.00E-36	131
549475032	YP_008608696.1	heme ABC transporter ATP-binding protein	511	1855682	1857217	+	VFG1206	iron(III) ABC transporter, ATP-binding protein [Neisseria meningitidis MC58 (serogroup B)]	352	28.12	256	1.00E-21	92.8
549475464	YP_008609133.1	heme ABC transporter ATP-binding protein	539	2297733	2299352	+	VFG1206	iron(III) ABC transporter, ATP-binding protein [Neisseria meningitidis MC58 (serogroup B)]	352	24.17	240	1.00E-12	65.9
549475558	YP_008609228.1	spermidine/putrescine ABC transporter ATP-binding protein	368	2377885	2378991	-	VFG1206	iron(III) ABC transporter, ATP-binding protein [Neisseria meningitidis MC58 (serogroup B)]	352	35.41	305	6.00E-58	192
549475697	YP_008609369.1	ABC transporter ATP-binding protein	304	2498578	2499492	-	VFG1206	iron(III) ABC transporter, ATP-binding protein [Neisseria meningitidis MC58 (serogroup B)]	352	30.04	233	1.00E-24	98.6
549476018	YP_008609692.1	hypothetical protein	215	2757570	2758217	-	VFG1206	iron(III) ABC transporter, ATP-binding protein [Neisseria meningitidis MC58 (serogroup B)]	352	29.89	184	7.00E-15	68.9
549476080	YP_008609754.1	peptide ABC transporter ATP-binding protein	320	2815441	2816403	-	VFG1206	iron(III) ABC transporter, ATP-binding protein [Neisseria meningitidis MC58 (serogroup B)]	352	36.61	224	8.00E-37	134
549476081	YP_008609755.1	peptide ABC transporter ATP-binding protein	337	2816400	2817413	-	VFG1206	iron(III) ABC transporter, ATP-binding protein [Neisseria meningitidis MC58 (serogroup B)]	352	29.32	249	2.00E-27	107
549476820	YP_008610509.1	arginine ABC transporter ATP-binding protein	245	3517702	3518439	-	VFG1206	iron(III) ABC transporter, ATP-binding protein [Neisseria meningitidis MC58 (serogroup B)]	352	36.24	229	2.00E-42	146
549476989	YP_008610682.1	multidrug ABC transporter ATP-binding protein	237	3705161	3705874	-	VFG1206	iron(III) ABC transporter, ATP-binding protein [Neisseria meningitidis MC58 (serogroup B)]	352	27.31	216	1.00E-23	94.4
549477032	YP_008610726.1	spermidine/putrescine ABC transporter ATP-binding protein	256	3746360	3747130	+	VFG1206	iron(III) ABC transporter, ATP-binding protein [Neisseria meningitidis MC58 (serogroup B)]	352	40.72	221	1.00E-46	158
549477246	YP_008610940.1	glycine/betaine ABC transporter ATPase	381	3945543	3946688	-	VFG1206	iron(III) ABC transporter, ATP-binding protein [Neisseria meningitidis MC58 (serogroup B)]	352	39.66	237	8.00E-51	173
549477250	YP_008610944.1	glycine/betaine ABC transporter ATP-binding protein	318	3951113	3952069	-	VFG1206	iron(III) ABC transporter, ATP-binding protein [Neisseria meningitidis MC58 (serogroup B)]	352	40.35	228	9.00E-49	166
549477309	YP_008611003.1	phosphate ABC transporter ATP-binding protein	343	4007472	4008503	-	VFG1206	iron(III) ABC transporter, ATP-binding protein [Neisseria meningitidis MC58 (serogroup B)]	352	34.36	227	8.00E-36	131
549477323	YP_008611017.1	cell division protein FtsE	228	4021773	4022459	-	VFG1206	iron(III) ABC transporter, ATP-binding protein [Neisseria meningitidis MC58 (serogroup B)]	352	30.5	200	1.00E-29	111
549477760	YP_008611459.1	bacitracin ABC transporter ATP-binding protein	303	4549971	4550882	-	VFG1206	iron(III) ABC transporter, ATP-binding protein [Neisseria meningitidis MC58 (serogroup B)]	352	30.52	249	2.00E-22	92.4
549477808	YP_008611507.1	D-ribose transporter ATP binding protein	500	4597140	4598642	-	VFG1206	iron(III) ABC transporter, ATP-binding protein [Neisseria meningitidis MC58 (serogroup B)]	352	28.35	254	3.00E-22	94.4
549478057	YP_008611761.1	peptide ABC transporter ATP-binding protein	245	4804231	4804968	-	VFG1206	iron(III) ABC transporter, ATP-binding protein [Neisseria meningitidis MC58 (serogroup B)]	352	35.78	232	2.00E-45	154
549478064	YP_008611768.1	glycine/betaine ABC transporter ATP-binding protein	408	4811247	4812473	-	VFG1206	iron(III) ABC transporter, ATP-binding protein [Neisseria meningitidis MC58 (serogroup B)]	352	40.85	235	9.00E-51	174
549478101	YP_008611805.1	peptide ABC transporter ATP-binding protein	227	4856630	4857313	-	VFG1206	iron(III) ABC transporter, ATP-binding protein [Neisseria meningitidis MC58 (serogroup B)]	352	33.18	220	1.00E-28	108
549473906	YP_008607560.1	hypothetical protein	417	751928	753181	+	VFG1213	two-component sensor PilS [Pseudomonas aeruginosa PAO1]	530	26.7	221	1.00E-16	78.2
549474331	YP_008607990.1	histidine kinase	383	1163145	1164296	-	VFG1213	two-component sensor PilS [Pseudomonas aeruginosa PAO1]	530	29.33	208	2.00E-15	74.3
549474412	YP_008608071.1	hypothetical protein	412	1239376	1240614	+	VFG1213	two-component sensor PilS [Pseudomonas aeruginosa PAO1]	530	32.72	217	2.00E-24	101
549474697	YP_008608356.1	histidine kinase	628	1527588	1529474	+	VFG1213	two-component sensor PilS [Pseudomonas aeruginosa PAO1]	530	27.35	223	9.00E-20	89.4
549474711	YP_008608371.1	histidine kinase	497	1543274	1544767	-	VFG1213	two-component sensor PilS [Pseudomonas aeruginosa PAO1]	530	30.66	212	3.00E-17	80.9
549474715	YP_008608375.1	hypothetical protein	529	1549097	1550686	-	VFG1213	two-component sensor PilS [Pseudomonas aeruginosa PAO1]	530	33.03	221	2.00E-19	87.8
549475205	YP_008608873.1	hypothetical protein	474	2037273	2038697	+	VFG1213	two-component sensor PilS [Pseudomonas aeruginosa PAO1]	530	31.82	220	5.00E-27	110
549475582	YP_008609253.1	hypothetical protein	421	2398034	2399299	+	VFG1213	two-component sensor PilS [Pseudomonas aeruginosa PAO1]	530	24.55	220	1.00E-16	78.2
549475691	YP_008609363.1	sporulation kinase	478	2492742	2494178	+	VFG1213	two-component sensor PilS [Pseudomonas aeruginosa PAO1]	530	28.57	217	3.00E-22	96.3
549475766	YP_008609438.1	hypothetical protein	240	2550807	2551529	-	VFG1213	two-component sensor PilS [Pseudomonas aeruginosa PAO1]	530	28.44	225	2.00E-22	92.8
549475902	YP_008609576.1	hypothetical protein	350	2661975	2663027	-	VFG1213	two-component sensor PilS [Pseudomonas aeruginosa PAO1]	530	29.58	213	8.00E-15	72
549476156	YP_008609833.1	ATPase	384	2899380	2900534	-	VFG1213	two-component sensor PilS [Pseudomonas aeruginosa PAO1]	530	30.28	218	5.00E-21	91.3
549477311	YP_008611005.1	histidine kinase	475	4009233	4010660	+	VFG1213	two-component sensor PilS [Pseudomonas aeruginosa PAO1]	530	29.49	217	6.00E-23	98.2
549474041	YP_008607696.1	transcriptional regulator	235	868557	869264	+	VFG1214	two-component response regulator PilR [Pseudomonas aeruginosa PAO1]	445	35.19	108	2.00E-13	65.1
549477081	YP_008610775.1	ATPase AAA	467	3800410	3801813	+	VFG1214	two-component response regulator PilR [Pseudomonas aeruginosa PAO1]	445	35.95	420	9.00E-71	231
549475094	YP_008608760.1	chemotaxis protein CheY	119	1918036	1918395	+	VFG1226	twitching motility protein PilH [Pseudomonas aeruginosa PAO1]	121	37.19	121	2.00E-21	81.6
549474695	YP_008608354.1	methyl-accepting chemotaxis protein	602	1525524	1527332	+	VFG1228	twitching motility protein PilJ [Pseudomonas aeruginosa PAO1]	682	31.82	220	1.00E-26	111
549475928	YP_008609602.1	hypothetical protein	562	2686911	2688599	-	VFG1228	twitching motility protein PilJ [Pseudomonas aeruginosa PAO1]	682	33.86	381	4.00E-41	154
549476128	YP_008609803.1	hypothetical protein	482	2860519	2861967	-	VFG1228	twitching motility protein PilJ [Pseudomonas aeruginosa PAO1]	682	31.03	232	3.00E-19	87.4
549477242	YP_008610936.1	hypothetical protein	565	3938125	3939822	+	VFG1228	twitching motility protein PilJ [Pseudomonas aeruginosa PAO1]	682	29.78	403	1.00E-28	117
549477312	YP_008611006.1	hypothetical protein	433	4010827	4012128	+	VFG1228	twitching motility protein PilJ [Pseudomonas aeruginosa PAO1]	682	32.18	202	3.00E-22	95.9
549477695	YP_008611393.1	methyl-accepting chemotaxis protein	430	4488997	4490289	-	VFG1228	twitching motility protein PilJ [Pseudomonas aeruginosa PAO1]	682	28.11	338	3.00E-26	108
549477987	YP_008611689.1	methyl-accepting chemotaxis protein	567	4741585	4743288	+	VFG1228	twitching motility protein PilJ [Pseudomonas aeruginosa PAO1]	682	30.51	390	1.00E-32	129
549476154	YP_008609831.1	amino acid transporter	207	2892069	2892692	+	VFG1235	probable chemotaxis protein [Pseudomonas aeruginosa PAO1]	203	27.01	174	9.00E-11	55.5
549474966	YP_008608628.1	flagellar basal body rod modification protein FlgD	211	1791955	1792590	+	VFG1237	flagellar basal-body rod modification protein FlgD [Pseudomonas aeruginosa PAO1]	237	40	75	7.00E-15	67.4
549477645	YP_008611342.1	flagellar basal body rod protein FlgG	274	4440963	4441787	-	VFG1240	flagellar basal-body rod protein FlgG [Pseudomonas aeruginosa PAO1]	261	35.59	281	2.00E-33	121
549477349	YP_008611044.1	flagellar hook protein FlgK	532	4049203	4050801	-	VFG1244	flagellar hook-associated protein 1 FlgK [Pseudomonas aeruginosa PAO1]	683	28.42	373	1.00E-25	107
549477348	YP_008611043.1	flagellar hook-associated protein FlgL	287	4048328	4049191	-	VFG1245	flagellar hook-associated protein type 3 FlgL [Pseudomonas aeruginosa PAO1]	439	29.19	185	3.00E-19	83.6
549476226	YP_008609905.1	transcriptional regulator	460	2961743	2963125	-	VFG1249	two-component response regulator [Pseudomonas aeruginosa PAO1]	473	47.37	228	5.00E-61	206
549474958	YP_008608620.1	flagellar hook-basal body protein FliE	102	1784524	1784832	+	VFG1250	flagellar hook-basal body complex protein FliE [Pseudomonas aeruginosa PAO1]	109	46.67	75	8.00E-17	68.6
549474960	YP_008608622.1	flagellar motor switch protein FliG	338	1786509	1787525	+	VFG1252	flagellar motor switch protein FliG [Pseudomonas aeruginosa PAO1]	338	33.64	330	4.00E-69	219
549474972	YP_008608634.1	flagellar motor switch protein FliN	408	1795590	1796816	+	VFG1257	flagellar motor switch protein FliN [Pseudomonas aeruginosa PAO1]	157	57.47	87	9.00E-30	110
549474976	YP_008608638.1	flagellar biosynthesis protein FliQ	89	1798593	1798862	+	VFG1260	flagellar biosynthetic protein FliQ [Pseudomonas aeruginosa PAO1]	89	38.2	89	2.00E-20	77
549474934	YP_008608596.1	signal recognition particle protein Srp54	449	1762574	1763923	+	VFG1264	flagellar biosynthesis protein FlhF [Pseudomonas aeruginosa PAO1]	429	30.81	198	2.00E-15	74.3
549474981	YP_008608643.1	cobyrinic acid a,c-diamide synthase	290	1804142	1805014	+	VFG1265	flagellar synthesis regulator FleN [Pseudomonas aeruginosa PAO1]	280	32.57	218	3.00E-35	127
549476782	YP_008610471.1	septum site-determining protein MinD	267	3476037	3476840	-	VFG1265	flagellar synthesis regulator FleN [Pseudomonas aeruginosa PAO1]	280	27.35	234	1.00E-20	85.5
549473680	YP_008607329.1	ABC transporter	580	533428	535170	+	VFG1269	cyclolysin secretion ATP-binding protein [Bordetella pertussis Tohama I]	712	30.5	577	5.00E-65	224
549473681	YP_008607330.1	hypothetical protein	615	535151	536998	+	VFG1269	cyclolysin secretion ATP-binding protein [Bordetella pertussis Tohama I]	712	29.6	571	2.00E-77	259
549474189	YP_008607847.1	multidrug ABC transporter ATP-binding protein	582	1008886	1010634	+	VFG1269	cyclolysin secretion ATP-binding protein [Bordetella pertussis Tohama I]	712	34.29	560	1.00E-104	331
549474351	YP_008608010.1	multidrug ABC transporter ATP-binding protein	585	1183568	1185325	+	VFG1269	cyclolysin secretion ATP-binding protein [Bordetella pertussis Tohama I]	712	33	494	2.00E-71	242
549474442	YP_008608101.1	ABC transporter ATP-binding protein	264	1266332	1267126	+	VFG1269	cyclolysin secretion ATP-binding protein [Bordetella pertussis Tohama I]	712	30.91	220	3.00E-24	99.4
549474763	YP_008608424.1	multidrug ABC transporter ATP-binding protein	584	1590458	1592212	+	VFG1269	cyclolysin secretion ATP-binding protein [Bordetella pertussis Tohama I]	712	36.91	550	7.00E-107	337
549475091	YP_008608757.1	multidrug ABC transporter ATP-binding protein	599	1914755	1916554	+	VFG1269	cyclolysin secretion ATP-binding protein [Bordetella pertussis Tohama I]	712	30.7	544	3.00E-68	234
549476105	YP_008609779.1	multidrug ABC transporter ATP-binding protein	571	2838511	2840226	-	VFG1269	cyclolysin secretion ATP-binding protein [Bordetella pertussis Tohama I]	712	32.17	575	7.00E-88	286
549477239	YP_008610933.1	hypothetical protein	290	3935861	3936733	-	VFG1269	cyclolysin secretion ATP-binding protein [Bordetella pertussis Tohama I]	712	25	192	4.00E-14	68.6
549474245	YP_008607903.1	glycosyl transferase	470	1073555	1074967	+	VFG1285	intercellular adhesion protein A [Staphylococcus aureus MW2]	412	26.35	463	2.00E-28	112
549477438	YP_008611133.1	glycosyl transferase family 2	449	4166792	4168141	-	VFG1285	intercellular adhesion protein A [Staphylococcus aureus MW2]	412	25.9	363	7.00E-28	110
549477437	YP_008611132.1	hypothetical protein	319	4165857	4166816	-	VFG1287	intercellular adhesion protein B [Staphylococcus aureus MW2]	290	31.46	213	7.00E-22	90.1
549477606	YP_008611302.1	capsular polysaccharide biosynthesis protein	246	4372397	4373137	-	VFG1297	truncated capsular polysaccharide synthesis enzyme Cap5A [Staphylococcus aureus MW2]	171	38.65	163	3.00E-33	118
549477605	YP_008611301.1	tyrosine protein kinase	234	4371703	4372407	-	VFG1298	capsular polysaccharide synthesis enzyme Cap8B [Staphylococcus aureus MW2]	228	42.11	209	8.00E-63	196
549477604	YP_008611300.1	tyrosine protein phosphatase	254	4370840	4371604	-	VFG1299	capsular polysaccharide synthesis enzyme Cap8C [Staphylococcus aureus MW2]	254	40.55	254	2.00E-65	204
549477603	YP_008611299.1	polysaccharide biosynthesis protein EpsC	606	4368745	4370565	-	VFG1300	capsular polysaccharide synthesis enzyme Cap8D [Staphylococcus aureus MW2]	607	56.48	602	0	647
549475276	YP_008608944.1	UDP-N-acetylglucosamine 4,6-dehydratase	329	2107824	2108813	+	VFG1301	capsular polysaccharide synthesis enzyme Cap8E [Staphylococcus aureus MW2]	342	45.19	270	8.00E-73	229
549475275	YP_008608943.1	UDP-N-acetylglucosamine 2-epimerase	363	2106740	2107831	+	VFG1303	capsular polysaccharide synthesis enzyme Cap8G [Staphylococcus aureus MW2]	374	43.89	360	4.00E-104	312
549473525	YP_008607172.1	maltose O-acetyltransferase	186	401350	401910	+	VFG1306	capsular polysaccharide synthesis enzyme Cap8J [Staphylococcus aureus MW2]	185	40.78	103	2.00E-17	73.9
549477287	YP_008610981.1	acetyltransferase	177	3986174	3986707	-	VFG1306	capsular polysaccharide synthesis enzyme Cap8J [Staphylococcus aureus MW2]	185	38.2	89	2.00E-11	56.2
549477596	YP_008611292.1	hypothetical protein	420	4360627	4361889	-	VFG1307	capsular polysaccharide synthesis enzyme Cap8K [Staphylococcus aureus MW2]	412	25.84	356	1.00E-17	81.3
549477601	YP_008611297.1	UDP-galactose phosphate transferase	214	4366786	4367430	-	VFG1309	capsular polysaccharide synthesis enzyme Cap8M [Staphylococcus aureus MW2]	185	42.39	184	3.00E-47	154
549477595	YP_008611291.1	UDP-N-acetyl-D-glucosamine dehydrogenase	441	4358957	4360282	-	VFG1311	capsular polysaccharide synthesis enzyme Cap8O [Staphylococcus aureus MW2]	420	33.65	419	2.00E-76	244
549477614	YP_008611310.1	UDP-N-acetyl-D-mannosamine dehydrogenase	424	4387916	4389190	-	VFG1311	capsular polysaccharide synthesis enzyme Cap8O [Staphylococcus aureus MW2]	420	54.57	416	2.00E-163	467
549477406	YP_008611101.1	peptidase M4	549	4128425	4130074	-	VFG1314	zinc metalloproteinase aureolysin [Staphylococcus aureus MW2]	509	46.27	469	3.00E-123	372
549475785	YP_008609457.1	ABC transporter substrate-binding protein	380	2567056	2568198	+	VFG1330	laminin-binding surface protein [Streptococcus agalactiae 2603V/R]	306	32.34	167	1.00E-22	93.2
549477045	YP_008610739.1	adhesin	330	3755376	3756368	+	VFG1330	laminin-binding surface protein [Streptococcus agalactiae 2603V/R]	306	26.28	331	9.00E-34	124
549475285	YP_008608953.1	acetyltransferase	212	2115888	2116526	-	VFG1336	neuD protein [Streptococcus agalactiae 2603V/R]	209	28.64	206	5.00E-21	85.1
549477588	YP_008611284.1	hypothetical protein	374	4351023	4352147	-	VFG1336	neuD protein [Streptococcus agalactiae 2603V/R]	209	27.78	198	9.00E-15	69.3
549477804	YP_008611503.1	hypothetical protein	329	4593679	4594668	-	VFG1341	glycosyl transferase CpsJ(V) [Streptococcus agalactiae 2603V/R]	321	33	203	1.00E-25	102
549478120	YP_008611826.1	sporulation initiation inhibitor Soj	253	4875044	4875805	-	VFG1349	cpsD protein [Streptococcus agalactiae 2603V/R]	229	29.87	154	4.00E-11	57.8
549476206	YP_008609885.1	hypothetical protein	300	2945800	2946702	-	VFG1352	capsular polysaccharide biosynthesis protein CpsA [Streptococcus agalactiae 2603V/R]	485	30.96	197	1.00E-14	70.5
549477365	YP_008611060.1	LytR family transcriptional regulator	327	4063142	4064125	+	VFG1352	capsular polysaccharide biosynthesis protein CpsA [Streptococcus agalactiae 2603V/R]	485	32.62	233	7.00E-28	109
549478040	YP_008611743.1	transcriptional regulator	322	4786166	4787134	-	VFG1352	capsular polysaccharide biosynthesis protein CpsA [Streptococcus agalactiae 2603V/R]	485	28.3	212	3.00E-15	72.4
549477623	YP_008611319.1	hypothetical protein	656	4407148	4409118	-	VFG1356	endo-beta-N-acetylglucosaminidase [Streptococcus pneumoniae TIGR4]	658	33.33	210	2.00E-20	92
549474452	YP_008608111.1	transcriptional regulator	313	1275405	1276346	+	VFG1365	capsular polysaccharide biosynthesis protein Cps4A [Streptococcus pneumoniae TIGR4]	481	29.29	198	1.00E-13	67.8
549477382	YP_008611077.1	transcriptional regulator	308	4088548	4089474	+	VFG1365	capsular polysaccharide biosynthesis protein Cps4A [Streptococcus pneumoniae TIGR4]	481	31.58	247	7.00E-31	118
549477600	YP_008611296.1	hypothetical protein	375	4365564	4366691	-	VFG1372	capsular polysaccharide biosynthesis protein Cps4H [Streptococcus pneumoniae TIGR4]	372	27.1	310	3.00E-20	87.8
549477611	YP_008611307.1	glycosyl transferase	365	4384445	4385542	-	VFG1372	capsular polysaccharide biosynthesis protein Cps4H [Streptococcus pneumoniae TIGR4]	372	27.83	309	7.00E-21	89
549477687	YP_008611384.1	UDP-N-acetylglucosamine 2-epimerase	382	4480933	4482081	-	VFG1373	UDP-N-acetylglucosamine-2-epimerase [Streptococcus pneumoniae TIGR4]	365	62.78	360	2.00E-171	484
549475070	YP_008608735.1	transcriptional regulator	134	1898508	1898912	+	VFG1379	hspR [Mycobacterium tuberculosis H37Rv]	126	35.63	87	7.00E-12	55.8
549473423	YP_008607070.1	isocitrate lyase	427	304786	306069	+	VFG1381	aceA [Mycobacterium tuberculosis H37Rv]	428	69.52	420	0	609
549474240	YP_008607898.1	pyrroline-5-carboxylate reductase	272	1067839	1068657	+	VFG1384	proC [Mycobacterium tuberculosis H37Rv]	295	34.55	275	2.00E-42	146
549476502	YP_008610186.1	pyrroline-5-carboxylate reductase	281	3209514	3210359	+	VFG1384	proC [Mycobacterium tuberculosis H37Rv]	295	34.83	290	7.00E-33	120
549473672	YP_008607321.1	transcriptional regulator	236	526232	526942	-	VFG1386	phoP [Mycobacterium tuberculosis H37Rv]	247	37.93	232	1.00E-40	139
549474243	YP_008607901.1	hypothetical protein	557	1070836	1072509	+	VFG1386	phoP [Mycobacterium tuberculosis H37Rv]	247	30.51	118	2.00E-13	67
549475133	YP_008608800.1	PhoB family transcriptional regulator	227	1961223	1961906	+	VFG1386	phoP [Mycobacterium tuberculosis H37Rv]	247	38.12	223	3.00E-48	159
549476149	YP_008609826.1	response regulator	404	2885146	2886360	+	VFG1386	phoP [Mycobacterium tuberculosis H37Rv]	247	37.8	127	3.00E-16	74.3
549476892	YP_008610582.1	PhoP family transcriptional regulator	237	3599394	3600107	-	VFG1386	phoP [Mycobacterium tuberculosis H37Rv]	247	43.4	235	1.00E-60	191
549478098	YP_008611802.1	PhoP family transcriptional regulator	236	4850411	4851121	-	VFG1386	phoP [Mycobacterium tuberculosis H37Rv]	247	39.91	233	3.00E-53	172
549476199	YP_008609878.1	transcriptional regulator	227	2940787	2941470	-	VFG1389	hypothetical protein Rv0903c [Mycobacterium tuberculosis H37Rv]	236	42.08	202	4.00E-46	153
549477762	YP_008611461.1	chemotaxis protein CheY	225	4552604	4553281	-	VFG1390	hypothetical protein Rv0981 [Mycobacterium tuberculosis H37Rv]	230	36	225	3.00E-38	132
549477830	YP_008611529.1	PhoB family transcriptional regulator	229	4622128	4622817	+	VFG1390	hypothetical protein Rv0981 [Mycobacterium tuberculosis H37Rv]	230	30.36	224	8.00E-36	125
549477883	YP_008611585.1	XRE family transcriptional regulator	226	4666704	4667384	+	VFG1390	hypothetical protein Rv0981 [Mycobacterium tuberculosis H37Rv]	230	38.46	221	1.00E-41	141
549473293	YP_008606935.1	RNA polymerase sigma factor SigW	187	174237	174800	+	VFG1392	sigE [Mycobacterium tuberculosis H37Rv]	257	36.26	171	5.00E-33	117
549473735	YP_008607384.1	hypothetical protein	188	587867	588433	+	VFG1392	sigE [Mycobacterium tuberculosis H37Rv]	257	25.77	163	2.00E-14	65.9
549473995	YP_008607649.1	RNA polymerase sigma-70 factor	179	828692	829231	+	VFG1392	sigE [Mycobacterium tuberculosis H37Rv]	257	27.56	156	5.00E-16	70.1
549474346	YP_008608005.1	RNA polymerase sigma factor SigY	187	1181542	1182105	+	VFG1392	sigE [Mycobacterium tuberculosis H37Rv]	257	32.39	142	3.00E-18	76.6
549475630	YP_008609302.1	hypothetical protein	174	2442734	2443258	-	VFG1392	sigE [Mycobacterium tuberculosis H37Rv]	257	30.59	170	1.00E-16	71.6
549475749	YP_008609421.1	hypothetical protein	176	2537501	2538031	-	VFG1392	sigE [Mycobacterium tuberculosis H37Rv]	257	34.01	147	1.00E-15	68.9
549476122	YP_008609797.1	anthranilate phosphoribosyltransferase	341	2854690	2855715	-	VFG1398	trpD [Mycobacterium tuberculosis H37Rv]	370	33.82	346	3.00E-49	168
549475071	YP_008608736.1	glutamine synthetase	444	1898947	1900281	+	VFG1399	glnA1 [Mycobacterium tuberculosis H37Rv]	478	38.59	482	3.00E-102	313
549478021	YP_008611723.1	glutamine synthetase	448	4768558	4769904	+	VFG1399	glnA1 [Mycobacterium tuberculosis H37Rv]	478	35.67	471	2.00E-73	238
549473777	YP_008607427.1	alkyl hydroperoxide reductase	123	624686	625057	+	VFG1404	ahpC [Mycobacterium tuberculosis H37Rv]	195	31.19	109	2.00E-13	60.5

549477835	YP_008611534.1	alkyl hydroperoxide reductase subunit C	187	4627675	4628238	+	VFG1404	ahpC [Mycobacterium tuberculosis H37Rv]	195	33.9	177	2.00E-29	107
549476650	YP_008610335.1	RNA polymerase sigma factor RpoD	374	3344869	3345993	-	VFG1405	sigA [Mycobacterium tuberculosis H37Rv]	528	63.04	303	2.00E-130	385
549473533	YP_008607180.1	NADPH	328	408368	409354	-	VFG1408	mas [Mycobacterium tuberculosis H37Rv]	2111	29.84	248	4.00E-15	73.2
549475613	YP_008609285.1	quinone oxidoreductase	325	2425350	2426327	+	VFG1408	mas [Mycobacterium tuberculosis H37Rv]	2111	28.49	337	4.00E-29	116
549476829	YP_008610518.1	isopropylmalate isomerase	192	3530007	3530585	-	VFG1411	leuD [Mycobacterium tuberculosis H37Rv]	198	43.46	191	2.00E-39	133
549475217	YP_008608885.1	hypothetical protein	320	2051002	2051964	+	VFG1412	sigH [Mycobacterium tuberculosis H37Rv]	216	27.37	190	2.00E-13	64.7
549475782	YP_008609454.1	hypothetical protein	320	2564105	2565067	-	VFG1412	sigH [Mycobacterium tuberculosis H37Rv]	216	31.76	170	7.00E-19	80.5
549475916	YP_008609590.1	RNA polymerase sigma-70 factor	171	2674776	2675291	-	VFG1412	sigH [Mycobacterium tuberculosis H37Rv]	216	26.71	161	7.00E-11	55.1
549477690	YP_008611388.1	RNA polymerase	234	4485243	4485947	-	VFG1412	sigH [Mycobacterium tuberculosis H37Rv]	216	32.34	167	3.00E-19	80.5
549477911	YP_008611613.1	RNA polymerase	308	4690844	4691770	+	VFG1412	sigH [Mycobacterium tuberculosis H37Rv]	216	28.9	173	3.00E-12	61.2
549473396	YP_008607038.1	RNA polymerase sigma factor SigB	264	262159	262953	+	VFG1413	sigF [Mycobacterium tuberculosis H37Rv]	261	36.64	232	2.00E-52	171
549474871	YP_008608533.1	RNA polymerase sigma 70	270	1692566	1693378	+	VFG1413	sigF [Mycobacterium tuberculosis H37Rv]	261	32.14	224	2.00E-27	105
549476469	YP_008610152.1	RNA polymerase sigma70	252	3183629	3184387	-	VFG1413	sigF [Mycobacterium tuberculosis H37Rv]	261	36.09	230	4.00E-39	136
549476363	YP_008610044.1	aspartate decarboxylase	127	3091616	3091999	-	VFG1416	panD [Mycobacterium tuberculosis H37Rv]	139	51.22	123	7.00E-38	125
549476364	YP_008610045.1	pantoate--beta-alanine ligase	282	3092021	3092869	-	VFG1417	panC [Mycobacterium tuberculosis H37Rv]	309	46.46	198	1.00E-50	169
549476452	YP_008610135.1	superoxide dismutase	296	3172335	3173225	-	VFG1421	sodA [Mycobacterium tuberculosis H37Rv]	207	43.37	196	3.00E-55	177
549477541	YP_008611237.1	hypothetical protein	451	4291397	4292752	-	VFG1442	invasion protein IbeA [Escherichia coli]	456	24.77	440	3.00E-26	107
549473094	YP_008607519.1	DNA methyltransferase	416	715117	716367	-	VFG1454	putative DNA methylase [Escherichia coli 536]	310	32.24	183	3.00E-22	92.8
549476638	YP_008610323.1	zinc ABC transporter ATP-binding protein	263	3334279	3335070	-	VFG1496	putative ABC transporter ATP-binding protein [Escherichia coli 536]	244	34.51	226	7.00E-37	130
549475429	YP_008609098.1	DNA helicase	745	2267712	2269949	-	VFG1537	hypothetical protein [Escherichia coli 536]	1171	29.05	296	2.00E-16	80.1
549475090	YP_008608756.1	multidrug ABC transporter ATP-binding protein	587	1913002	1914765	+	VFG1557	HlyB protein [Escherichia coli 536]	707	30.96	575	8.00E-83	273
549477171	YP_008610865.1	iron ABC transporter ATP-binding protein	261	3875490	3876275	-	VFG1557	HlyB protein [Escherichia coli 536]	707	28.44	218	3.00E-12	62.8
549477780	YP_008611479.1	multidrug ABC transporter permease	588	4569222	4570988	+	VFG1557	HlyB protein [Escherichia coli 536]	707	32.63	567	1.00E-81	270
549477117	YP_008610811.1	oxidoreductase	197	3826211	3826804	-	VFG1561	hypothetical protein [Escherichia coli 536]	256	25	172	1.00E-12	61.2
549474026	YP_008607681.1	transcriptional regulator	227	851936	852619	+	VFG1563	hypothetical protein [Escherichia coli 536]	239	35.81	229	5.00E-49	160
549474256	YP_008607914.1	PhoP family transcriptional regulator	229	1091250	1091939	+	VFG1563	hypothetical protein [Escherichia coli 536]	239	37.05	224	3.00E-48	159
549474322	YP_008607981.1	LuxR family transcriptional regulator	233	1156046	1156747	+	VFG1563	hypothetical protein [Escherichia coli 536]	239	43.78	233	7.00E-59	186
549475918	YP_008609592.1	transcriptional regulator	230	2677518	2678210	-	VFG1563	hypothetical protein [Escherichia coli 536]	239	33.19	226	5.00E-45	150
549475925	YP_008609599.1	regulator	238	2684970	2685686	-	VFG1563	hypothetical protein [Escherichia coli 536]	239	37.07	232	7.00E-48	158
549476444	YP_008610127.1	PhoP family transcriptional regulator	238	3164589	3165305	-	VFG1563	hypothetical protein [Escherichia coli 536]	239	39.5	238	2.00E-54	175
549477887	YP_008611589.1	DeoR family transcriptional regulator	228	4669252	4669938	-	VFG1563	hypothetical protein [Escherichia coli 536]	239	36.44	225	2.00E-49	161
549478103	YP_008611807.1	transcriptional regulator	225	4858403	4859080	-	VFG1563	hypothetical protein [Escherichia coli 536]	239	36.12	227	1.00E-45	152
549474263	YP_008607922.1	gluconate transporter	447	1099173	1100516	+	VFG1583	hypothetical protein [Escherichia coli 536]	445	36.01	436	4.00E-82	260
549474759	YP_008608420.1	LysR family transcriptional regulator	294	1586402	1587286	+	VFG1584	hypothetical protein [Escherichia coli 536]	314	32	125	2.00E-12	63.2
549477027	YP_008610721.1	LysR family transcriptional regulator	292	3741991	3742869	-	VFG1584	hypothetical protein [Escherichia coli 536]	314	26.95	167	1.00E-12	63.5
549477982	YP_008611684.1	LysR family transcriptional regulator	302	4738877	4739785	+	VFG1584	hypothetical protein [Escherichia coli 536]	314	30.43	161	5.00E-14	67.8
549476052	YP_008609726.1	quaternary ammonium transporter	123	2788755	2789126	-	VFG1586	hypothetical protein [Escherichia coli 536]	109	32.35	102	3.00E-20	78.2
549473359	YP_008607001.1	multidrug transporter	104	230009	230323	+	VFG1587	hypothetical protein [Escherichia coli 536]	133	32	100	4.00E-11	53.1
549475780	YP_008609452.1	alcohol dehydrogenase	398	2561854	2563050	-	VFG1593	hypothetical protein [Escherichia coli 536]	384	30.95	391	4.00E-53	181
549477078	YP_008610772.1	NADH-dependent butanol dehydrogenase A	387	3797282	3798445	-	VFG1593	hypothetical protein [Escherichia coli 536]	384	26.9	290	3.00E-17	79.3
549477526	YP_008611222.1	alcohol dehydrogenase	397	4273449	4274642	-	VFG1593	hypothetical protein [Escherichia coli 536]	384	31.33	383	3.00E-52	178
549474735	YP_008608395.1	aminotransferase A	384	1567800	1568954	-	VFG1604	hypothetical protein [Escherichia coli 536]	392	22.13	366	3.00E-17	79
549476273	YP_008609953.1	hypothetical protein	393	3005221	3006402	-	VFG1604	hypothetical protein [Escherichia coli 536]	392	24.46	368	8.00E-19	84
549477084	YP_008610778.1	aminotransferase A	388	3803077	3804243	-	VFG1604	hypothetical protein [Escherichia coli 536]	392	23.31	369	1.00E-19	86.3
549477089	YP_008610783.1	cystathionine beta-lyase	389	3806817	3807986	+	VFG1604	hypothetical protein [Escherichia coli 536]	392	33.07	384	8.00E-81	253
549474713	YP_008608373.1	MarR family transcriptional regulator	146	1546582	1547022	+	VFG1649	SfaX protein [Escherichia coli 536]	166	26.09	115	7.00E-12	56.6
549475763	YP_008609435.1	multidrug ABC transporter ATP-binding protein	585	2548183	2549940	-	VFG1653	ABC transport protein [Escherichia coli 536]	1219	32.87	499	2.00E-57	206
549475872	YP_008609546.1	4-hydroxytetrahydrobiopterin dehydratase	392	2636398	2637576	-	VFG1661	hypothetical protein [Escherichia coli 536]	399	27.23	415	2.00E-36	135
549476205	YP_008609884.1	cobalamin biosynthesis protein	311	2944729	2945664	-	VFG1661	hypothetical protein [Escherichia coli 536]	399	24.55	224	3.00E-15	72
549477584	YP_008611280.1	ATP-binding protein	257	4346728	4347501	-	VFG1663	transposase Orf B, insertion element IS100 [Escherichia coli 536]	259	35.59	236	4.00E-45	152
549473232	YP_008606855.1	lysyl-tRNA synthetase	496	93050	94540	+	VFG1668	putative lysil-tRNA synthetase LysU [Escherichia coli 536]	498	50.3	495	7.00E-180	515
549473956	YP_008607610.1	asparaginyl-tRNA synthetase	463	803040	804431	+	VFG1668	putative lysil-tRNA synthetase LysU [Escherichia coli 536]	498	25.22	460	7.00E-17	79.3
549476739	YP_008610428.1	aspartyl-tRNA synthase	591	3434002	3435777	-	VFG1668	putative lysil-tRNA synthetase LysU [Escherichia coli 536]	498	24.49	294	3.00E-24	103
549473175	YP_008606797.1	arginine decarboxylase	502	39416	40924	+	VFG1670	putative cadaverin decarboxylase [Escherichia coli 536]	708	23.05	577	2.00E-25	107
549474789	YP_008608450.1	arginine decarboxylase	489	1616306	1617775	-	VFG1670	putative cadaverin decarboxylase [Escherichia coli 536]	708	25.45	554	6.00E-30	120
549474108	YP_008607764.1	MarR family transcriptional regulator	141	938443	938868	-	VFG1688	putative MarR-family transcriptional regulator [Escherichia coli 536]	174	27.61	134	3.00E-11	54.7
549477993	YP_008611695.1	MarR family transcriptional regulator	141	4746599	4747024	-	VFG1688	putative MarR-family transcriptional regulator [Escherichia coli 536]	174	29.69	128	3.00E-12	57.4
549473671	YP_008607320.1	sensor histidine kinase	591	524460	526235	-	VFG1701	Hypothetical protein [Escherichia coli CFT073]	490	29.15	247	1.00E-24	103
549473887	YP_008607541.1	histidine kinase	297	739355	740248	+	VFG1701	Hypothetical protein [Escherichia coli CFT073]	490	29.06	234	7.00E-24	97.8
549475917	YP_008609591.1	hypothetical protein	745	2675303	2677540	-	VFG1701	Hypothetical protein [Escherichia coli CFT073]	490	24.71	255	4.00E-17	81.6
549476443	YP_008610126.1	sensor histidine kinase	590	3162820	3164592	-	VFG1701	Hypothetical protein [Escherichia coli CFT073]	490	31.89	254	3.00E-31	123
549477761	YP_008611460.1	histidine kinase	459	4551228	4552607	-	VFG1701	Hypothetical protein [Escherichia coli CFT073]	490	23.42	316	5.00E-12	63.9
549477886	YP_008611588.1	histidine kinase	355	4668188	4669255	-	VFG1701	Hypothetical protein [Escherichia coli CFT073]	490	24.77	323	6.00E-22	93.2
549478007	YP_008611709.1	membrane protein	458	4754951	4756327	-	VFG1701	Hypothetical protein [Escherichia coli CFT073]	490	24.44	315	1.00E-25	105
549478102	YP_008611806.1	histidine kinase	333	4857400	4858401	-	VFG1701	Hypothetical protein [Escherichia coli CFT073]	490	23.19	332	9.00E-18	80.5
549473884	YP_008607538.1	hypothetical protein	200	736880	737482	+	VFG1702	Putative response regulator [Escherichia coli CFT073]	240	34.05	185	6.00E-30	109
549475619	YP_008609291.1	transcriptional regulator	234	2434313	2435017	-	VFG1702	Putative response regulator [Escherichia coli CFT073]	240	38.6	228	2.00E-48	159
549473500	YP_008607147.1	hypothetical protein	510	372030	373562	+	VFG1746	putative two-component response regulator [Yersinia pestis CO92]	210	29.34	167	2.00E-14	69.3
549475324	YP_008608993.1	long-chain fatty acid--CoA ligase	519	2160406	2161965	+	VFG1817	mbtA [Mycobacterium tuberculosis H37Rv]	565	26.05	522	7.00E-39	145
549476951	YP_008610642.1	acetyl-CoA synthetase	572	3664513	3666231	-	VFG1817	mbtA [Mycobacterium tuberculosis H37Rv]	565	24.85	511	7.00E-32	126
549473723	YP_008607372.1	LuxR family transcriptional regulator	210	580256	580888	+	VFG1824	hypothetical protein Rv3133c [Mycobacterium tuberculosis H37Rv]	217	39.81	206	3.00E-47	154
549474279	YP_008607938.1	LuxR family transcriptional regulator	211	1117019	1117654	+	VFG1824	hypothetical protein Rv3133c [Mycobacterium tuberculosis H37Rv]	217	39.41	203	1.00E-48	158
549474426	YP_008608085.1	LuxR family transcriptional regulator	214	1250465	1251109	+	VFG1824	hypothetical protein Rv3133c [Mycobacterium tuberculosis H37Rv]	217	37.86	206	1.00E-48	158
549474490	YP_008608149.1	LuxR family transcriptional regulator	210	1315062	1315694	+	VFG1824	hypothetical protein Rv3133c [Mycobacterium tuberculosis H37Rv]	217	31.55	206	2.00E-34	121
549475674	YP_008609346.1	transcriptional regulator	198	2481225	2481821	-	VFG1824	hypothetical protein Rv3133c [Mycobacterium tuberculosis H37Rv]	217	31.31	214	8.00E-27	100
549477362	YP_008611057.1	chemotaxis protein CheY	227	4059944	4060627	-	VFG1824	hypothetical protein Rv3133c [Mycobacterium tuberculosis H37Rv]	217	33.49	215	1.00E-37	130
549473629	YP_008607277.1	hypothetical protein	373	489517	490638	+	VFG1825	hypothetical protein Rv3132c [Mycobacterium tuberculosis H37Rv]	578	25.23	218	6.00E-12	63.2
549473722	YP_008607371.1	histidine kinase	347	579220	580263	+	VFG1825	hypothetical protein Rv3132c [Mycobacterium tuberculosis H37Rv]	578	27.88	208	5.00E-13	66.6
549474278	YP_008607937.1	sensor histidine kinase	372	1115889	1117007	+	VFG1825	hypothetical protein Rv3132c [Mycobacterium tuberculosis H37Rv]	578	27.27	264	1.00E-16	77.8
549474425	YP_008608084.1	histidine kinase	345	1249426	1250463	+	VFG1825	hypothetical protein Rv3132c [Mycobacterium tuberculosis H37Rv]	578	27.36	212	2.00E-12	64.3
549475675	YP_008609347.1	sensor histidine kinase	378	2481818	2482954	-	VFG1825	hypothetical protein Rv3132c [Mycobacterium tuberculosis H37Rv]	578	31.96	194	5.00E-18	82
549476744	YP_008610433.1	GTP pyrophosphokinase	731	3440163	3442358	-	VFG1826	relA [Mycobacterium tuberculosis H37Rv]	790	43.19	720	0	646
549477343	YP_008611038.1	hypothetical protein	735	4042418	4044625	-	VFG1854	flagellin [Legionella pneumophila Philadelphia 1]	475	50.7	142	1.00E-36	141
549477344	YP_008611039.1	flagellin	395	4045702	4046889	-	VFG1854	flagellin [Legionella pneumophila Philadelphia 1]	475	56.2	137	7.00E-43	154
549473611	YP_008607259.1	peptide ABC transporter permease	497	472085	473578	-	VFG1857	IraAB [Legionella pneumophila Philadelphia 1]	502	23.48	511	2.00E-33	129
549476627	YP_008610312.1	superoxide dismutase	202	3324045	3324653	-	VFG1867	superoxide dismutase [Legionella pneumophila Philadelphia 1]	196	49.49	196	1.00E-66	203
549476591	YP_008610276.1	competence protein ComG	343	3291718	3292749	-	VFG1881	(type IV) pilus assembly protein PilC [Legionella pneumophila Philadelphia 1]	406	23.3	352	1.00E-14	71.2
549476795	YP_008610484.1	prepilin peptidase	247	3486219	3486962	-	VFG1882	type 4 (IV) prepilin-like protein leader peptide processing enzyme PilD [Legionella pneumophila Philadelphia 1]	289	32.62	279	3.00E-37	132
549477345	YP_008611040.1	carbon storage regulator	77	4047032	4047265	-	VFG1886	carbon storage regulator RsmA [Legionella pneumophila Philadelphia 1]	78	53.7	54	6.00E-16	64.7
549473530	YP_008607177.1	integral membrane sensor signal transduction histidine kinase	461	404534	405919	+	VFG1888	sensory box histidine kinase/response regulator [Legionella pneumophila Philadelphia 1]	910	32.23	242	7.00E-25	104
549474025	YP_008607680.1	histidine kinase	361	850850	851935	+	VFG1888	sensory box histidine kinase/response regulator [Legionella pneumophila Philadelphia 1]	910	25.5	349	2.00E-24	101
549474242	YP_008607900.1	hypothetical protein	420	1069182	1070444	-	VFG1888	sensory box histidine kinase/response regulator [Legionella pneumophila Philadelphia 1]	910	29.11	237	4.00E-13	67.8
549474248	YP_008607906.1	hypothetical protein	956	1078573	1081443	+	VFG1888	sensory box histidine kinase/response regulator [Legionella pneumophila Philadelphia 1]	910	38.26	230	4.00E-35	140
549474323	YP_008607982.1	sensor histidine kinase	466	1156737	1158137	+	VFG1888	sensory box histidine kinase/response regulator [Legionella pneumophila Philadelphia 1]	910	29.03	341	7.00E-27	110
549475134	YP_008608801.1	histidine kinase	376	1961896	1963026	+	VFG1888	sensory box histidine kinase/response regulator [Legionella pneumophila Philadelphia 1]	910	25.45	220	1.00E-18	84
549476020	YP_008609694.1	histidine kinase	421	2758780	2760045	+	VFG1888	sensory box histidine kinase/response regulator [Legionella pneumophila Philadelphia 1]	910	24.21	347	3.00E-18	84
549476151	YP_008609828.1	histidine kinase	933	2887385	2890186	-	VFG1888	sensory box histidine kinase/response regulator [Legionella pneumophila Philadelphia 1]	910	34.68	372	3.00E-44	169
549476198	YP_008609877.1	sensor histidine kinase	463	2939399	2940790	-	VFG1888	sensory box histidine kinase/response regulator [Legionella pneumophila Philadelphia 1]	910	30.77	234	3.00E-32	127
549476891	YP_008610581.1	alkaline phosphatase	587	3597631	3599394	-	VFG1888	sensory box histidine kinase/response regulator [Legionella pneumophila Philadelphia 1]	910	34.17	240	2.00E-33	132
549477882	YP_008611584.1	sensor histidine kinase	453	4665329	4666690	+	VFG1888	sensory box histidine kinase/response regulator [Legionella pneumophila Philadelphia 1]	910	26.83	328	7.00E-27	110
549473630	YP_008607278.1	transcriptional regulator	201	490654	491259	+	VFG1889	response regulator GacA [Legionella pneumophila Philadelphia 1]	225	31.82	198	1.00E-29	108
549477713	YP_008611412.1	chemotaxis protein CheY	124	4506282	4506656	-	VFG1889	response regulator GacA [Legionella pneumophila Philadelphia 1]	225	39.32	117	3.00E-18	74.7
549477811	YP_008611510.1	hypothetical protein	525	4600823	4602400	-	VFG1889	response regulator GacA [Legionella pneumophila Philadelphia 1]	225	32.5	120	3.00E-18	80.9
549474971	YP_008608633.1	flagellar motor switch protein FliM	332	1794602	1795600	+	VFG1893	flagellar motor switch protein [Campylobacter jejuni NCTC 11168]	359	37.8	328	1.00E-69	221
549474924	YP_008608586.1	cell division protein FtsY	329	1748440	1749429	+	VFG1895	flagellar biosynthesis protein [Campylobacter jejuni NCTC 11168]	484	26.24	202	1.00E-14	70.9
549474978	YP_008608640.1	flagellar biosynthesis protein FlhB	360	1799646	1800728	+	VFG1900	flagellar biosynthetic protein [Campylobacter jejuni NCTC 11168]	362	43.14	350	1.00E-92	282
549473781	YP_008607431.1	flagellar motor protein MotA	264	628518	629312	+	VFG1902	putative flagellar motor proton channel [Campylobacter jejuni NCTC 11168]	258	34.92	252	3.00E-43	147
549474975	YP_008608637.1	flagellar biosynthesis protein FliP	221	1797915	1798580	+	VFG1915	flagellar biosynthesis protein [Campylobacter jejuni NCTC 11168]	244	55.92	211	6.00E-84	250
549474977	YP_008608639.1	flagellar biosynthesis protein FliR	260	1798867	1799649	+	VFG1918	flagellar biosynthetic protein [Campylobacter jejuni NCTC 11168]	255	30.17	242	3.00E-34	123
549474185	YP_008607843.1	enoyl-ACP reductase	261	1006052	1006837	+	VFG1922	putative oxidoreductase (flagellin modification) [Campylobacter jejuni NCTC 11168]	256	28.57	266	7.00E-22	89
549476615	YP_008610300.1	hypothetical protein	771	3311223	3313538	-	VFG1960	putative sugar transferase [Campylobacter jejuni NCTC 11168]	1031	26.09	299	8.00E-15	75.1
549473345	YP_008606987.1	histidinol phosphate aminotransferase	357	220361	221434	+	VFG1964	putative aminotransferase [Campylobacter jejuni NCTC 11168]	390	26.17	321	8.00E-24	97.4
549476357	YP_008610038.1	aspartate aminotransferase	396	3085558	3086748	-	VFG1964	putative aminotransferase [Campylobacter jejuni NCTC 11168]	390	25.38	260	4.00E-12	63.5
549475315	YP_008608984.1	hypothetical protein	363	2151081	2152172	+	VFG1965	putative aminotransferase [Campylobacter jejuni NCTC 11168]	367	27.17	368	4.00E-30	116
549476388	YP_008610069.1	histidinol-phosphate aminotransferase	366	3111723	3112823	-	VFG1965	putative aminotransferase [Campylobacter jejuni NCTC 11168]	367	27.72	368	4.00E-28	110
549476521	YP_008610206.1	ABC transporter substrate-binding protein	267	3227339	3228142	-	VFG1983	probable ABC-type amino-acid transporter periplasmic solute-binding protein [Campylobacter jejuni NCTC 11168]	259	26.49	268	3.00E-16	72.4
549478059	YP_008611763.1	ABC transporter substrate-binding protein	279	4805782	4806621	-	VFG1983	probable ABC-type amino-acid transporter periplasmic solute-binding protein [Campylobacter jejuni NCTC 11168]	259	26.83	246	3.00E-13	64.3
549475273	YP_008608941.1	adenylylsulfate kinase	204	2104104	2104718	-	VFG1988	possible adenylylsulfate kinase [Campylobacter jejuni NCTC 11168]	170	35.15	165	1.00E-27	101
549475656	YP_008609328.1	adenylylsulfate kinase	200	2465218	2465820	-	VFG1988	possible adenylylsulfate kinase [Campylobacter jejuni NCTC 11168]	170	30.06	163	1.00E-19	80.1
549474959	YP_008608621.1	flagellar M-ring protein FliF	528	1784910	1786496	+	VFG2006	flagellar basal body M-ring protein FliF [Legionella pneumophila Philadelphia 1]	544	23.29	554	3.00E-35	135
549473782	YP_008607432.1	flagellar motor protein MotB	269	629305	630114	+	VFG2008	flagellar motor protein MotB [Legionella pneumophila Philadelphia 1]	273	30.3	264	2.00E-30	114
549476956	YP_008610647.1	flagellar motor protein MotS	246	3669593	3670333	-	VFG2008	flagellar motor protein MotB [Legionella pneumophila Philadelphia 1]	273	32.79	244	1.00E-36	129
549476957	YP_008610648.1	flagellar motor protein MotP	272	3670323	3671141	-	VFG2009	flagellar motor protein MotA [Legionella pneumophila Philadelphia 1]	257	33.46	260	6.00E-46	154
549474980	YP_008608642.1	flagellar biosynthesis regulator FlhF	381	1803004	1804149	+	VFG2011	flagellar GTP-binding protein FlhF [Legionella pneumophila Philadelphia 1]	386	31.06	367	1.00E-36	135
549475357	YP_008609026.1	hypothetical protein	378	2190278	2191414	-	VFG2045	virulence sensor protein [Bordetella pertussis Tohama I]	1238	27.13	258	3.00E-19	86.3
549475924	YP_008609598.1	histidine kinase	491	2683505	2684980	-	VFG2045	virulence sensor protein [Bordetella pertussis Tohama I]	1238	25.67	261	2.00E-15	75.9
549476592	YP_008610277.1	competence protein ComG	355	3292736	3293803	-	VFG2048	putative type II secretion protein [Shigella dysenteriae Sd197]	497	42.51	287	2.00E-66	217
549474907	YP_008608569.1	serine/threonine protein kinase	658	1730662	1732638	+	VFG2060	serine/threonine protein kinase PpkA [Pseudomonas aeruginosa PAO1]	1032	40.08	242	7.00E-52	190
549474906	YP_008608568.1	protein phosphatase	253	1729907	1730668	+	VFG2061	probable phosphoprotein phosphatase [Pseudomonas aeruginosa PAO1]	242	33.33	243	2.00E-28	107
549476162	YP_008609839.1	hypothetical protein	274	2903065	2903889	+	VFG2112	VipF, N-terminal acetyltransferase, GNAT family [Legionella pneumophila Philadelphia 1]	286	27.82	284	1.00E-25	100
549474962	YP_008608624.1	ATP synthase	438	1788276	1789592	+	VFG2144	type III secretion system ATPase [Chlamydia trachomatis D/UW-3/CX]	442	51.52	429	2.00E-147	428
549476296	YP_008609976.1	hypothetical protein	307	3031249	3032172	-	VFG2157	lipoprotein [Listeria monocytogenes (serovar 1/2a) EGD-e]	310	31.63	313	3.00E-53	176
549474436	YP_008608095.1	lipoate-protein ligase A	330	1260352	1261344	+	VFG2158	lipoate protein ligase [Listeria monocytogenes (serovar 1/2a) EGD-e]	331	65.66	332	2.00E-162	457
549476575	YP_008610260.1	octanoyltransferase	278	3280210	3281046	-	VFG2158	lipoate protein ligase [Listeria monocytogenes (serovar 1/2a) EGD-e]	331	28.16	277	6.00E-21	87
549474893	YP_008608555.1	hypothetical protein	570	1715076	1716788	-	VFG2160	fibronectin-binding protein [Listeria monocytogenes (serovar 1/2a) EGD-e]	570	54.29	571	0	630
549474881	YP_008608543.1	peptidase A8	162	1701736	1702224	+	VFG2161	signal peptidase II [Listeria monocytogenes (serovar 1/2a) EGD-e]	154	60.93	151	3.00E-57	176
549475140	YP_008608807.1	hypothetical protein	329	1967137	1968126	-	VFG2162	bile salt hydrolase [Listeria monocytogenes (serovar 1/2a) EGD-e]	325	27.16	324	7.00E-24	96.3
549473114	YP_008609471.1	XRE family transcriptional regulator	68	2577575	2577781	-	VFG2168	transcriptional regulator, Cro/CI family [Enterococcus faecalis V583]	66	44.44	63	2.00E-13	57.4
549473137	YP_008611139.1	XRE family transcriptional regulator	66	4177047	4177247	-	VFG2168	transcriptional regulator, Cro/CI family [Enterococcus faecalis V583]	66	51.52	66	3.00E-18	70.1
549473299	YP_008606941.1	Cro/CI family transcriptional regulator	70	181911	182123	+	VFG2168	transcriptional regulator, Cro/CI family [Enterococcus faecalis V583]	66	46.27	67	1.00E-13	58.2

549473544	YP_008607191.1	DNA-binding protein	69	415468	415677	+	VFG2168	transcriptional regulator, Cro/CI family [Enterococcus faecalis V583]	66	45.16	62	3.00E-14	59.7
549475221	YP_008608889.1	DNA-binding protein	71	2054925	2055140	+	VFG2168	transcriptional regulator, Cro/CI family [Enterococcus faecalis V583]	66	53.97	63	6.00E-16	64.3
549475866	YP_008609540.1	XRE family transcriptional regulator	67	2632424	2632627	-	VFG2168	transcriptional regulator, Cro/CI family [Enterococcus faecalis V583]	66	42.42	66	3.00E-13	57
549475416	YP_008609085.1	serine protease	320	2256353	2257315	-	VFG2177	cytolysin activator [Enterococcus faecalis MMH594]	412	30.71	267	3.00E-18	81.3
549475535	YP_008609205.1	peptidase S8	447	2358358	2359701	-	VFG2177	cytolysin activator [Enterococcus faecalis MMH594]	412	26.38	307	5.00E-18	82.4
549473360	YP_008607002.1	LacI family transcriptional regulator	339	230489	231508	+	VFG2197	sugar-binding transcriptional regulator, LacI family [Enterococcus faecalis V583]	336	28.57	315	1.00E-38	139
549473491	YP_008607138.1	LacI family transcriptional regulator	329	363264	364253	+	VFG2197	sugar-binding transcriptional regulator, LacI family [Enterococcus faecalis V583]	336	24.44	315	4.00E-11	59.7
549474082	YP_008607738.1	LacI family transcription regulator	331	910640	911635	+	VFG2197	sugar-binding transcriptional regulator, LacI family [Enterococcus faecalis V583]	336	28.28	343	3.00E-28	109
549474260	YP_008607918.1	LacI family transcription regulator	323	1095340	1096311	+	VFG2197	sugar-binding transcriptional regulator, LacI family [Enterococcus faecalis V583]	336	24.5	347	9.00E-22	90.5
549474508	YP_008608167.1	LacI family transcription regulator	341	1337432	1338457	+	VFG2197	sugar-binding transcriptional regulator, LacI family [Enterococcus faecalis V583]	336	39.13	345	1.00E-74	234
549475168	YP_008608835.1	LacI family transcriptional regulator	331	2000328	2001323	+	VFG2197	sugar-binding transcriptional regulator, LacI family [Enterococcus faecalis V583]	336	26.1	341	3.00E-31	118
549475832	YP_008609506.1	transcriptional regulator	338	2604115	2605131	-	VFG2197	sugar-binding transcriptional regulator, LacI family [Enterococcus faecalis V583]	336	23.21	349	5.00E-21	88.6
549476958	YP_008610649.1	catabolite control protein A	332	3671248	3672246	-	VFG2197	sugar-binding transcriptional regulator, LacI family [Enterococcus faecalis V583]	336	27.95	347	2.00E-37	135
549477418	YP_008611113.1	hypothetical protein	340	4143498	4144520	-	VFG2197	sugar-binding transcriptional regulator, LacI family [Enterococcus faecalis V583]	336	29.02	348	6.00E-39	139
549477436	YP_008611131.1	LacI family transcription regulator	336	4164579	4165589	-	VFG2197	sugar-binding transcriptional regulator, LacI family [Enterococcus faecalis V583]	336	26.07	326	3.00E-23	94.7
549477529	YP_008611225.1	transcriptional regulator	340	4276490	4277512	-	VFG2197	sugar-binding transcriptional regulator, LacI family [Enterococcus faecalis V583]	336	29.78	319	8.00E-33	122
549477463	YP_008611159.1	spore coat protein	410	4201055	4202287	-	VFG2226	perosamine synthetase [Brucella melitensis 16M]	380	33.78	296	3.00E-42	151
549477602	YP_008611298.1	aminotransferase DegT	376	4367430	4368560	-	VFG2226	perosamine synthetase [Brucella melitensis 16M]	380	34.25	365	5.00E-68	219
549474903	YP_008608565.1	methionyl-tRNA formyltransferase	318	1726522	1727478	+	VFG2230	GDP-mannose 4,6-dehydratase / GDP-4-amino-4,6-dideoxy-D-mannose formyltransferase [Brucella melitensis 16M]	259	32.94	170	3.00E-19	82.4
549474988	YP_008608650.1	RNA polymerase sigma factor SigD	253	1810063	1810824	+	VFG2319	RNA polymerase sigma factor for flagellar operon [Yersinia enterocolitica 8081]	240	35.68	227	7.00E-46	153
549477341	YP_008611036.1	flagellar hook protein	698	4039755	4041851	-	VFG2324	flagellar hook-associated protein 2 [Yersinia enterocolitica 8081]	466	24.36	312	8.00E-13	67.4
549477340	YP_008611035.1	flagellar biosynthesis protein FliS	133	4039311	4039712	-	VFG2325	flagellar protein FliS [Yersinia enterocolitica 8081]	132	36.36	110	7.00E-18	72.8
549474957	YP_008608619.1	flagellar basal body rod protein FlgC	149	1784061	1784510	+	VFG2350	flagellar basal-body rod protein FlgC [Yersinia enterocolitica 8081]	134	44.3	149	4.00E-36	121
549474956	YP_008608618.1	flagellar basal body rod protein FlgB	132	1783660	1784058	+	VFG2351	flagellar basal-body rod protein FlgB [Yersinia enterocolitica 8081]	137	28	125	2.00E-15	66.2
549473493	YP_008607140.1	UDP-glucose 4-epimerase	329	365465	366454	+	VFG2361	UDP-glucose 4-epimerase [Yersinia enterocolitica 8081]	336	44.67	338	5.00E-91	276
549477581	YP_008611277.1	UDP-glucose 4-epimerase	295	4344089	4344976	-	VFG2361	UDP-glucose 4-epimerase [Yersinia enterocolitica 8081]	336	53.56	295	1.00E-110	324
549477587	YP_008611283.1	UDP-glucose 4-epimerase	334	4349577	4350581	-	VFG2361	UDP-glucose 4-epimerase [Yersinia enterocolitica 8081]	336	53.35	328	2.00E-127	369
549477591	YP_008611287.1	UDP-glucose 4-epimerase	336	4355041	4356051	-	VFG2361	UDP-glucose 4-epimerase [Yersinia enterocolitica 8081]	336	53.35	328	8.00E-130	375
549473297	YP_008606939.1	phosphoglucosamine mutase	449	177927	179276	+	VFG2362	phosphomannomutase [Yersinia enterocolitica 8081]	456	24.34	452	1.00E-31	122
549477599	YP_008611295.1	hypothetical protein	393	4364200	4365381	-	VFG2366	putative galactosyltransferase [Yersinia enterocolitica 8081]	358	28.5	207	4.00E-14	69.3
549475279	YP_008608947.1	hypothetical protein	234	2110569	2111273	-	VFG2368	putative glycosyltransferase [Yersinia enterocolitica 8081]	288	30.05	203	7.00E-23	91.7
549475291	YP_008608959.1	hypothetical protein	246	2125523	2126263	+	VFG2368	putative glycosyltransferase [Yersinia enterocolitica 8081]	288	29.5	200	3.00E-24	96.3
549475296	YP_008608964.1	glycosyl transferase	247	2131215	2131958	+	VFG2368	putative glycosyltransferase [Yersinia enterocolitica 8081]	288	30.22	225	2.00E-21	87.8
549475281	YP_008608949.1	spore coat protein	152	2112705	2113163	-	VFG2375	putative epimerase [Yersinia enterocolitica 8081]	174	27.59	145	3.00E-12	58.2
549475066	YP_008608731.1	stage V sporulation protein K	324	1893626	1894600	+	VFG2380	hypothetical protein [Mycobacterium tuberculosis H37Rv]	573	36.69	278	1.00E-50	175
549475351	YP_008609020.1	stage V sporulation protein K	787	2185896	2188259	+	VFG2380	hypothetical protein [Mycobacterium tuberculosis H37Rv]	573	37.81	283	7.00E-47	172
549477373	YP_008611068.1	alkaline serine protease	1170	4072858	4076370	-	VFG2391	MEMBRANE-ANCHORED MYCOSIN MYCP1 (SERINE PROTEASE) (SUBTILISIN-LIKE PROTEASE) (SUBTILASE-LIKE) (MYC(	446	29.11	371	1.00E-16	80.5
549475028	YP_008608692.1	cell division protein FtsK	782	1850742	1853090	+	VFG2409	hypothetical protein [Staphylococcus aureus MW2]	1479	33.19	232	7.00E-22	98.2
549476968	YP_008610659.1	hypothetical protein	1106	3682990	3686310	-	VFG2409	hypothetical protein [Staphylococcus aureus MW2]	1479	33.18	220	3.00E-21	96.7
549474712	YP_008608372.1	2', 3'-cyclic nucleotide 2'-phosphodiesterase	532	1544911	1546509	+	VFG2420	Adenosine synthase A [Staphylococcus aureus MW2]	772	23.85	524	2.00E-26	110
549475523	YP_008609193.1	hypothetical protein	371	2348955	2350070	+	VFG2420	Adenosine synthase A [Staphylococcus aureus MW2]	772	23.51	285	2.00E-13	68.2
549477609	YP_008611305.1	hypothetical protein	870	4378372	4380984	-	VFG2420	Adenosine synthase A [Staphylococcus aureus MW2]	772	29.45	489	1.00E-48	181
549477630	YP_008611326.1	2', 3'-cyclic nucleotide 2'-phosphodiesterase	1617	4420188	4425041	-	VFG2420	Adenosine synthase A [Staphylococcus aureus MW2]	772	27.59	482	3.00E-31	129
549474968	YP_008608630.1	flagellar basal body rod protein FlgG	270	1793057	1793869	+	VFG2513	flagellar basal body rod protein FlgG [Burkholderia pseudomallei K96243]	262	40.22	271	5.00E-49	162
549477646	YP_008611343.1	flagellar basal body rod protein subunit C	279	4441837	4442676	-	VFG2513	flagellar basal body rod protein FlgG [Burkholderia pseudomallei K96243]	262	30.6	281	2.00E-26	102
549474979	YP_008608641.1	flagellar biosynthesis protein FlhA	678	1800971	1803007	+	VFG2522	flagellar biosynthesis protein FlhA [Burkholderia pseudomallei K96243]	700	42.3	688	2.00E-180	531
549474946	YP_008608608.1	hypothetical protein	99	1771514	1771813	+	VFG2523	flagellar biosynthesis protein FlhB [Burkholderia pseudomallei K96243]	405	46.84	79	9.00E-14	63.2
549474247	YP_008607905.1	histidine kinase	123	1078215	1078586	+	VFG2525	chemotaxis protein CheY [Burkholderia pseudomallei K96243]	142	33.9	118	9.00E-18	72.4
549474973	YP_008608635.1	chemotaxis protein CheY	120	1796838	1797200	+	VFG2525	chemotaxis protein CheY [Burkholderia pseudomallei K96243]	142	36.67	120	1.00E-19	77.4
549474982	YP_008608644.1	chemotaxis protein CheY	363	1805015	1806106	+	VFG2526	chemotaxis-specific methylesterase [Burkholderia pseudomallei K96243]	367	43.02	358	6.00E-90	275
549476537	YP_008610222.1	chemotaxis protein CheY	262	3247828	3248616	-	VFG2526	chemotaxis-specific methylesterase [Burkholderia pseudomallei K96243]	367	44.55	110	2.00E-17	77.4
549474986	YP_008608648.1	chemotaxis protein CheD	165	1809301	1809798	+	VFG2527	chemoreceptor glutamine deamidase CheD [Burkholderia pseudomallei K96243]	234	31.16	138	1.00E-16	71.2
549474651	YP_008608310.1	histidine kinase	1175	1470572	1474099	+	VFG2528	chemotaxis protein methyltransferase [Burkholderia pseudomallei K96243]	315	26.74	273	1.00E-18	85.1
549476150	YP_008609827.1	chemotaxis protein R	278	2886529	2887365	-	VFG2528	chemotaxis protein methyltransferase [Burkholderia pseudomallei K96243]	315	30.16	189	1.00E-17	77.8
549476392	YP_008610073.1	chemotaxis protein CheR	257	3115811	3116584	-	VFG2528	chemotaxis protein methyltransferase [Burkholderia pseudomallei K96243]	315	33.19	238	1.00E-33	122
549476048	YP_008609722.1	hypothetical protein	565	2782243	2783940	-	VFG2529	methyl-accepting chemotaxis protein I [Burkholderia pseudomallei K96243]	673	26.67	345	2.00E-20	91.7
549477332	YP_008611027.1	methyl-accepting chemotaxis protein	552	4033410	4035068	+	VFG2529	methyl-accepting chemotaxis protein I [Burkholderia pseudomallei K96243]	673	33.47	242	2.00E-21	94.7
549474984	YP_008608646.1	chemotaxis protein CheW	154	1808195	1808659	+	VFG2530	chemotaxis protein CheW [Burkholderia pseudomallei K96243]	175	39.58	144	1.00E-32	113
549474983	YP_008608645.1	chemotaxis protein CheA	674	1806127	1808151	+	VFG2531	chemotaxis two-component sensor kinase CheA [Burkholderia pseudomallei K96243]	747	42.71	398	2.00E-98	319
549475334	YP_008609003.1	2-amino-3-ketobutyrate CoA ligase	411	2172390	2173625	+	VFG2546	acyl-CoA transferase [Burkholderia pseudomallei K96243]	439	33.24	361	2.00E-58	196
549473829	YP_008607481.1	NADPH	337	674633	675646	+	VFG2548	capsular polysaccharide biosynthesis fatty acid synthase [Burkholderia pseudomallei K96243]	2546	39.86	138	1.00E-14	71.6
549474542	YP_008608201.1	3-oxoacyl-ACP synthase	413	1369001	1370242	+	VFG2548	capsular polysaccharide biosynthesis fatty acid synthase [Burkholderia pseudomallei K96243]	2546	28.05	385	2.00E-31	125
549474601	YP_008608260.1	alcohol dehydrogenase	323	1425024	1425995	+	VFG2548	capsular polysaccharide biosynthesis fatty acid synthase [Burkholderia pseudomallei K96243]	2546	31.72	268	4.00E-25	104
549474918	YP_008608580.1	malonyl CoA-ACP transacylase	315	1741756	1742703	+	VFG2548	capsular polysaccharide biosynthesis fatty acid synthase [Burkholderia pseudomallei K96243]	2546	28.52	298	2.00E-24	102
549474176	YP_008607834.1	short-chain dehydrogenase	224	1001001	1001675	-	VFG2550	capsular polysaccharide biosynthesis dehydrogenase/reductase [Burkholderia pseudomallei K96243]	262	30.14	219	3.00E-16	72
549476500	YP_008610184.1	oxidoreductase	265	3207160	3207957	-	VFG2550	capsular polysaccharide biosynthesis dehydrogenase/reductase [Burkholderia pseudomallei K96243]	262	28.79	257	2.00E-17	76.3
549473828	YP_008607479.1	hypothetical protein	180	673417	673959	+	VFG2552	D-glycero-d-manno-heptose 1,7-bisphosphate phosphatase [Burkholderia pseudomallei K96243]	189	35.42	144	5.00E-14	63.9
549474368	YP_008608027.1	histidinol phosphatase	173	1200730	1201251	+	VFG2552	D-glycero-d-manno-heptose 1,7-bisphosphate phosphatase [Burkholderia pseudomallei K96243]	189	35.71	154	4.00E-26	97.4
549475278	YP_008608946.1	hypothetical protein	312	2109634	2110572	+	VFG2556	GDP sugar epimerase/dehydratase [Burkholderia pseudomallei K96243]	337	29.02	348	4.00E-33	123

Table S3: List of unique genes for virulence factor encoded in the genome of *Bacillus* sp. NRRL B-14911.

NRRLB14911 GI ID	NRRL B-14911 protein ID	NRRL B-14911 protein description	Length (aa)	Start position (genome)	End position (genome)	Strand (genome)	VFDB protein ID	VFDB protein description	VF length (aa)	% positive	Aligned length	E-value	Bit score
549474315	YP_0086079[illegible]	ribonuclease yfkH	286	1149702	1150562	+	VFG0028	serum resistance protein [Bordetella pertussis Tohama I]	296	25.85	294	6.00E-15	70.1
549477814	YP_0086115[illegible]	AraC family transcriptional regulator	298	4605066	4605962	+	VFG0167	transcriptional regulator PchR [Pseudomonas aeruginosa PAO1]	296	32.73	110	9.00E-12	61.2
549475283	YP_0086089[illegible]	spore coat protein	318	2114001	2114957	-	VFG0314	UDP-glucose 4-epimerase [Helicobacter pylori 26695]	344	25.36	351	1.00E-21	90.1
549475931	YP_0086096[illegible]	hypothetical protein	277	2690664	2691497	-	VFG0481	putative hydrolase or acyltransferase [Salmonella enterica (serovar typh	297	30.77	104	3.00E-11	59.3
549473873	YP_0086075[illegible]	hypothetical protein	443	725814	727145	+	VFG0564	ATP binding protein [Salmonella enterica (serovar typhimurium) LT2]	396	41	100	6.00E-15	72.8
549473529	YP_0086071[illegible]	regulator	224	403870	404544	+	VFG0596	Copper resistance; transcriptional regulatory protein [Salmonella enteric	248	38.84	224	9.00E-47	155
549475001	YP_0086086[illegible]	transposase	370	1821987	1823099	+	VFG0628	orf, partial conserved hypothetical protein [Shigella flexneri (serotype 2a	121	49.02	102	1.00E-21	86.7
549478071	YP_0086117[illegible]	ABC transporter permease	343	4818952	4819983	+	VFG0928	Ferric enterobactin transport system permease protein fepG [Escherichi	330	34.98	303	1.00E-43	152
549477536	YP_0086112[illegible]	N-acetylmannosamine-6-phosphate 2-epimerase	227	4283874	4284557	-	VFG1114	conserved hypothetical protein [Vibrio cholerae N16961]	240	35.65	216	5.00E-35	124
549477645	YP_0086113[illegible]	flagellar basal body rod protein FlgG	274	4440963	4441787	-	VFG1240	flagellar basal-body rod protein FlgG [Pseudomonas aeruginosa PAO1]	261	35.59	281	2.00E-33	121
549475275	YP_0086089[illegible]	UDP-N-acetylglucosamine 2-epimerase	363	2106740	2107831	+	VFG1303	capsular polysaccharide synthesis enzyme Cap8G [Staphylococcus aureu	374	43.89	360	4.00E-104	312
549477596	YP_0086112[illegible]	hypothetical protein	420	4360627	4361889	-	VFG1307	capsular polysaccharide synthesis enzyme Cap8K [Staphylococcus aureu	412	25.84	356	1.00E-17	81.3
549477541	YP_0086112[illegible]	hypothetical protein	451	4291397	4292752	-	VFG1442	invasion protein IbeA [Escherichia coli]	456	24.77	440	3.00E-26	107
549476615	YP_0086103[illegible]	hypothetical protein	771	3311223	3313538	-	VFG1960	putative sugar transferase [Campylobacter jejuni NCTC 11168]	1031	26.09	299	8.00E-15	75.1
549473345	YP_0086069[illegible]	histidinol phosphate aminotransferase	357	220361	221434	+	VFG1964	putative aminotransferase [Campylobacter jejuni NCTC 11168]	390	26.17	321	8.00E-24	97.4
549474959	YP_0086086[illegible]	flagellar M-ring protein FliF	528	1784910	1786496	+	VFG2006	flagellar basal body M-ring protein FliF [Legionella pneumophila Philadel	544	23.29	554	3.00E-35	135
549477599	YP_0086112[illegible]	hypothetical protein	393	4364200	4365381	-	VFG2366	putative galactosyltransferase [Yersinia enterocolitica 8081]	358	28.5	207	4.00E-14	69.3
549473828	YP_0086074[illegible]	hypothetical protein	180	673417	673959	+	VFG2552	D-glycero-d-manno-heptose 1,7-bisphosphate phosphatase [Burkholder	189	35.42	144	5.00E-14	63.9

Table S4: List of genes for virulence factor encoded in the genome of *Bacilllus* sp. NRRL B-14911 that are in common with pathogenic Bacilli.

NRRLB14911 GI ID	NRRL B-14911 protein ID	NRRL B-14911 protein description	Length (aa)	Start position (genome)	End position (genome)	Strand (genome)	VFDB protein ID	VFDB protein description	VF length (aa)	% positive	Aligned length	E-value	Bit score
**NRRLB14911**	**NRRLB14911**	**NRRLB14911 protein Description**	**Length (aa)**	**Start Position**	**End Position (**	**Strand (genoı**	**VFDB protein**	**VFDB protein Description**	**VF length (aa**	**% indentity**	**Aligned lengt**	**E-value**	**Bit score**
549475378	YP_00860904	glycosyl transferase family 1	331	2215049	2216044	+	VFG0031	putative glycosyl transferase [Bordetella pertussis Tohama I]	390	29.2	137	3.00E-11	60.1
549475286	YP_00860895	aminotransferase DegT	373	2116523	2117644	-	VFG0033	lipopolysaccharide biosynthesis protein [Bordetella pertussis Tohama I]	410	24.75	396	3.00E-24	99.4
549477593	YP_00861128	aminotransferase DegT	376	4357186	4358316	-	VFG0036	lipopolysaccharide biosynthesis protein [Bordetella pertussis Tohama I]	366	41.21	381	4.00E-90	276
549474760	YP_00860842	2,3,4,5-tetrahydropyridine-2,6-carboxylate N-succinyltransferase	236	1587357	1588067	+	VFG0037	probable acetyltransferase [Bordetella pertussis Tohama I]	191	30.52	154	6.00E-14	64.3
549474218	YP_00860787	NADH-dependent dehydrogenase	342	1047655	1048683	+	VFG0038	probable oxidoreductase [Bordetella pertussis Tohama I]	350	26.36	349	4.00E-28	109
549474625	YP_00860828	peptidase	309	1448499	1449428	+	VFG0070	P60 extracellular protein, invasion associated protein Iap [Listeria monocytogenes (serovar 1/2a) EGD-e]	482	35.79	95	4.00E-17	78.2
549477271	YP_00861096	ATP-dependent Clp protease proteolytic subunit	201	3971231	3971836	+	VFG0077	ATP-dependent Clp protease proteolytic subunit [Listeria monocytogenes (serovar 1/2a) EGD-e]	198	77.89	190	6.00E-111	316
549473237	YP_00860686	ATP-dependent Clp protease ATP-binding protein	817	109202	111655	+	VFG0079	endopeptidase Clp ATP-binding chain C [Listeria monocytogenes (serovar 1/2a) EGD-e]	820	79.23	809	0	1313
549474716	YP_00860837	ATP-dependent Clp protease ATP-binding protein	708	1550832	1552958	-	VFG0080	ATP-dependent protease [Listeria monocytogenes (serovar 1/2a) EGD-e]	724	65.41	714	0	928
549473420	YP_00860706	1-pyrroline-5-carboxylate dehydrogenase	515	299210	300757	+	VFG0082	aldehyde dehydrogenase [Vibrio cholerae N16961]	541	31.57	472	1.00E-59	204
549476928	YP_00861061	lipid hydroperoxide peroxidase	166	3636104	3636604	-	VFG0087	tagD protein [Vibrio cholerae N16961]	164	36.94	157	3.00E-31	110
549473998	YP_00860765	hypothetical protein	402	831171	832379	-	VFG0088	toxin co-regulated pilus biosynthesis protein I [Vibrio cholerae N16961]	620	33.96	159	4.00E-15	73.6
549475146	YP_00860881	chemotaxis protein	561	1973569	1975254	+	VFG0104	accessory colonization factor AcfB [Vibrio cholerae N16961]	626	32.51	323	2.00E-35	137
549476797	YP_00861048	type II secretion system protein F	401	3487491	3488696	-	VFG0113	still frameshift type 4 fimbrial biogenesis protein PilC [Pseudomonas aeruginosa PAO1]	374	38.67	375	1.00E-85	265
549474310	YP_00860796	LuxR family transcriptional regulator	233	1142656	1143357	-	VFG0119	alginate biosynthesis regulatory protein AlgR [Pseudomonas aeruginosa PAO1]	248	24.03	233	1.00E-19	81.6
549473501	YP_00860714	membrane protein	581	373576	375321	+	VFG0120	sigma factor AlgU [Pseudomonas aeruginosa PAO1]	358	26.01	296	7.00E-22	94
549473374	YP_00860701	RNA polymerase sigma70 factor	183	245380	245931	-	VFG0121	alginate biosynthesis protein AlgZ/FimS [Pseudomonas aeruginosa PAO1]	193	24.6	187	2.00E-11	56.6
549475738	YP_00860941	nucleotide sugar dehydrogenase	435	2527240	2528547	-	VFG0122	GDP-mannose 6-dehydrogenase AlgD [Pseudomonas aeruginosa PAO1]	436	31.64	414	3.00E-60	202
549477375	YP_00861107	acetyltransferase	481	4077756	4079201	-	VFG0130	alginate o-acetyltransferase AlgI [Pseudomonas aeruginosa PAO1]	520	37.69	398	1.00E-76	249
549478111	YP_00861181	glycosyl transferase	383	4867768	4868919	-	VFG0139	B-band O-antigen polymerase [Pseudomonas aeruginosa PAO1]	373	28.8	250	4.00E-17	78.6
549473798	YP_00860744	hypothetical protein	86	644094	644354	+	VFG0168	pyochelin biosynthesis protein PchD [Pseudomonas aeruginosa PAO1]	547	41.67	72	1.00E-11	57
549477055	YP_00861074	isochorismate synthase	468	3766629	3768035	-	VFG0171	salicylate biosynthesis isochorismate synthase [Pseudomonas aeruginosa PAO1]	476	35.19	466	5.00E-83	264
549476799	YP_00861048	type II secretion system protein E	553	3489749	3491410	-	VFG0182	general secretion pathway protein E [Pseudomonas aeruginosa PAO1]	502	51.04	384	5.00E-132	395
549477616	YP_00861131	glycosyl transferase family 1	390	4390660	4391832	+	VFG0242	alpha-1,2-N-acetylglucosamine transferase [Neisseria meningitidis MC58 (serogroup B)]	354	30.07	143	2.00E-12	64.3
549477039	YP_00861073	general stress protein	145	3751262	3751699	-	VFG0307	neutrophil activating protein (napA) (bacterioferritin) [Helicobacter pylori 26695]	144	34.59	133	6.00E-22	84.3
549474832	YP_00860849	phosphopantetheine adenylyltransferase	162	1655265	1655753	+	VFG0320	lipopolysaccharide core biosynthesis protein (kdtB) [Helicobacter pylori 26695]	157	45.16	155	4.00E-44	143
549473652	YP_00860730	hypothetical protein	289	511264	512133	+	VFG0344	iron(III) ABC transporter, ATP-binding protein [Haemophilus influenzae Rd]	351	25.57	219	9.00E-19	81.6
549474469	YP_00860812	long-chain fatty acid--CoA ligase	502	1292093	1293601	+	VFG0359	yersiniabactin siderophore biosynthetic protein [Yersinia pestis CO92]	525	28.48	495	9.00E-45	162
549475762	YP_00860943	ABC transporter	609	2546361	2548190	-	VFG0366	inner membrane ABC-transporter YbtQ [Yersinia pestis CO92]	600	31.14	533	4.00E-71	240
549476124	YP_00860979	anthranilate synthase subunit I	467	2856282	2857685	-	VFG0368	putative salicylate synthetase [Yersinia pestis CO92]	434	33.73	255	2.00E-33	127
549477676	YP_00861137	F0F1 ATP synthase subunit beta	473	4469664	4471085	-	VFG0390	putative Yops secretion ATP synthase [Yersinia pestis CO92]	439	29.53	430	2.00E-44	160
549475335	YP_00860900	UDP-glucose 4-epimerase	316	2173673	2174623	+	VFG0430	Vi polysaccharide biosynthesis protein, epimerase [Salmonella enterica (serovar typhi) CT18]	348	27.63	257	7.00E-12	62
549475903	YP_00860957	transcriptional regulator	232	2663034	2663732	-	VFG0475	response regulator in two-component regulatory system with PhoQ, transcribes genes expressed under low Mg+ concentration (OmpR family) [Salmonella ent	224	28.82	229	2.00E-30	111
549474870	YP_00860853	RNA polymerase sigma 70	239	1691736	1692455	+	VFG0477	sigma S (sigma 38) factor of RNA polymerase, major sigmafactor during stationary phase [Salmonella enterica (serovar typhimurium) LT2]	330	28.91	256	1.00E-21	88.6
549474208	YP_00860786	Fur family transcriptional regulator	145	1031667	1032104	+	VFG0478	transcriptional repressor of iron-responsive genes (Fur family) (ferric uptake regulator) [Salmonella enterica (serovar typhimurium) LT2]	150	30.88	136	4.00E-17	71.6
549476901	YP_00861059	pyruvate kinase	586	3607602	3609362	-	VFG0479	pyruvate kinase I (formerly F), fructose stimulated [Salmonella enterica (serovar typhimurium) LT2]	470	52.42	475	3.00E-165	480
549477413	YP_00861110	fructokinase	318	4137103	4138059	-	VFG0483	putative regulatory protein, deoR family [Salmonella enterica (serovar typhimurium) LT2]	408	25.19	258	2.00E-11	60.8
549476421	YP_00861010	MerR family transcriptional regulator	301	3143725	3144630	-	VFG0491	putative regulatory proteins, merR family [Salmonella enterica (serovar typhimurium) LT2]	242	36.99	73	1.00E-12	62.8
549474257	YP_00860791	hypothetical protein	457	1091936	1093309	+	VFG0493	Secretion system regulator:Sensor component [Salmonella enterica (serovar typhimurium) LT2]	920	27.42	423	1.00E-31	125
549476533	YP_00861021	ATPase AAA	692	3243235	3245313	-	VFG0524	formate hydrogen-lyase transcriptional activator for fdhF, hyc and hyp operons (EBP family) [Salmonella enterica (serovar typhimurium) LT2]	692	40.91	330	2.00E-69	239
549476295	YP_00860997	manganese ABC transporter ATP-binding protein	249	3030488	3031237	-	VFG0526	Salmonella iron transporter: fur regulated [Salmonella enterica (serovar typhimurium) LT2]	273	44.12	238	2.00E-70	217
549476293	YP_00860997	iron ABC transporter	293	3028286	3029167	-	VFG0527	Salmonella iron transporter: fur regulated [Salmonella enterica (serovar typhimurium) LT2]	286	28.57	287	6.00E-34	124
549476637	YP_00861032	metal ABC transporter permease	287	3333419	3334282	-	VFG0528	Salmonella iron transporter: fur regulated [Salmonella enterica (serovar typhimurium) LT2]	282	29.12	261	3.00E-27	105
549475055	YP_00860872	DNA mismatch repair protein MutS	883	1882508	1885159	+	VFG0562	methyl-directed mismatch repair, recognize exocyclic adducts of guanosine [Salmonella enterica (serovar typhimurium) LT2]	855	38.89	882	0	610
549473818	YP_00860746	ATPase	639	663854	665773	-	VFG0574	Mg<up>2+</up> transport protein [Salmonella enterica (serovar typhimurium) LT2]	908	28.57	189	7.00E-13	68.2
549477067	YP_00861076	methyltransferase	233	3788396	3789097	-	VFG0575	Mg<up>2+</up> transport protein [Salmonella enterica (serovar typhimurium) LT2]	231	38.21	123	3.00E-18	77.4
549473138	YP_00861133	single-stranded DNA-binding protein	121	4437988	4438353	-	VFG0576	ssDNA-binding protein controls activity of RecBCD nuclease [Salmonella enterica (serovar typhimurium) LT2]	176	36.08	97	4.00E-17	71.2
549473602	YP_00860725	histidine kinase	744	462483	464717	+	VFG0584	putative diguanylate cyclase/phosphodiesterase [Salmonella enterica (serovar typhimurium) LT2]	533	32.39	247	1.00E-36	142
549473962	YP_00860761	AraC family transcriptional regulator	311	808252	809187	-	VFG0585	transcriptional activator of superoxide response regulon (AraC/XylS family) [Salmonella enterica (serovar typhimurium) LT2]	107	31	100	7.00E-12	58.9
549473574	YP_00860722	RNA-directed DNA polymerase	427	437743	439026	+	VFG0639	orf, conserved hypothetical protein [Shigella flexneri (serotype 2a) 301]	502	30.89	382	3.00E-48	171
549473099	YP_00860781	bactoprenol glucosyl transferase	323	988376	989347	+	VFG0670	bactoprenol glucosyl transferase [Shigella flexneri (serotype 2a) 301]	309	40.79	304	1.00E-78	243
549473837	YP_00860748	gamma-glutamyltranspeptidase	562	680721	682409	+	VFG0679	<gamma>-glutamyltranspeptidase [Bacillus anthracis]	466	30.65	522	2.00E-59	204
549473241	YP_00860686	2-C-methyl-D-erythritol 4-phosphate cytidylyltransferase	230	115654	116346	+	VFG0700	bifunctional; ribulose 5-phosphate reductase; CDP-ribitol pyrophosphorylase [Haemophilus influenzae 1007]	474	29.79	235	8.00E-26	101
549474352	YP_00860801	multidrug ABC transporter permease	669	1185322	1187331	+	VFG0841	hemolysin transport protein [Escherichia coli O157:H7 EDL933]	706	31.96	510	1.00E-72	248
549475901	YP_00860957	bacitracin ABC transporter ATP-binding protein	256	2661175	2661945	-	VFG0869	AatC ATB binding protein of ABC transporter [Escherichia coli 042]	209	38.04	184	3.00E-32	116
549473316	YP_00860695	hypothetical protein	185	191229	191786	-	VFG0872	Type 1 fimbriae Regulatory protein fimE [Escherichia coli CFT073]	198	34	150	8.00E-17	72
549475149	YP_00860881	ABC transporter	587	1977850	1979613	-	VFG0907	Hemolysin B [Escherichia coli CFT073]	707	28.68	551	3.00E-68	234
549473341	YP_00860698	iron ABC transporter substrate-binding protein	323	215963	216934	+	VFG0918	Putative Periplasmic binding protein [Escherichia coli CFT073]	330	27.8	259	1.00E-25	101
549474372	YP_00860803	coproporphyrinogen III oxidase	501	1203717	1205222	+	VFG0919	Putative oxygen independent coproporphyrinogen III oxidase [Escherichia coli CFT073]	445	26.47	170	2.00E-13	68.6
549475584	YP_00860925	NAD dependent epimerase/dehydratase	214	2400032	2400676	+	VFG0921	Hypothetical protein [Escherichia coli CFT073]	207	28.44	211	2.00E-12	60.1
549473924	YP_00860757	iron ABC transporter permease	318	770107	771063	+	VFG0922	Putative permease of iron compound ABC transport system [Escherichia coli CFT073]	330	28.39	310	1.00E-27	107
549474199	YP_00860785	daunorubicin ABC transporter ATP-binding protein	335	1025124	1026131	+	VFG0925	Ferric enterobactin transport ATP-binding protein fepC [Escherichia coli CFT073]	271	28.31	219	1.00E-21	89.7
549477196	YP_00861089	siderophore ABC transporter permease	333	3898153	3899154	-	VFG0926	Ferric enterobactin transport system permease protein fepD [Escherichia coli CFT073]	338	43.09	304	6.00E-64	206
549474481	YP_00860814	AMP-dependent synthetase	530	1305608	1307200	+	VFG0932	Enterobactin synthetase component E [Escherichia coli CFT073]	536	26.3	540	9.00E-36	136
549473166	YP_00860678	isochorismatase	187	27911	28474	-	VFG0933	Isochorismatase [Escherichia coli CFT073]	285	30.82	159	6.00E-14	65.1
549474092	YP_00860774	short-chain dehydrogenase	261	922632	923417	+	VFG0934	2,3-dihydro-2,3-dihydroxybenzoate dehydrogenase [Escherichia coli CFT073]	248	25.97	181	8.00E-11	57.4
549475284	YP_00860895	glucose-1-phosphate thymidylyltransferase	295	2114959	2115846	-	VFG0964	UDP-glucose pyrophosphorylase [Streptococcus pyogenes SF370]	304	27.04	270	2.00E-14	68.9
549475201	YP_00860886	aminopeptidase	464	2032249	2033643	-	VFG0974	C5a peptidase precursor [Streptococcus pyogenes SF370]	1181	37.19	121	1.00E-11	63.5
549475148	YP_00860881	racemase	234	1976808	1977512	+	VFG1024	unknown [Shigella flexneri (serotype 2a) YSH6000]	229	34.81	135	3.00E-19	80.5
549474952	YP_00860861	tyrosine recombinase XerC	260	1779415	1780197	+	VFG1028	Tn21 integrase IntI1 [Shigella flexneri (serotype 2a) YSH6000]	337	28.2	266	3.00E-22	90.5
549474313	YP_00860797	MFS transporter	403	1145585	1146796	+	VFG1036	tetracycline resistance protein TetA(B) [Shigella flexneri (serotype 2a) YSH6000]	401	25.14	362	1.00E-12	65.5
549474103	YP_00860775	transcriptional regulator	294	933783	934667	+	VFG1038	putative transcriptional regulator TetD [Shigella flexneri (serotype 2a) YSH6000]	138	40.78	103	5.00E-23	90.1
549473926	YP_00860758	iron ABC transporter ATP-binding protein	255	772003	772770	+	VFG1042	ATP-binding protein FecE [Shigella flexneri (serotype 2a) YSH6000]	255	41.67	252	7.00E-62	195
549477195	YP_00861088	iron ABC transporter permease	351	3897098	3898153	-	VFG1043	FecD [Shigella flexneri (serotype 2a) YSH6000]	318	38.67	300	5.00E-58	190
549473927	YP_00860758	ferrichrome ABC transporter substrate-binding protein	342	772967	773995	+	VFG1045	FecB [Shigella flexneri (serotype 2a) YSH6000]	300	25.38	264	1.00E-15	72.8
549476788	YP_00861047	hypothetical protein	229	3480606	3481295	-	VFG1066	unknown [Shigella flexneri (serotype 2a) YSH6000]	163	42.11	114	6.00E-29	105
549476581	YP_00861026	ATP-dependent helicase	562	3286352	3288040	+	VFG1093	helicase, putative [Vibrio cholerae N16961]	940	24.51	506	4.00E-20	90.9
549474086	YP_00860774	transcriptional regulator	241	915051	915776	-	VFG1108	conserved hypothetical protein [Vibrio cholerae N16961]	278	29.59	196	1.00E-17	76.3
549475023	YP_00860868	dihydrodipicolinate synthase	289	1846291	1847160	+	VFG1109	N-acetylneuraminate lyase, putative [Vibrio cholerae N16961]	298	28.93	280	9.00E-29	109
549474611	YP_00860827	C4-dicarboxylate ABC transporter	653	1433974	1435935	+	VFG1110	conserved hypothetical protein [Vibrio cholerae N16961]	427	29.45	163	1.00E-11	63.2
549477877	YP_00861157	C4-dicarboxylate ABC transporter permease	181	4660318	4660863	-	VFG1111	conserved hypothetical protein [Vibrio cholerae N16961]	173	26.9	145	2.00E-12	59.3
549477432	YP_00861112	C4-dicarboxylate ABC transporter	333	4161008	4162009	-	VFG1112	C4-dicarboxylate-binding periplasmic protein [Vibrio cholerae N16961]	321	26.67	315	1.00E-37	136
549474030	YP_00860768	xylose repressor	399	855037	856236	-	VFG1115	ROK family protein [Vibrio cholerae N16961]	287	29.8	151	3.00E-12	63.2
549477292	YP_00861098	N-acetylglucosamine-6-phosphate deacetylase	391	3990388	3991563	+	VFG1116	N-acetylglucosamine-6-phosphate deacetylase [Vibrio cholerae N16961]	378	32.45	379	7.00E-40	144
549473885	YP_00860753	bacitracin ABC transporter ATP-binding protein	307	737668	738591	+	VFG1206	iron(III) ABC transporter, ATP-binding protein [Neisseria meningitidis MC58 (serogroup B)]	352	29.07	227	4.00E-24	97.1
549473906	YP_00860756	hypothetical protein	417	751928	753181	+	VFG1213	two-component sensor PilS [Pseudomonas aeruginosa PAO1]	530	26.7	221	1.00E-16	78.2
549474041	YP_00860769	transcriptional regulator	235	868557	869264	+	VFG1214	two-component response regulator PilR [Pseudomonas aeruginosa PAO1]	445	35.19	108	2.00E-13	65.1
549475094	YP_00860876	chemotaxis protein CheY	119	1918036	1918395	+	VFG1226	twitching motility protein PilH [Pseudomonas aeruginosa PAO1]	121	37.19	121	2.00E-21	81.6
549474695	YP_00860835	methyl-accepting chemotaxis protein	602	1525524	1527332	+	VFG1228	twitching motility protein PilJ [Pseudomonas aeruginosa PAO1]	682	31.82	220	1.00E-26	111
549476154	YP_00860983	amino acid transporter	207	2892069	2892692	+	VFG1235	probable chemotaxis protein [Pseudomonas aeruginosa PAO1]	203	27.01	174	9.00E-11	55.5
549474966	YP_00860862	flagellar basal body rod modification protein FlgD	211	1791955	1792590	+	VFG1237	flagellar basal-body rod modification protein FlgD [Pseudomonas aeruginosa PAO1]	237	40	75	7.00E-15	67.4
549477349	YP_00861104	flagellar hook protein FlgK	532	4049203	4050801	-	VFG1244	flagellar hook-associated protein 1 FlgK [Pseudomonas aeruginosa PAO1]	683	28.42	373	1.00E-25	107
549477348	YP_00861104	flagellar hook-associated protein FlgL	287	4048328	4049191	-	VFG1245	flagellar hook-associated protein type 3 FlgL [Pseudomonas aeruginosa PAO1]	439	29.19	185	3.00E-19	83.6
549476226	YP_00860990	transcriptional regulator	460	2961743	2963125	-	VFG1249	two-component response regulator [Pseudomonas aeruginosa PAO1]	473	47.37	228	5.00E-61	206
549474958	YP_00860862	flagellar hook-basal body protein FliE	102	1784524	1784832	+	VFG1250	flagellar hook-basal body complex protein FliE [Pseudomonas aeruginosa PAO1]	109	46.67	75	8.00E-17	68.6
549474960	YP_00860862	flagellar motor switch protein FliG	338	1786509	1787525	+	VFG1252	flagellar motor switch protein FliG [Pseudomonas aeruginosa PAO1]	338	33.64	330	4.00E-69	219
549474972	YP_00860863	flagellar motor switch protein FliN	408	1795590	1796816	+	VFG1257	flagellar motor switch protein FliN [Pseudomonas aeruginosa PAO1]	157	57.47	87	9.00E-30	110
549474976	YP_00860863	flagellar biosynthesis protein FliQ	89	1798593	1798862	+	VFG1260	flagellar biosynthetic protein FliQ [Pseudomonas aeruginosa PAO1]	89	38.2	89	2.00E-20	77
549474934	YP_00860859	signal recognition particle protein Srp54	449	1762574	1763923	+	VFG1264	flagellar biosynthesis protein FlhF [Pseudomonas aeruginosa PAO1]	429	30.81	198	2.00E-15	74.3
549474981	YP_00860864	cobyrinic acid a,c-diamide synthase	290	1804142	1805014	+	VFG1265	flagellar synthesis regulator FleN [Pseudomonas aeruginosa PAO1]	280	32.57	218	3.00E-35	127
549474189	YP_00860784	multidrug ABC transporter ATP-binding protein	582	1008886	1010634	+	VFG1269	cyclolysin secretion ATP-binding protein [Bordetella pertussis Tohama I]	712	34.29	560	1.00E-104	331
549474245	YP_00860790	glycosyl transferase	470	1073555	1074967	+	VFG1285	intercellular adhesion protein A [Staphylococcus aureus MW2]	412	26.35	463	2.00E-28	112
549477437	YP_00861113	hypothetical protein	319	4165857	4166816	-	VFG1287	intercellular adhesion protein B [Staphylococcus aureus MW2]	290	31.46	213	7.00E-22	90.1
549477606	YP_00861130	capsular polysaccharide biosynthesis protein	246	4372397	4373137	-	VFG1297	truncated capsular polysaccharide synthesis enzyme Cap5A [Staphylococcus aureus MW2]	171	38.65	163	3.00E-33	118
549477605	YP_00861130	tyrosine protein kinase	234	4371703	4372407	-	VFG1298	capsular polysaccharide synthesis enzyme Cap8B [Staphylococcus aureus MW2]	228	42.11	209	8.00E-63	196
549477604	YP_00861130	tyrosine protein phosphatase	254	4370840	4371604	-	VFG1299	capsular polysaccharide synthesis enzyme Cap8C [Staphylococcus aureus MW2]	254	40.55	254	2.00E-65	204
549477603	YP_00861129	polysaccharide biosynthesis protein EpsC	606	4368745	4370565	-	VFG1300	capsular polysaccharide synthesis enzyme Cap8D [Staphylococcus aureus MW2]	607	56.48	602	0	647
549475276	YP_00860894	UDP-N-acetylglucosamine 4,6-dehydratase	329	2107824	2108813	+	VFG1301	capsular polysaccharide synthesis enzyme Cap8E [Staphylococcus aureus MW2]	342	45.19	270	8.00E-73	229
549477287	YP_00861098	acetyltransferase	177	3986174	3986707	-	VFG1306	capsular polysaccharide synthesis enzyme Cap8J [Staphylococcus aureus MW2]	185	38.2	89	2.00E-11	56.2
549477601	YP_00861129	UDP-galactose phosphate transferase	214	4366786	4367430	-	VFG1309	capsular polysaccharide synthesis enzyme Cap8M [Staphylococcus aureus MW2]	185	42.39	184	3.00E-47	154
549477595	YP_00861129	UDP-N-acetyl-D-glucosamine dehydrogenase	441	4358957	4360282	-	VFG1311	capsular polysaccharide synthesis enzyme Cap8O [Staphylococcus aureus MW2]	420	33.65	419	2.00E-76	244
549477406	YP_00861110	peptidase M4	549	4128425	4130074	-	VFG1314	zinc metalloproteinase aureolysin [Staphylococcus aureus MW2]	509	46.27	469	3.00E-123	372
549475785	YP_00860945	ABC transporter substrate-binding protein	380	2567056	2568198	+	VFG1330	laminin-binding surface protein [Streptococcus agalactiae 2603V/R]	306	32.34	167	1.00E-22	93.2
549475285	YP_00860895	acetyltransferase	212	2115888	2116526	-	VFG1336	neuD protein [Streptococcus agalactiae 2603V/R]	209	28.64	206	5.00E-21	85.1
549477804	YP_00861150	hypothetical protein	329	4593679	4594668	-	VFG1341	glycosyl transferase CpsJ(V) [Streptococcus agalactiae 2603V/R]	321	33	203	1.00E-25	102
549478120	YP_00861182	sporulation initiation inhibitor Soj	253	4875044	4875805	-	VFG1349	cpsD protein [Streptococcus agalactiae 2603V/R]	229	29.87	154	4.00E-11	57.8
549476206	YP_00860988	hypothetical protein	300	2945800	2946702	-	VFG1352	capsular polysaccharide biosynthesis protein CpsA [Streptococcus agalactiae 2603V/R]	485	30.96	197	1.00E-14	70.5
549477623	YP_00861131	hypothetical protein	656	4407148	4409118	-	VFG1356	endo-beta-N-acetylglucosaminidase [Streptococcus pneumoniae TIGR4]	658	33.33	210	2.00E-20	92
549474452	YP_00860811	transcriptional regulator	313	1275405	1276346	+	VFG1365	capsular polysaccharide biosynthesis protein Cps4A [Streptococcus pneumoniae TIGR4]	481	29.29	198	1.00E-13	67.8
549477600	YP_00861129	hypothetical protein	375	4365564	4366691	-	VFG1372	capsular polysaccharide biosynthesis protein Cps4H [Streptococcus pneumoniae TIGR4]	372	27.1	310	3.00E-20	87.8
549477687	YP_00861138	UDP-N-acetylglucosamine 2-epimerase	382	4480933	4482081	-	VFG1373	UDP-N-acetylglucosamine-2-epimerase [Streptococcus pneumoniae TIGR4]	365	62.78	360	2.00E-171	484
549475070	YP_00860873	transcriptional regulator	134	1898508	1898912	+	VFG1379	hspR [Mycobacterium tuberculosis H37Rv]	126	35.63	87	7.00E-12	55.8
549473423	YP_00860707	isocitrate lyase	427	304786	306069	+	VFG1381	aceA [Mycobacterium tuberculosis H37Rv]	428	69.52	420	0	609
549476502	YP_00861018	pyrroline-5-carboxylate reductase	281	3209514	3210359	+	VFG1384	proC [Mycobacterium tuberculosis H37Rv]	295	34.83	290	7.00E-33	120
549474243	YP_00860790	hypothetical protein	557	1070836	1072509	+	VFG1386	phoP [Mycobacterium tuberculosis H37Rv]	247	30.51	118	2.00E-13	67
549476199	YP_00860987	transcriptional regulator	227	2940787	2941470	-	VFG1389	hypothetical protein Rv0903c [Mycobacterium tuberculosis H37Rv]	236	42.08	202	4.00E-46	153
549477762	YP_00861146	chemotaxis protein CheY	225	4552604	4553281	-	VFG1390	hypothetical protein Rv0981 [Mycobacterium tuberculosis H37Rv]	230	36	225	3.00E-38	132
549474346	YP_00860800	RNA polymerase sigma factor SigY	187	1181542	1182105	+	VFG1392	sigE [Mycobacterium tuberculosis H37Rv]	257	32.39	142	3.00E-18	76.6
549476122	YP_00860979	anthranilate phosphoribosyltransferase	341	2854690	2855715	-	VFG1398	trpD [Mycobacterium tuberculosis H37Rv]	370	33.82	346	3.00E-49	168
549475071	YP_00860873	glutamine synthetase	444	1898947	1900281	+	VFG1399	glnA1 [Mycobacterium tuberculosis H37Rv]	478	38.59	482	3.00E-102	313
549477835	YP_00861153	alkyl hydroperoxide reductase subunit C	187	4627675	4628238	+	VFG1404	ahpC [Mycobacterium tuberculosis H37Rv]	195	33.9	177	2.00E-29	107
549476650	YP_00861033	RNA polymerase sigma factor RpoD	374	3344869	3345993	-	VFG1405	sigA [Mycobacterium tuberculosis H37Rv]	528	63.04	303	2.00E-130	385
549475613	YP_00860928	quinone oxidoreductase	325	2425350	2426327	+	VFG1408	mas [Mycobacterium tuberculosis H37Rv]	2111	28.49	337	4.00E-29	116
549476829	YP_00861051	isopropylmalate isomerase	192	3530007	3530585	-	VFG1411	leuD [Mycobacterium tuberculosis H37Rv]	198	43.46	191	2.00E-39	133
549475217	YP_00860888	hypothetical protein	320	2051002	2051964	+	VFG1412	sigH [Mycobacterium tuberculosis H37Rv]	216	27.37	190	2.00E-13	64.7
549474871	YP_00860853	RNA polymerase sigma 70	270	1692566	1693378	+	VFG1413	sigF [Mycobacterium tuberculosis H37Rv]	261	32.14	224	2.00E-27	105
549476363	YP_00861004	aspartate decarboxylase	127	3091616	3091999	-	VFG1416	panD [Mycobacterium tuberculosis H37Rv]	139	51.22	123	7.00E-38	125
549476364	YP_00861004	pantoate--beta-alanine ligase	282	3092021	3092869	-	VFG1417	panC [Mycobacterium tuberculosis H37Rv]	309	46.46	198	1.00E-50	169
549476452	YP_00861013	superoxide dismutase	296	3172335	3173225	-	VFG1421	sodA [Mycobacterium tuberculosis H37Rv]	207	43.37	196	3.00E-55	177
549473094	YP_00860751	DNA methyltransferase	416	715117	716367	-	VFG1454	putative DNA methylase [Escherichia coli 536]	310	32.24	183	3.00E-22	92.8

549475090	YP_00860875	multidrug ABC transporter ATP-binding protein	587	1913002	1914765	+	VFG1557	HlyB protein [Escherichia coli 536]	707	30.96	575	8.00E-83	273
549477117	YP_00861081	oxidoreductase	197	3826211	3826804	-	VFG1561	hypothetical protein [Escherichia coli 536]	256	25	172	1.00E-12	61.2
549475918	YP_00860959	transcriptional regulator	230	2677518	2678210	-	VFG1563	hypothetical protein [Escherichia coli 536]	239	33.19	226	5.00E-45	150
549474263	YP_00860792	gluconate transporter	447	1099173	1100516	+	VFG1583	hypothetical protein [Escherichia coli 536]	445	36.01	436	4.00E-82	260
549474759	YP_00860842	LysR family transcriptional regulator	294	1586402	1587286	+	VFG1584	hypothetical protein [Escherichia coli 536]	314	32	125	2.00E-12	63.2
549476052	YP_00860972	quaternary ammonium transporter	123	2788755	2789126	-	VFG1586	hypothetical protein [Escherichia coli 536]	109	32.35	102	3.00E-20	78.2
549473359	YP_00860700	multidrug transporter	104	230009	230323	+	VFG1587	hypothetical protein [Escherichia coli 536]	133	32	100	4.00E-11	53.1
549475780	YP_00860945	alcohol dehydrogenase	398	2561854	2563050	-	VFG1593	hypothetical protein [Escherichia coli 536]	384	30.95	391	4.00E-53	181
549474735	YP_00860839	aminotransferase A	384	1567800	1568954	-	VFG1604	hypothetical protein [Escherichia coli 536]	392	22.13	366	3.00E-17	79
549474713	YP_00860837	MarR family transcriptional regulator	146	1546582	1547022	+	VFG1649	SfaX protein [Escherichia coli 536]	166	26.09	115	7.00E-12	56.6
549475763	YP_00860943	multidrug ABC transporter ATP-binding protein	585	2548183	2549940	-	VFG1653	ABC transport protein [Escherichia coli 536]	1219	32.87	499	2.00E-57	206
549475872	YP_00860954	4-hydroxytetrahydrobiopterin dehydratase	392	2636398	2637576	-	VFG1661	hypothetical protein [Escherichia coli 536]	399	27.23	415	2.00E-36	135
549476739	YP_00861042	aspartyl-tRNA synthase	591	3434002	3435777	-	VFG1668	putative lysil-tRNA synthetase LysU [Escherichia coli 536]	498	24.49	294	3.00E-24	103
549474789	YP_00860845	arginine decarboxylase	489	1616306	1617775	-	VFG1670	putative cadaverin decarboxylase [Escherichia coli 536]	708	25.45	554	6.00E-30	120
549477993	YP_00861169	MarR family transcriptional regulator	141	4746599	4747024	-	VFG1688	putative MarR-family transcriptional regulator [Escherichia coli 536]	174	29.69	128	3.00E-12	57.4
549475917	YP_00860959	hypothetical protein	745	2675303	2677540	-	VFG1701	Hypothetical protein [Escherichia coli CFT073]	490	24.71	255	4.00E-17	81.6
549475619	YP_00860929	transcriptional regulator	234	2434313	2435017	-	VFG1702	Putative response regulator [Escherichia coli CFT073]	240	38.6	228	2.00E-48	159
549473500	YP_00860714	hypothetical protein	510	372030	373562	+	VFG1746	putative two-component response regulator [Yersinia pestis CO92]	210	29.34	167	2.00E-14	69.3
549475324	YP_00860899	long-chain fatty acid--CoA ligase	519	2160406	2161965	+	VFG1817	mbtA [Mycobacterium tuberculosis H37Rv]	565	26.05	522	7.00E-39	145
549474490	YP_00860814	LuxR family transcriptional regulator	210	1315062	1315694	+	VFG1824	hypothetical protein Rv3133c [Mycobacterium tuberculosis H37Rv]	217	31.55	206	2.00E-34	121
549475675	YP_00860934	sensor histidine kinase	378	2481818	2482954	-	VFG1825	hypothetical protein Rv3132c [Mycobacterium tuberculosis H37Rv]	578	31.96	194	5.00E-18	82
549476744	YP_00861043	GTP pyrophosphokinase	731	3440163	3442358	-	VFG1826	relA [Mycobacterium tuberculosis H37Rv]	790	43.19	720	0	646
549473611	YP_00860725	peptide ABC transporter permease	497	472085	473578	-	VFG1857	IraAB [Legionella pneumophila Philadelphia 1]	502	23.48	511	2.00E-33	129
549476627	YP_00861031	superoxide dismutase	202	3324045	3324653	-	VFG1867	superoxide dismutase [Legionella pneumophila Philadelphia 1]	196	49.49	196	1.00E-66	203
549476591	YP_00861027	competence protein ComG	343	3291718	3292749	-	VFG1881	(type IV) pilus assembly protein PilC [Legionella pneumophila Philadelphia 1]	406	23.3	352	1.00E-14	71.2
549476795	YP_00861048	prepilin peptidase	247	3486219	3486962	-	VFG1882	type 4 (IV) prepilin-like protein leader peptide processing enzyme PilD [Legionella pneumophila Philadelphia 1]	289	32.62	279	3.00E-37	132
549477345	YP_00861104	carbon storage regulator	77	4047032	4047265	-	VFG1886	carbon storage regulator RsmA [Legionella pneumophila Philadelphia 1]	78	53.7	54	6.00E-16	64.7
549475134	YP_00860880	histidine kinase	376	1961896	1963026	+	VFG1888	sensory box histidine kinase/response regulator [Legionella pneumophila Philadelphia 1]	910	25.45	220	1.00E-18	84
549477713	YP_00861141	chemotaxis protein CheY	124	4506282	4506656	-	VFG1889	response regulator GacA [Legionella pneumophila Philadelphia 1]	225	39.32	117	3.00E-18	74.7
549474971	YP_00860863	flagellar motor switch protein FliM	332	1794602	1795600	+	VFG1893	flagellar motor switch protein [Campylobacter jejuni NCTC 11168]	359	37.8	328	1.00E-69	221
549474924	YP_00860858	cell division protein FtsY	329	1748440	1749429	+	VFG1895	flagellar biosynthesis protein [Campylobacter jejuni NCTC 11168]	484	26.24	202	1.00E-14	70.9
549474978	YP_00860864	flagellar biosynthesis protein FlhB	360	1799646	1800728	+	VFG1900	flagellar biosynthetic protein [Campylobacter jejuni NCTC 11168]	362	43.14	350	1.00E-92	282
549473781	YP_00860743	flagellar motor protein MotA	264	628518	629312	+	VFG1902	putative flagellar motor proton channel [Campylobacter jejuni NCTC 11168]	258	34.92	252	3.00E-43	147
549474975	YP_00860863	flagellar biosynthesis protein FliP	221	1797915	1798580	+	VFG1915	flagellar biosynthesis protein [Campylobacter jejuni NCTC 11168]	244	55.92	211	6.00E-84	250
549474977	YP_00860863	flagellar biosynthesis protein FliR	260	1798867	1799649	+	VFG1918	flagellar biosynthetic protein [Campylobacter jejuni NCTC 11168]	255	30.17	242	3.00E-34	123
549474185	YP_00860784	enoyl-ACP reductase	261	1006052	1006837	+	VFG1922	putative oxidoreductase (flagellin modification) [Campylobacter jejuni NCTC 11168]	256	28.57	266	7.00E-22	89
549475315	YP_00860898	hypothetical protein	363	2151081	2152172	+	VFG1965	putative aminotransferase [Campylobacter jejuni NCTC 11168]	367	27.17	368	4.00E-30	116
549476521	YP_00861020	ABC transporter substrate-binding protein	267	3227339	3228142	-	VFG1983	probable ABC-type amino-acid transporter periplasmic solute-binding protein [Campylobacter jejuni NCTC 11168]	259	26.49	268	3.00E-16	72.4
549475273	YP_00860894	adenylylsulfate kinase	204	2104104	2104718	-	VFG1988	possible adenylylsulfate kinase [Campylobacter jejuni NCTC 11168]	170	35.15	165	1.00E-27	101
549476956	YP_00861064	flagellar motor protein MotS	246	3669593	3670333	-	VFG2008	flagellar motor protein MotB [Legionella pneumophila Philadelphia 1]	273	32.79	244	1.00E-36	129
549476957	YP_00861064	flagellar motor protein MotP	272	3670323	3671141	-	VFG2009	flagellar motor protein MotA [Legionella pneumophila Philadelphia 1]	257	33.46	260	6.00E-46	154
549474980	YP_00860864	flagellar biosynthesis regulator FlhF	381	1803004	1804149	+	VFG2011	flagellar GTP-binding protein FlhF [Legionella pneumophila Philadelphia 1]	386	31.06	367	1.00E-36	135
549475357	YP_00860902	hypothetical protein	378	2190278	2191414	-	VFG2045	virulence sensor protein [Bordetella pertussis Tohama I]	1238	27.13	258	3.00E-19	86.3
549476592	YP_00861027	competence protein ComG	355	3292736	3293803	-	VFG2048	putative type II secretion protein [Shigella dysenteriae Sd197]	497	42.51	287	2.00E-66	217
549474907	YP_00860856	serine/threonine protein kinase	658	1730662	1732638	+	VFG2060	serine/threonine protein kinase PpkA [Pseudomonas aeruginosa PAO1]	1032	40.08	242	7.00E-52	190
549474906	YP_00860856	protein phosphatase	253	1729907	1730668	+	VFG2061	probable phosphoprotein phosphatase [Pseudomonas aeruginosa PAO1]	242	33.33	243	2.00E-28	107
549476162	YP_00860983	hypothetical protein	274	2903065	2903889	+	VFG2112	VipF, N-terminal acetyltransferase, GNAT family [Legionella pneumophila Philadelphia 1]	286	27.82	284	1.00E-25	100
549474962	YP_00860862	ATP synthase	438	1788276	1789592	+	VFG2144	type III secretion system ATPase [Chlamydia trachomatis D/UW-3/CX]	442	51.52	429	2.00E-147	428
549476296	YP_00860997	hypothetical protein	307	3031249	3032172	-	VFG2157	lipoprotein [Listeria monocytogenes (serovar 1/2a) EGD-e]	310	31.63	313	3.00E-53	176
549476575	YP_00861026	octanoyltransferase	278	3280210	3281046	-	VFG2158	lipoate protein ligase [Listeria monocytogenes (serovar 1/2a) EGD-e]	331	28.16	277	6.00E-21	87
549474893	YP_00860855	hypothetical protein	570	1715076	1716788	-	VFG2160	fibronectin-binding protein [Listeria monocytogenes (serovar 1/2a) EGD-e]	570	54.29	571	0	630
549474881	YP_00860854	peptidase A8	162	1701736	1702224	+	VFG2161	signal peptidase II [Listeria monocytogenes (serovar 1/2a) EGD-e]	154	60.93	151	3.00E-57	176
549475140	YP_00860880	hypothetical protein	329	1967137	1968126	-	VFG2162	bile salt hydrolase [Listeria monocytogenes (serovar 1/2a) EGD-e]	325	27.16	324	7.00E-24	96.3
549475221	YP_00860888	DNA-binding protein	71	2054925	2055140	+	VFG2168	transcriptional regulator, Cro/CI family [Enterococcus faecalis V583]	66	53.97	63	6.00E-16	64.3
549475416	YP_00860908	serine protease	320	2256353	2257315	-	VFG2177	cytolysin activator [Enterococcus faecalis MMH594]	412	30.71	267	3.00E-18	81.3
549475168	YP_00860883	LacI family transcriptional regulator	331	2000328	2001323	+	VFG2197	sugar-binding transcriptional regulator, LacI family [Enterococcus faecalis V583]	336	26.1	341	3.00E-31	118
549477463	YP_00861115	spore coat protein	410	4201055	4202287	-	VFG2226	perosamine synthetase [Brucella melitensis 16M]	380	33.78	296	3.00E-42	151
549474903	YP_00860856	methionyl-tRNA formyltransferase	318	1726522	1727478	+	VFG2230	GDP-mannose 4,6-dehydratase / GDP-4-amino-4,6-dideoxy-D-mannose formyltransferase [Brucella melitensis 16M]	259	32.94	170	3.00E-19	82.4
549474988	YP_00860865	RNA polymerase sigma factor SigD	253	1810063	1810824	+	VFG2319	RNA polymerase sigma factor for flagellar operon [Yersinia enterocolitica 8081]	240	35.68	227	7.00E-46	153
549477341	YP_00861103	flagellar hook protein	698	4039755	4041851	-	VFG2324	flagellar hook-associated protein 2 [Yersinia enterocolitica 8081]	466	24.36	312	8.00E-13	67.4
549477340	YP_00861103	flagellar biosynthesis protein FliS	133	4039311	4039712	-	VFG2325	flagellar protein FliS [Yersinia enterocolitica 8081]	132	36.36	110	7.00E-18	72.8
549474957	YP_00860861	flagellar basal body rod protein FlgC	149	1784061	1784510	+	VFG2350	flagellar basal-body rod protein FlgC [Yersinia enterocolitica 8081]	134	44.3	149	4.00E-36	121
549477581	YP_00861127	UDP-glucose 4-epimerase	295	4344089	4344976	-	VFG2361	UDP-glucose 4-epimerase [Yersinia enterocolitica 8081]	336	53.56	295	1.00E-110	324
549473297	YP_00860693	phosphoglucosamine mutase	449	177927	179276	+	VFG2362	phosphomannomutase [Yersinia enterocolitica 8081]	456	24.34	452	1.00E-31	122
549475279	YP_00860894	hypothetical protein	234	2110569	2111273	-	VFG2368	putative glycosyltransferase [Yersinia enterocolitica 8081]	288	30.05	203	7.00E-23	91.7
549475281	YP_00860894	spore coat protein	152	2112705	2113163	-	VFG2375	putative epimerase [Yersinia enterocolitica 8081]	174	27.59	145	3.00E-12	58.2
549475066	YP_00860873	stage V sporulation protein K	324	1893626	1894600	+	VFG2380	hypothetical protein [Mycobacterium tuberculosis H37Rv]	573	36.69	278	1.00E-50	175
549477373	YP_00861106	alkaline serine protease	1170	4072858	4076370	-	VFG2391	MEMBRANE-ANCHORED MYCOSIN MYCP1 (SERINE PROTEASE) (SUBTILISIN-LIKE PROTEASE) (SUBTILASE-LIKE) (MYCOSIN-1) [Mycobacterium tuberculosis H37R	446	29.11	371	1.00E-16	80.5
549475028	YP_00860869	cell division protein FtsK	782	1850742	1853090	+	VFG2409	hypothetical protein [Staphylococcus aureus MW2]	1479	33.19	232	7.00E-22	98.2
549475523	YP_00860919	hypothetical protein	371	2348955	2350070	+	VFG2420	Adenosine synthase A [Staphylococcus aureus MW2]	772	23.51	285	2.00E-13	68.2
549474968	YP_00860863	flagellar basal body rod protein FlgG	270	1793057	1793869	+	VFG2513	flagellar basal body rod protein FlgG [Burkholderia pseudomallei K96243]	262	40.22	271	5.00E-49	162
549474979	YP_00860864	flagellar biosynthesis protein FlhA	678	1800971	1803007	+	VFG2522	flagellar biosynthesis protein FlhA [Burkholderia pseudomallei K96243]	700	42.3	688	2.00E-180	531
549474946	YP_00860860	hypothetical protein	99	1771514	1771813	+	VFG2523	flagellar biosynthesis protein FlhB [Burkholderia pseudomallei K96243]	405	46.84	79	9.00E-14	63.2
549474973	YP_00860863	chemotaxis protein CheY	120	1796838	1797200	+	VFG2525	chemotaxis protein CheY [Burkholderia pseudomallei K96243]	142	36.67	120	1.00E-19	77.4
549474982	YP_00860864	chemotaxis protein CheY	363	1805015	1806106	+	VFG2526	chemotaxis-specific methylesterase [Burkholderia pseudomallei K96243]	367	43.02	358	6.00E-90	275
549474986	YP_00860864	chemotaxis protein CheD	165	1809301	1809798	+	VFG2527	chemoreceptor glutamine deamidase CheD [Burkholderia pseudomallei K96243]	234	31.16	138	1.00E-16	71.2
549476150	YP_00860982	chemotaxis protein R	278	2886529	2887365	-	VFG2528	chemotaxis protein methyltransferase [Burkholderia pseudomallei K96243]	315	30.16	189	1.00E-17	77.8
549476048	YP_00860972	hypothetical protein	565	2782243	2783940	-	VFG2529	methyl-accepting chemotaxis protein I [Burkholderia pseudomallei K96243]	673	26.67	345	2.00E-20	91.7
549474984	YP_00860864	chemotaxis protein CheW	154	1808195	1808659	+	VFG2530	chemotaxis protein CheW [Burkholderia pseudomallei K96243]	175	39.58	144	1.00E-32	113
549474983	YP_00860864	chemotaxis protein CheA	674	1806127	1808151	+	VFG2531	chemotaxis two-component sensor kinase CheA [Burkholderia pseudomallei K96243]	747	42.71	398	2.00E-98	319
549475334	YP_00860900	2-amino-3-ketobutyrate CoA ligase	411	2172390	2173625	+	VFG2546	acyl-CoA transferase [Burkholderia pseudomallei K96243]	439	33.24	361	2.00E-58	196
549474542	YP_00860820	3-oxoacyl-ACP synthase	413	1369001	1370242	+	VFG2548	capsular polysaccharide biosynthesis fatty acid synthase [Burkholderia pseudomallei K96243]	2546	28.05	385	2.00E-31	125
549476500	YP_00861018	oxidoreductase	265	3207160	3207957	-	VFG2550	capsular polysaccharide biosynthesis dehydrogenase/reductase [Burkholderia pseudomallei K96243]	262	28.79	257	2.00E-17	76.3
549475278	YP_00860894	hypothetical protein	312	2109634	2110572	+	VFG2556	GDP sugar epimerase/dehydratase [Burkholderia pseudomallei K96243]	337	29.02	348	4.00E-33	123

Table S5: Comparative analysis of the presence or absence of common methyltransferases between *Bacillus* sp. NRRL B-14911 and other *Bacillus* species.

Methyltransferases	*Bacillus* sp. NRRL B-14911	*B. subtilis* Strain 168	*B. megaterium* DSM 319	*B. thuringiensis* serovar kurstaki strain HD73	*B. cereus* ATCC 14579
16S rRNA methyltransferase	+	+	+	+	+
23S rRNA (uracil-5-)-methyltransferase RumA	-	-	+	+	-
23S rRNA methyltransferase	+	-	+	+	+
3-demethylubiquinone-9 3-methyltransferase	+	-	+	-	-
3-methyl-2-oxobutanoate hydroxymethyltransferase	+	+	+	+	+
50S ribosomal protein L11 methyltransferase	-	+	+	-	-
**50S rRNA methyltransferase**	**+**	**-**	**-**	**-**	**-**
5-methyltetrahydrofolate--homocysteine methyltransferase	+	-	-	-	+
5-methyltetrahydropteroyltriglutamate/homocysteine S-methyltransferase	+	+	+	+	+
acyl-CoA O-methyltransferase	-	+	-	-	-
adenine-specific methyltransferase	-	-	-	-	+
AdoMet-dependent methyltransferase	-	+	-	-	-
bifunctional homocysteine S-methyltransferase/5,10-methylenetetrahydrofolate	-	+	-	-	+
caffeoyl-CoA O-methyltransferase	-	-	-	-	+
chemotaxis protein methyltransferase	-	-	+	+	+
Corrin/porphyrin methyltransferase	-	-	-	-	+
DNA (cytosine-5-)-methyltransferase	-	+	-	-	-
DNA methyltransferase	+	+	+	-	-
glycine cleavage system aminomethyltransferase T	-	+	-	+	+
Glycosyltransferase / Glycosyltransferase / O-methyltransferase	-	-	-	+	-
homocysteine methyltransferase	-	+	+	-	-
homocysteine S-methyltransferase/5,10-methylenetetrahydrofolate reductase	-	-	+	-	-
isoprenylcysteine carboxyl methyltransferase	+	-	+	-	-
**lysine methyltransferase**	**+**	**-**	**-**	**-**	**-**
macrocin O-methyltransferase	-	-	-	+	+
menaquinone methyltransferase	-	+	-	-	-
methylated-DNA-protein-cysteine S-methyltransferase	+	-	-	+	-
methyltetrahydrofolate methyltransferase	-	+	-	-	-
methyltransferase	+	+	+	+	+
methyltransferase domain-containing protein	-	-	+	-	-
methyltransferase type 11	+	-	-	+	-
methyltransferase type 12	+	-	-	+	-
methyltransferase, CheR	-	-	+	+	-
methyltransferase, UbiE/COQ5	-	-	-	+	-
**N5-glutamine S-adenosyl-L-methionine-dependent methyltransferase**	**+**	**-**	**-**	**-**	**-**
nucleic acid methyltransferase	-	+	-	-	-
O6-methylguanine-DNA methyltransferase	-	+	-	+	+
O-methyltransferase	+	-	+	+	-
phosphatidylethanolamine N-methyltransferase	-	-	+	-	-

Table S5 continued

phospholipid N-methyltransferase	-	-	+	-	-
precorrin-2 C(20)-methyltransferase	-	-	+	-	-
precorrin-4 C(11)-methyltransferase	-	-	+	-	-
protein-(glutamine-N5) methyltransferase	-	-	+	-	-
**protein-L-isoaspartate O-methyltransferase**	**+**	**-**	**-**	**-**	**-**
protein-L-isoD(D-D) O-methyltransferase	-	-	-	+	+
putative RNA methyltransferase	-	-	-	+	-
Ribosomal protein L11 methyltransferase	+	-	-	+	+
RNA methyltransferase	+	+	-	-	-
RNA methyltransferase, RsmE family	-	-	-	+	-
RNA methyltransferase, TrmH family	-	-	-	+	-
RNA methyltransferase, TrmH family, group 2	-	-	-	+	+
rRNA large subunit methyltransferase	-	+	-	-	+
rRNA large subunit methyltransferase N	-	+	-	+	+
rRNA methyltransferase	+	+	-	-	-
rRNA small subunit methyltransferase B	-	-	+	+	-
rRNA small subunit methyltransferase E	-	-	+	-	-
**S-adenosyl-L-methionine-dependent methyltransferase**	**+**	**-**	**-**	**-**	**-**
S-adenosylmethionine:2-demethylmenaquinone methyltransferase	-	-	+	+	-
S-adenosylmethionine-dependent methyltransferase	-	+	+	-	-
S-adenosyl-methyltransferase	-	+	-	-	-
S-adenosyl-methyltransferase MraW	-	-	+	+	+
SAM-dependent methyltransferase	+	-	-	+	+
serine hydroxymethyltransferase	+	+	+	+	+
TrmH family RNA methyltransferase	-	-	+	-	-
tRNA (5-methyl aminomethyl-2-thiouridylate)-methyltransferase	-	-	-	+	-
tRNA (guanine-N(1)-)-methyltransferase	+	+	+	+	+
tRNA (guanine-N(7)-)-methyltransferase	+	+	+	+	+
tRNA (Uracil-5-) -methyltransferase	+	+	-	-	+
tRNA/rRNA methyltransferase TrmH	-	+	-	-	-
tRNA: m1A22 methyltransferase	-	+	+	-	-
tRNA:m(5)U-54 methyltransferase	-	-	-	-	-
UbiE/COQ5 family methyltransferase	+	-	-	+	-
ubiquinone/menaquinone biosynthesis methyltransferase	+	+	+	+	+
uroporphyrin-III C-methyltransferase	+	+	-	+	+
uroporphyrinogen III C-methyltransferase	-	+	+	+	-

Bold, unique to *Bacillus* sp. NRRL B-14911; +, present; -, absent.

Table S6: Comparative analysis of the presence or absence of transporters between *Bacillus* sp. NRRL B-14911 and other *Bacillus* species.

Transporters	*Bacillus* sp. NRRL B-14911	*B. subtilis* Strain 168	*B. megaterium* DSM 319	*B. thuringiensis* serovar kurstaki strain HD73	*B. cereus* ATCC 14579
2-aminoethylphosphonate ABC transporter ATP-binding protein	-	-	-	+	-
2-oxoglutarate/malate transporter	-	-	+	-	-
ABC membrane transporter ATP-binding protein	-	-	+	-	-
ABC transporter	+	-	+	-	+
ABC transporter atpase	-	-	+	-	-
ABC transporter ATP-binding protein	+	+	+	+	+
ABC transporter ATP-binding protein uup	-	-	-	+	+
ABC transporter ATP-binding protein yxdl	-	-	-	-	+
ABC transporter ATP-binding/permease	-	+	-	-	-
ABC transporter binding lipoprotein	-	-	+	-	-
ABC transporter ecsb	-	-	-	-	+
ABC transporter family protein, partial	-	+	-	-	-
ABC transporter membrane protein	-	-	+	-	-
ABC transporter permease	+	+	+	+	+
ABC transporter permease ecsb	-	+	-	-	-
ABC transporter permease, bceb-like protein	-	+	-	-	-
ABC transporter protein	-	-	-	-	+
ABC transporter substrate-binding protein	+	+	-	+	-
ABC transporter, ATP-binding protein	-	-	-	-	+
ABC transporter-associated protein	-	-	-	+	+
ABC transporter-related protein	-	-	-	-	+
ABC-type transporter ATP-binding protein ecsa	-	-	-	+	+
Acetamide transporter	-	-	-	+	+
Acetoin ABC transporter ATP-binding protein	-	-	-	+	-
Acetoin ABC transporter permease	-	+	-	-	-
Acrb/acrd/acrf family transporter	-	-	+	-	-
Aliphatic sulfonate ABC transporter ATP-binding protein	-	-	+	-	-
Aliphatic sulfonate ABC transporter permease	-	-	+	-	-
Aliphatic sulfonates ABC transporter aliphatic sulfonates-binding protein ssua	-	+	-	-	-
Aliphatic sulfonates ABC transporter ATP-binding protein ssub	-	+	-	-	-
Aliphatic sulfonates ABC transporter permease ssuc	-	+	-	-	-
Alkanesulfonate ABC transporter permease	-	-	-	+	-
Alkanesulfonates transporter ATP-binding protein	-	-	-	+	-
Alpha-ketoglutarate transporter, MFS super	-	-	-	-	+
Amino acid ABC transporter (Substrate binding protein)	-	-	-	-	+
Amino acid ABC transporter amino acid-binding protein	-	+	-	-	-
**Amino acid ABC transporter ATP-binding protein**	**+**	**-**	**-**	-	-
Amino acid ABC transporter permease	+	+	-	-	-
**Amino acid ABC transporter, permease protein**	**+**	**-**	**-**	-	-
Amino acid ABC transporter, solute binding protein	-	-	-	-	+

Table S6 continued

Amino acid related metabolite efflux transporter	-	-	+	-	-
Amino acid transporter	+	+	+	-	+
Amino acid transporter lyse	-	-	-	+	-
Amino acid transporter, AAT family	-	-	-	-	+
Amino acid/peptide transporter	-	-	-	-	+
Amino acid/peptide transporter (Peptide:H+ symporter)	-	+	-	-	-
Ammonium transporter	+	+	+	+	+
Anaerobic C4-dicarboxylate membrane transporter	-	-	-	+	+
Anion ABC transporter permease	-	-	+	-	-
Anion transporter	+	-	+	-	-
Anion transporter and exported enzyme	-	-	+	-	-
**Antibiotic ABC transporter ATP-binding protein**	**+**	**-**	**-**	-	-
Arabinogalactan ABC transporter permease	-	-	-	-	+
Arginine ABC transporter arginine-binding protein artp	-	-	-	-	+
Arginine ABC transporter ATP-binding protein	-	+	-	+	-
Arginine ABC transporter permease	+	-	-	+	+
**Arsenic transporter atpase**	**+**	**-**	**-**	-	-
**Arsenite efflux transporter**	**+**	**-**	**-**	-	-
ATP-binding cassette efflux transporter	-	-	+	-	-
ATP-binding teichoic acid transporter component	-	-	+	-	-
Auxin efflux carrier (AEC) family transporter	-	+	-	-	-
Bacitracin ABC efflux transporter ATP-binding protein	-	-	+	-	-
**Bacitracin ABC transporter ATP-binding protein**	**+**	**-**	**-**	-	-
Bacitracin ABC transporter ATP-binding protein bcea	-	+	-	-	-
**Bacitracin ABC transporter permease**	**+**	**-**	**-**	-	-
Bacitracin ABC transporter permease bceb	-	+	-	-	-
Bacitracin ABC transporter, ATP-binding protein	-	-	-	-	+
Bcr/cfla subfamily drug resistance transporter	-	+	-	-	-
Betaine/carnitine/choline transporter (BCCT) family transporter	-	-	-	-	+
**Beta-methylgalactoside transporter**	**+**	**-**	**-**	-	-
Biotin transporter	-	-	+	-	-
Branched chain amino acid ABC transporter	-	-	-	+	-
Branched chain amino acid ABC transporter ATP-binding protein	-	-	-	+	-
Branched chain amino acid ABC transporter carrier protein	-	-	-	+	-
Branched chain amino acid ABC transporter permease	-	-	-	+	+
Branched chain amino acids transporter	-	-	+	-	-
Branched-chain amino acid ABC transporter ATP-binding protein	+	+	-	-	-
Branched-chain amino acid ABC transporter branched-chain amino acid-binding protein	-	+	-	-	-
Branched-chain amino acid ABC transporter permease	+	+	-	-	-
Branched-chain amino acid ABC transporter substrate-binding protein	-	-	-	-	+
Branched-chain amino acid ABC transporter, ATP-binding protein	-	-	-	-	+
Branched-chain amino acid transporter	+	-	+	-	-

Table S6 continued

**C4-dicarboxylate ABC transporter**	+	-	-	-	-
**C4-dicarboxylate ABC transporter permease**	+	-	-	-	-
**C4-dicarboxylate ABC transporter substrate-binding protein**	+	-	-	-	-
C4-dicarboxylate transporter dcta	-	-	+	-	-
Ca2+/citrate complex secondary transporter	-	-	-	+	+
**Cadmium transporter**	+	-	-	-	-
Carbohydrate ABC transporter ATP-binding protein	-	-	+	-	-
Carbohydrate ABC transporter permease	-	+	-	-	-
Carbohydrate ABC transporter permease amyd	-	-	+	-	-
Cation ABC transporter ATP-binding protein	-	+	-	-	-
Cation diffusion facilitator family transporter	-	+	-	-	-
Cation transporter	+	+	-	-	-
Cationic peptides ABC transporter ATP-binding protein	-		+	-	-
Cationic peptides ABC transporter permease	-		+	-	-
Cell division ABC transporter ATP-binding protein ftse	-	+	-	-	-
Cell division ABC transporter permease ftsx	-	+	-	-	-
Cell division ABC transporter, permease protein ftsx	-	-	-	-	+
Cell wall oligopeptide ABC transporter ATP binding protein	-	-	+	-	-
Cell-division ABC transporter	-	-	+	-	-
Cell-division ABC transporter ATP-binding protein	-	-	+	-	-
Choline ABC transporter ATP-binding protein	-	-	+	-	-
Choline ABC transporter choline-binding lipoprotein	-	-	+	-	-
Choline ABC transporter permease	-	-	+	-	-
**Choline transporter**	+	-	-	-	-
Citrate transporter family protein	+	+	+	-	+
**Cobalt ABC transporter ATP-binding protein**	+	-	-	-	-
**Cobalt ABC transporter permease**	+	-	-	-	-
Cobalt ABC transporter permease cbiq	-	+	-	-	-
Cobalt import ABC transporter ATP-binding protein cbio 1	-	+	-	-	-
**Cobalt transporter**	+	-	-	-	-
Cobalt transporter ATP-binding subunit	-	-	+	+	+
Copper insertion chaperone and transporter	-	-	+	-	-
Copper transporter atpase	-	-	+	-	-
Cora-like Mg2+ transporter protein,family protein	-	+	-	-	-
**Corrinoid ABC transporter permease**	+	-	-	-	-
**Cyclodextrin transporter permease**	+	-	-	-	-
**Cysteine ABC transporter permease**	+	-	-	-	-
Cysteine ABC transporter permease/ATP-binding protein cydc	-	+	-	-	-
Cystine ABC transporter ATP-binding protein	-	-	+	-	-
Cystine ABC transporter substrate-binding lipoprotein	-	-	+	-	-
**Daunorubicin ABC transporter ATP-binding protein**	+	-	-	-	-
**Daunorubicin ABC transporter permease**	+	-	-	-	-

Table S6 continued

Dctp family TRAP transporter solute receptor	-	+	-	-	-
Di-/tripeptide transporter	-	-	-	+	+
Dipeptide ABC transporter ATP-binding protein	-	-	+	-	-
Dipeptide ABC transporter dipeptide-binding protein dppe	-	+	-	-	-
Dipeptide ABC transporter permease	-	-	+	-	-
Dipeptide ABC transporter permease dppb	-	+	-	-	-
Dipeptide ABC transporter permease dppc	-	+	-	-	-
Dipeptide ABC transporter substrate-binding protein	-	-	+	-	-
Divalent anion:Na+ symporter (DASS) family transporter	-	+	-	-	-
Divalent cation efflux transporter	-	-	+	-	-
**D-ribose transporter ATP binding protein**	**+**	**-**	**-**	-	-
Drrb family ABC transporter efflux protein	-	-	-	-	+
Drug resistance ABC transporter ATP-binding protein drra	-	+	-	-	-
Drug resistance MFS transporter	-	+	-	-	-
Drug resistance transporter, Bcr/cfla	-	-	-	-	+
Drug resistance transporter, emrb/qaca	-	-	-	-	+
Drug resistance transporter, emrb/qaca family	-	-	-	-	+
Drug/metabolite exporter family transporter	-	+	-	-	-
Drug:H+ antiporter-2 (14 Spanner) (DHA2) family drug resistance MFS transporter	-	-	-	-	+
D-serine/D-alanine/glycine transporter	-	+	-	-	-
Eama family transporter	-	+	-	-	-
Efflux ABC transporter ATP-binding protein	-	+	+	-	-
Efflux transporter	-	-	+	-	-
Efflux transporter ATP-binding and permease	-	-	+	-	-
Efflux transporter, RND family, MFP subunit	-	-	-	-	+
Ethanolamine transporter	-	-	+	-	-
Fe3+ ABC transporter substrate-binding protein	-	-	-	-	+
Ferrichrome ABC transporter ATP-binding protein	-	+	+	+	+
Ferrichrome ABC transporter ferrichrome-binding protein	-	+	-	-	-
Ferrichrome ABC transporter permease	-	+	+	+	+
Ferrichrome ABC transporter substrate-binding protein	+	-	+	-	-
Ferrichrome import ABC transporter ATP-binding protein fhuc	-	+	-	-	-
Ferrichrome import ABC transporter ferrichrome-binding protein	-	+	-	-	-
Ferrichrome import ABC transporter ferrichrome-binding protein fhud	-	+	-	-	-
Ferrichrome import ABC transporter permease	-	+	-	-	-
Ferrichrome import ABC transporter permease fhub	-	+	-	-	-
Ferrichrome import ABC transporter permease fhug	-	+	-	-	-
Ferrioxamine ABC transporter substrate-binding protein	-	-	+	-	-
Ferrous iron transporter B	-	-	-	-	+
Formate transporter	-	-	-	+	-
Formate/nitrite transporter	+	+	+	-	-
Formate/nitrite transporter family protein	-	-	-	-	+

Table S6 continued

**Fructoselysine transporter**	+	-	-	-	-
Glucarate transporter	+	-	+	-	-
Gluconate:H+ symporter (gntp) family transporter	-	+		-	+
Glucose/mannose transporter	-	-		+	-
Glutamine ABC transporter ATP-binding protein	-	-	+	-	-
Glutamine ABC transporter ATP-binding protein glnq	-	+	-	-	-
Glutamine ABC transporter glutamine-binding lipoprotein	-	-	+	-	-
Glutamine ABC transporter glutamine-binding protein glnh	-	+	-	-	-
Glutamine ABC transporter permease	-	-	+	-	-
Glutamine ABC transporter permease glnm	-	+	-	-	-
Glutamine ABC transporter permease glnp	-	+	-	-	-
**Glutamine ABC transporter substrate-binding protein**	+	-	-	-	-
Glutamine transporter	-	+	-	-	-
Glycerol-3-phosphate ABC transporter ATP-binding protein	-	+	-	-	-
Glycerol-3-phosphate ABC transporter glycerol-3-phosphate-binding protein	-	+	-	-	-
Glycerol-3-phosphate ABC transporter permease	-	+	-	-	-
**Glycerol-3-phosphate ABC transporter substrate-binding protein**	+	-	-	-	-
Glycerol-3-phosphate transporter	-	-	-	+	+
Glycine betaine ABC transporter permease	-	-	+	-	-
Glycine betaine ABC transporter substrate-binding protein	-	-	+	-	-
Glycine betaine ABC transporter, ATP-binding protein	-	-	-	-	+
Glycine betaine transporter	-	-	+	+	+
Glycine betaine/carnitine/choline/choline sulfate ABC transporter ATP-binding protein	-	-	+	-	-
Glycine betaine/carnitine/choline/choline sulfate ABC transporter osmoprotectant-binding lipoprotein	-	-	+	-	-
Glycine betaine/carnitine/choline/choline sulfate ABC transporter permease	-	-	+	-	-
**Glycine/betaine ABC transporter**	+	-	-	-	-
**Glycine/betaine ABC transporter atpase**	+	-	-	-	-
Glycine/betaine ABC transporter ATP-binding protein	+	-	+	-	-
Glycine/betaine ABC transporter ATP-binding protein opuaa	-	+	-	-	-
Glycine/betaine ABC transporter glycine/betaine- binding protein opuac	-	+	-	-	-
**Glycine/betaine ABC transporter permease**	+	-	-	-	-
Glycine/betaine ABC transporter permease opuab	-	+	-	-	-
Glycine/betaine/carnitine/choline ABC transporter ATP-binding protein Opu	-	+	-	-	-
Glycine/betaine/carnitine/choline ABC transporter permease/substrate-binding protein Opu	-	+	-	-	-
H+/amino acid transporter	-	-	+	-	-
Heme ABC transporter ATP-binding protein	+	-	+	-	-
Heme ABC transporter, heme-binding protein isde	-	-	-	-	+
**Heme transporter ccmc**	+	-	-	-	-
Hexuronate transporter	-	+	-	-	-
High affinity arginine ABC transporter ATP-binding protein	-	+	-	-	-
High affinity arginine ABC transporter permease	-	+	-	-	-

Table S6 continued

High affinity Zn(II) ABC transporter permease	-	+	-		-
His/Glu/Gln/Arg/opine family amino ABC transporter, permease, 3-TM region	-	-	-	-	+
**Histidine transporter**	+		-	-	-
Hypoxanthine efflux transporter	-	+	-	-	-
Inorganic phosphate transporter	+	-	-	+	-
**Ion transporter**	+	-	-	-	-
Iron (Fe) ABC superfamily ATP binding cassette transporter, membrane protein	-	-	-	-	+
**Iron ABC transporter**	+	-	-	-	-
**Iron ABC transporter atpase**	+	-	-	-	-
**Iron ABC transporter ATP-binding protein**	+	-	-	-	-
Iron ABC transporter permease	+	-	-	+	+
**Iron ABC transporter substrate-binding protein**	+	-	-	-	-
Iron compound ABC transporter, ATP-binding protein	-	-	-	-	-
Iron compound ABC transporter, iron compound-binding protein	-	-	-	-	-
Iron compound ABC transporter, permease protein	-	-	-	-	+
Iron(III) dicitrate transporter binding lipoprotein	-	-	-	-	+
Iron(III)-citrate import ABC transporter iron(III)-citrate-binding protein	+	-	+	-	-
Iron(III)-citrate import ABC transporter permease	-	-	+	-	-
**Iron(III)-siderophore transporter ATP binding protein**	+	-	-	-	-
Iron-dicitrate ABC transporter ATP-binding protein	-	-	+	-	-
Iron-dicitrate ABC transporter permease	-	-	+	-	-
**Iron-enterobactin transporter ATP-binding protein**	+	-		-	-
Iron-siderophore ABC transporter ATP-binding protein	-	-	+	-	-
Iron-siderophore ABC transporter permease	-	-	+	-	-
K+ efflux transporter	-	-	+	-	-
**Ktr system potassium transporter B**	+	-	-	-	-
**Lactose ABC transporter permease**	+	-	-	-	-
L-arabinose ABC transporter L-arabinose-binding protein aran	-	+	-	-	-
L-arabinose ABC transporter permease arap	-	+	-	-	-
L-arabinose ABC transporter permease araq	-	+	-	-	-
L-cystine import ABC transporter ATP-binding protein tcyc	-	+	-	-	-
L-cystine import ABC transporter L-cystine-binding protein tcya	-	+	-	-	-
L-cystine import ABC transporter permease tcyb	-	+	-	-	-
**L-cystine transporter tcyp**	+	-	-	-	-
Lipid ABC transporter ATP-binding protein	-	-	+	-	-
Low-affinity branched-chain amino acid transporter	-	-	+	-	-
Low-affinity inorganic phosphate transporter	-	-	+	-	-
Lyse family transporter	-	+	-	-	-
**Macrolide ABC transporter permease**	+	-	-	-	-
**Macrolide transporter**	+	-	-	-	-
Magnesium transporter	+	+	+	-	-
Magnesium/cobalt transporter cora	-	-	+	+	+

Table S6 continued

Major facilitator family transporter	+	+	-	+	+
Malonate transporter subunit madl	-	+	-	-	-
Malonate transporter subunit madm	-	+	-	-	-
Maltodextrin ABC transporter permease	-	-	+	-	-
Maltodextrin import ABC transporter ATP-binding protein msmx	-	+	-	-	-
Maltosaccharide ABC transporter, maltosaccharide-binding protein	-	-	-	-	+
Maltose and maltodextrin ABC transporter ATP-binding/permease malq	-	+	-	-	-
Maltose and maltodextrin ABC transporter subunit ATP-binding protein	-	-	+	-	-
Maltose and multiple sugar ABC transporter permease amyc	-	+	-	-	-
Maltose and multiple sugars ABC transporter permease	-	-	+	-	-
Manganese ABC transporter ATP-binding protein	+	-	+	-	-
Manganese ABC transporter manganese binding lipoprotein	-	-	+	-	-
Manganese ABC transporter permease	+	-	+	-	-
**Manganese transporter**	**+**	**-**	**-**	-	-
Membrane protein, possible ABC transporter, permease component	-	-	-	-	+
Metabolite transporter	-	-	+	-	-
Metabolite-efflux transporter	-	-	+	-	-
Metal ABC transporter ATP-binding protein	-	+	-	-	-
Metal ABC transporter metal-binding protein	-	+	-	-	-
Metal ABC transporter permease	+	+	-	-	-
**Metal transporter**	**+**	**-**	**-**	-	-
Methionine ABC transporter ATP-binding protein	+	-	+	-	-
Methionine ABC transporter permease	+	-	+	-	-
Methionine ABC transporter substrate-binding protein	+	-	-	-	-
Methionine ABC transporter, substrate binding lipoprotein		-	+	-	-
Methionine import ABC transporter ATP-binding protein metn	-	+	-	-	-
Methionine import ABC transporter methionine-binding protein Met	-	+	-	-	-
Methionine import ABC transporter methionine-binding protein metq	-	+	-	-	-
Methionine import ABC transporter permease Met	-	+	-	-	-
Methionine import ABC transporter permease metp	-	+	-	-	-
**Methyl-galactoside ABC transporter substrate-binding protein**	**+**	**-**	**-**	-	-
Methylthioribose ABC transporter ATP-binding protein	-	-	-	+	-
Methylthioribose ABC transporter permease	-	-	-	+	-
MFS transporter	+	-	+	-	+
Mg2+ transporter mgte	-	-	-	-	+
Mmpl family transporter	-	+	-	-	-
Molybdate ABC transporter molybdate-binding protein moda	-	+	-	-	-
Molybdate ABC transporter permease modb	-	+	-	-	-
Molybdenum ABC transporter permease	+	-	-	+	+
Molybdenum ABC transporter substrate-binding protein	+	-	-	-	-
Molybdenum ABC transporter, periplasmic molybdate-binding protein	-	-	-	-	+
Monovalent cation:proton antiporter-2 (CPA2) family transporter	-	-	-	-	+

Table S6 continued

Multidrug ABC transporter	-	-	-	+	+
**Multidrug ABC transporter atpase**	+	-	-	-	-
**Multidrug ABC transporter ATP-binding protein**	+	-	-	-	-
**Multidrug ABC transporter permease**	+	-	-	-	-
Multidrug ABC transporter permease/ATP-binding protein	-	-	-	+	-
Multidrug ABC transporter, ATP-binding protein	-	-	-	-	+
Multidrug ABC transporter, permease component	-	-	-	-	+
**Multidrug DMT transporter permease**	+	-	-	-	-
Multidrug export ABC transporter ATP-binding/permease	-	+	-	-	-
**Multidrug MFS transporter**	+	-	-	-	-
Multidrug resistance ABC transporter ATP-binding/permease	-	+	-	-	-
**Multidrug transporter**	+	-	-	-	-
**Multidrug transporter mate**	+	-	-	-	-
Multidrug-efflux transporter	-	-	+	-	+
Multidrug-efflux transporter 2 regulator	-	+	-	-	+
Multiple sugar ABC transporter ATP-binding protein	-	-	+	-	-
Multiple sugar ABC transporter multiple sugar-binding protein msme	-	+	-	-	-
Multiple sugar-binding transporter ATP-binding protein	-	-	+	-	-
Myo-inositol transporter	-	-	+	-	-
Myo-inositol transporter iolt	-	+	-	-	-
Na/Pi cotransporter	+	-	-	-	+
Na+ ABC efflux transporter ATP-binding protein	-	-	+	-	-
Na+ ABC efflux transporter permease	-	-	+	-	-
Na+ dependent nucleoside transporter domain protein	-	-	-	-	+
Na+(H+)/nucleoside cotransporter	-	-	+	-	-
Na+/anion cotransporter	-	-	+	-	-
Na+/metabolite cotransporter	-	-	+	-	-
Na+driven efflux transporter	-	-	+	-	-
Na+-efflux ABC transporter ATP-binding protein	-	-	+	-	-
NCS1 nucleoside transporter family	-	+	-	-	-
Negatively charged metabolite transporter	-	-	+	-	-
Ni2+-Co2+ transporter (nicot) family transition metal uptake transporter	-	-	-	-	+
Nickel import ABC transporter ATP-binding protein nikd	-	+	-	-	-
Nickel import ABC transporter ATP-binding protein nike	-	+	-	-	-
Nickel import ABC transporter nickel-binding protein nika	-	+	-	-	-
Nickel import ABC transporter permease subunit nikb	-	+	-	-	-
Nickel import ABC transporter permease subunit nikc	-	+	-	-	-
Nickel transporter	-	+	-	-	-
**Nicotinamide riboside transporter pnuc**	+	-	-	-	-
**Nitrate ABC transporter permease**	+	-	-	-	-
**Nitrate ABC transporter substrate-binding protein**	+	-	-	-	-
Nitrate transporter	-	-	+	+	+

Table S6 continued

Nucleoside transporter nupc	+	+	-	-	+
Oligopeptide ABC transporter ATP-binding protein	-	+	+	-	+
Oligopeptide ABC transporter ATP-binding protein appd	-	+	-	-	-
Oligopeptide ABC transporter ATP-binding protein appf	-	+	-	-	-
Oligopeptide ABC transporter ATP-binding protein oppf	-	+	-	-	-
Oligopeptide ABC transporter binding lipoprotein	-	-	+	-	-
Oligopeptide ABC transporter oligopeptide-binding protein	-	+	-	-	-
Oligopeptide ABC transporter oligopeptide-binding protein appa	-	+	-	-	-
Oligopeptide ABC transporter oligopeptide-binding protein oppa	-	+	-	-	-
Oligopeptide ABC transporter permease	-	+	+	-	-
Oligopeptide ABC transporter permease appb	-	+	-	-	-
Oligopeptide ABC transporter permease appc	-	+	-	-	-
Oligopeptide ABC transporter permease oppb	-	+	-	-	-
Oligopeptide ABC transporter permease oppc	-	+	-	-	-
Oligopeptide ABC transporter substrate-binding protein	-	+	-	-	-
Oligopeptide ABC transporter, oligopeptide-binding protein	-	-	-	-	+
Oligopeptide ABC transporter, permease	-	-	-	-	+
Oligopeptide ABC transporter, solute-binding protein	-	-	-	-	+
Oligopeptide transporter, periplasmic-binding protein	-	-	-	-	+
Oligopeptide/dipeptide ABC transporter, ATP-binding protein domain protein	-	-	-	-	+
Osmoprotectant transporter ousa	-	+	-	-	-
Osmoregulated proline transporter	-	+	-	-	-
**Peptide ABC transporter ATP-binding protein**	**+**	**-**	**-**	-	-
**Peptide ABC transporter permease**	**+**	**-**	**-**	-	-
**Peptide ABC transporter substrate-binding protein**	**+**	**-**	**-**	-	-
Phosphate ABC transporter ATP-binding protein	+	+	+	-	-
Phosphate ABC transporter permease	+	+	+	+	-
Phosphate ABC transporter, permease protein psta	-	-	-	-	+
Phosphate ABC transporter, permease pstc	-	-	-	-	+
Phosphate ABC transporter, phosphate-binding protein	-	-	-	-	+
Phosphate transporter	-	+	-	-	-
Phosphate transporter ATP-binding protein	-	-	-	+	-
Phosphate transporter family protein	-	-	-	-	+
Phosphate transporter phou	-	-	-	+	
Phosphate/phosphite/phosphonate ABC transporter, periplasmic binding protein	-	-	-	-	+
Phosphoglycerate transporter	-	-	-	+	-
Phosphoglycerate transporter family protein	-	-	-	-	+
**Phosphate ABC transporter substrate-binding protein**	**+**	**-**	**-**	-	-
**Pn transporter membrane channel protein**	**+**	**-**	**-**	-	-
Potassium transporter atpase	-	+	+	-	-
**Potassium transporter CPA**	**+**	**-**	**-**	-	-
**Potassium transporter ktra**	**+**	**-**	**-**	-	-

Table S6 continued

**Potassium transporter Trk**	+	-	-	-	-
Proline transporter	-	-	+	-	-
Proline/betaine transporter	-	-	-	+	+
Proton metabolite efflux transporter	-	-	+	-	-
**Proton-coupled thiamine transporter yuaj**	+	-	-	-	-
**PTS cellbiose transporter subunit IIB**	+	-	-	-	-
**PTS cellobiose transporter subunit IIA**	+	-	-	-	-
**PTS cellobiose transporter subunit IIB**	+	-	-	-	-
**PTS cellobiose transporter subunit IIC**	+	-	-	-	-
**PTS fructose transporter subunit IIA**	+	-	-	-	-
**PTS fructose transporter subunit IIC**	+	-	-	-	-
**PTS glucose transporter subunit IIA**	+	-	-	-	-
**PTS glucose transporter subunit IIBC**	+	-	-	-	-
**PTS mannitol transporter subunit IIABC**	+	-	-	-	-
**PTS sorbitol transporter subunit IIA**	+	-	-	-	-
**PTS sorbitol transporter subunit IIB**	+	-	-	-	-
**PTS sorbitol transporter subunit IIC**	+	-	-	-	-
**PTS sugar transporter**	+	-	-	-	-
**PTS sugar transporter subunit IID**	+	-	-	-	-
PTS system beta-glucoside-specific transporter subunit IIBCA	-	-	+	-	-
PTS system diacetylchitobiose-specific transporter subunit IIB	-	+	-	-	-
PTS system fructose-specific transporter subunit IIA	-	+	+	-	-
PTS system fructose-specific transporter subunit IIABC	-	+	+	-	-
PTS system fructose-specific transporter subunit IIB	-	+	+	-	-
PTS system fructose-specific transporter subunit IIC	-	+	+	-	-
PTS system fructose-specific transporter subunit IID	-	+	+	-	-
PTS system glucosamine-specific transporter subunit IICBA	-	-	+	-	-
PTS system glucose subfamily transporter	-	+	-	-	-
PTS system glucose-specific transporter subunit IIBC	-	+	-	-	-
PTS system glucose-specific transporter subunit IICBA	-	-	+	-	-
PTS system lactose/cellobiose specific transporter subunit IIB	-	+	-	-	-
PTS system lichenan-specific transporter subunit IIA	-	+	+	-	-
PTS system lichenan-specific transporter subunit IIB	-	+	+	-	-
PTS system lichenan-specific transporter subunit IIC	-	+	+	-	-
PTS system mannitol-specific transporter subunit IIA	-	-	+	-	-
PTS system mannitol-specific transporter subunit IIC	-	+	-	-	-
PTS system mannitol-specific transporter subunit IICB	-	-	+	-	-
PTS system mannose-specific transporter subunit IIBCA	-	-	+	-	-
PTS system N-acetylglucosamine-specific transporter subunit EIICBA	-	+	-	-	-
PTS system N-acetylglucosamine-specific transporter subunit IICB	-	-	+	-	-
PTS system oligo-alpha-mannoside transporter subunit IIA	-	-	+	-	-
PTS system oligo-alpha-mannoside transporter subunit IIB	-	-	+	-	-

Table S6 continued

PTS system oligo-alpha-mannoside transporter subunit IIC	-	-	+	-	-
PTS system sucrose-specific transporter subunit IIABC	-	+	-	-	-
PTS system sucrose-specific transporter subunit IIBC	-	+	+	-	-
PTS system transporter I	-	+	-	-	-
PTS system transporter subunit IIA	-	-	+	-	-
PTS system transporter subunit IIBC	-	+	-	-	-
PTS system transporter subunit IIC	-	-	+	-	-
PTS system trehalose-specific transporter subunit IIBC	-	+	+	-	-
Purine nucleoside transporter	-	-	+	-	-
Putative ABC transporter ATP-binding protein	-	-	+	-	-
Putative ABC transporter, substrate-binding protein	-	-	-	-	+
Putative bacitracin ABC transporter, permease protein	-	-	-	-	+
Putative glycerol-3-phosphate ABC transporter, ATP-binding protein	-	-	-	-	+
Putative iron compound ABC transporter, permease protein	-	-	-	-	+
Putative MFS-type transporter	-	-	-	-	+
Putative phosphate ABC transporter, permease protein	-	-	-	-	+
Putative transporter	-	-	-	-	+
Pyrimidine nucleoside transporter	-	-	-	-	+
Pyrimidine-nucleoside Na+(H+) cotransporter	-	-	+	-	-
**Quaternary ammonium transporter**	+	-	-	-	-
Quinolone resistance protein, major facilitator family transporter	-	-	-	-	+
Rbsb (Ribose ABC transporter)	-	-	-	-	+
**Riboflavin transporter fmnp**	+	-	-	-	-
Ribose ABC transporter	-	-	-	-	+
Ribose ABC transporter ATP-binding protein	-	-	+	+	-
Ribose ABC transporter ATP-binding protein rbsa	-	+	-	-	-
Ribose ABC transporter permease	-	-	+	+	-
Ribose ABC transporter permease rbsc	-	+	-	-	-
Ribose ABC transporter protein	-	-	-	-	+
Ribose ABC transporter ribose-binding lipoprotein	-	-	+	-	-
Ribose ABC transporter ribose-binding protein rbsb	-	+	-	-	-
RND family efflux transporter MFP subunit	-	+	-	-	-
Secondary transporter of divalent metal ions/citrate complexes	-	-	+	-	-
Serine transporter	-	-	-	+	+
**Short-chain fatty acid transporter**	+	-	-	-	-
**Siderophore ABC transporter permease**	+	-	-	-	-
Small conductance mechanosensitive ion channel (mscs) family transporter protein	-	+	-	-	-
Small multidrug efflux transporter	-	-	+	-	-
Small multidrug resistance efflux transporter	-	-	+	-	-
**Sodium ABC transporter ATP-binding protein**	+	-	-	-	-
Sodium ABC transporter permease	+	-	+	-	-
Sodium dependent transporter	-	-	+	-	-

Table S6 continued

Sodium efflux ABC transporter ATP-binding protein nata	-	+	-	-	-
Sodium efflux ABC transporter permease natb	-	+	-	-	-
**Sodium transporter**	**+**	**-**	**-**	-	-
Sodium/proton-dependent alanine transporter	-	-	+	-	-
Sodium-dependent leucine transporter	-	-	-	+	-
Sodium-dependent phosphate transporter	-	-	-	+	-
Sodium-dependent serine transporter	-	+	+	-	+
Sodium-dependent tryptophan transporter	-	-	-	+	+
Solute:sodium symporter (SSS) family transporter	-	+	-	-	-
**Spermidine/purescine ABC transporter permease**	**+**	**-**	**-**	-	-
Spermidine/putrescine ABC transporter ATP-binding protein	+	-	+	+	-
Spermidine/putrescine ABC transporter permease	+	-	-	+	+
**Spermidine/putrescine ABC transporter substrate-binding protein**	**+**	**-**	**-**	-	-
Spermidine/putrescine import ABC transporter ATP-binding protein pota	-	+	-	-	-
Spermidine/putrescine import ABC transporter permease potb	-	+	-	-	-
Spermidine/putrescine import ABC transporter permease potc	-	+	-	-	-
Spermidine/putrescine import ABC transporter spermidine/putrescine-binding protein potd	-	+	-	-	-
Stress adaptation transporter	-	-	+	-	-
Sublancin 168 lantibiotic transporter	-	-	+	-	-
Subtilosin production transporter	-	-	+	-	-
Sugar (Glycoside-Pentoside-Hexuronide) transporter	-	+	-	-	-
**Sugar ABC transporter atpase**	**+**	**-**	**-**	-	-
Sugar ABC transporter ATP-binding protein	+	+	-	-	-
**Sugar ABC transporter permease**	**+**	**-**	**-**	-	-
**Sugar ABC transporter substrate-binding protein**	**+**	**-**	**-**	-	-
Sugar ABC transporter, permease protein	-	-	-	-	+
Sugar efflux transporter (Major Facilitator family)	-	+	-	-	-
**Sugar MFS transporter**	**+**	**-**	**-**	-	-
Sugar transporter	+	-	+	-	-
Sulfate ABC transporter ATP-binding protein	-	+	-	-	-
Sulfate ABC transporter permease cyst	-	+	-	-	-
Sulfate ABC transporter permease cysw	-	+	-	-	-
Sulfate ABC transporter sulfate-binding protein	-	+	-	-	-
Sulfate ABC transporter, inner membrane subunit cysw	-	-	-	-	+
Sulfate ABC transporter, permease protein cyst	-	-	-	-	+
Sulfate transporter	+	+	-	+	+
Sulfate transporter family protein	-	+	-	-	-
**Sulfonate ABC transporter permease**	**+**	**-**	**-**	-	-
Sulfur containing amino acid ABC transporter binding lipoprotein	-	-	+	-	-
Sulfur-containing amino acid ABC transporter ATP-binding protein tcyn	-	+	-	-	-
Sulfur-containing amino acid ABC transporter permease	-	-	+	-	-
Sulfur-containing amino acid ABC transporter permease tcyl	-	+	-	-	-

Table S6 continued

Sulfur-containing amino acid ABC transporter permease tcym	-	+	-	-	-
Sulfur-containing amino acid ABC transporter sulfur-containing amino acid-binding protein tcyj	-	+	-	-	-
Sulfur-containing amino acid ABC transporter sulfur-containing amino acid-binding protein tcyk	-	+	-	-	-
Sulfur-containing amino-acid ABC transporter ATP-binding protein	-	-	+	-	-
Surfactin self-resistance transporter	-	-	+	-	-
Taurine ABC transporter ATP-binding protein	-	+	-	-	-
Taurine ABC transporter permease	-	+	-	-	-
Taurine ABC transporter taurine-binding protein	-	+	-	-	-
Thiamine ABC transporter ATP-binding protein	-	-	+	-	-
Thiamine transporter	-	-	-	+	-
Thiamine transporter permease	-	-	+	-	-
Thiamine transporter protein (Thia_yuaj) family protein, partial	-	+	-	-	-
Threonine transporter	-	-	-	+	-
Toxic compound efflux transporter	-	-	+	-	-
Transporter	+	-	+	+	+
Transporter family protein	-	-	-	+	-
Transporter membrane protein	-	+	-	-	-
Transporter of divalent metal ions/citrate complexes	-	-	+	-	-
Transporter yvqf	-	-	-	-	+
Transporter, drug/metabolite exporter	-	-	-	-	+
Transporter, eama	-	-	-	-	+
Transporter, lyse	-	-	-	-	+
Transporter, MFS superfamily	-	-	-	-	+
Transporter, Sodium/bile acid symporter	-	-	-	-	+
TRAP transporter, dctm-like membrane protein	-	+	-	-	-
Tripartite tricarboxylate transporter tcta	+	-	-	-	-
Tryptophan transporter	+	-	+	+	-
Uracil transporter	+	-	-	-	-
Ycki (ABC transporter ycki)	-	-	-	-	+
**Zinc ABC transporter ATP-binding protein**	**+**	**-**	**-**	-	-
Zinc transporter	-	+	-	+	-
Zinc uptake transporter	-	-	-	+	+
Zn transporter	-	-	+	-	-
Zn(II) transporter ATP-binding protein	-	-	+	-	-

Bold, unique to *Bacillus* sp. NRRL B-14911; +, present; -, absent.

Table S7: Comparison of the nucleotide sequences and amino acid sequences corresponding to the epitope BAC 25-40 from *Bacillus* sp. NRRL B-14911and *B. infantis* JCM 13438T.

Name	Sequence
	Nucleotide†
Bacillus sp. NRRL B-14911	GAGGGCTTCACCCGCCTCAGCTTCACCGCCGAAGAAAAAGCTGCCCAT
Bacillus infantis JCM 13438T	GAGGGCTTCACCCGCCTCAGCTTCACCGCCGAGGAAAAAGCTGCCCAT
	Amino acid
Bacillus sp. NRRL B-14911	EGFTRLSFTAEEKAAH
Bacillus infantis JCM 13438T	EGFTRLSFTAEEKAAH

†underlined codon shows the silent mutation

Table S8: Comparative analysis of the presence or absence of enzymes and biochemical pathways of *Bacillus* sp. NRRL B-14911 in relation to other *Bacillus* species.

Parameter	*Bacillus* sp. NRRL B-14911[†]	*B. subtilis* strain 168	*B. megaterium* DSM 319	*B. thuringiensis* serovar kurstaki strain HD73	*B. cereus* ATCC 14579
Enzymes					
D-alanine transaminase	+	+	+	+	+
Glutamate racemase (*murI*)	+	+	+	+	+
Glutamine synthetase	+	+	+	+	+
Biochemical pathways					
Atrazine degradation	0	-	+	-	-
Biosynthesis of siderophore group nonribosomal peptides	0	+	-	+	+
Bisphenol degradation	0	+	-	-	+
Carotenoid biosynthesis	7	+	+	-	-
Dioxin degradation	0	-	+	+	+
Ethylbenzene degradation	0	-	+	-	+
Geraniol degradation	0	+	+	-	+
Limonene and pinene degradation	0	+	+	-	+
**Lipopolysaccharide biosynthesis**	**1**	-	-	-	-
Nitrotoluene degradation	0	-	+	+	-
Non-homologous end-joining	2	+	-	-	-
Nonribosomal peptide structures	1	+	-	+	+
Other glycan degradation	1	+	+	-	-
Penicillin and cephalosporin biosynthesis	2	+	-	-	+
Phosphonate and phosphinate metabolism	0	-	+	+	+
Polycyclic aromatic hydrocarbon degradation	0	-	+	-	+
Polyketide sugar unit biosynthesis	6	+	-	+	+
Sphingolipid metabolism	2	+	+	-	-
**Steroid biosynthesis**	**1**	-	-	-	-
Styrene degradation	3	-	-	+	+
α-Linolenic acid metabolism	0	+	+	-	+
β-Lactam resistance	0	-	+	+	+
ABC transporters	129	+	+	+	+
Alanine, aspartate and glutamate metabolism	27	+	+	+	+
Amino sugar and nucleotide sugar metabolism	43	+	+	+	+
Aminoacyl-tRNA biosynthesis	118	+	+	+	+
Aminobenzoate degradation	5	+	+	+	+
Arachidonic acid metabolism	5	+	+	+	+
Arginine and proline metabolism	46	+	+	+	+
Ascorbate and aldarate metabolism	8	+	+	+	+
Bacterial chemotaxis	30	+	+	+	+
Bacterial secretion system	14	+	+	+	+
Base excision repair	15	+	+	+	+
Benzoate degradation	8	+	+	+	+

Table S8 continued

beta-Alanine metabolism	13	+	+	+	+
Biosynthesis of unsaturated fatty acids	11	+	+	+	+
Biotin metabolism	13	+	+	+	+
Butanoate metabolism	19	+	+	+	+
C5-Branched dibasic acid metabolism	6	+	+	+	+
Chloroalkane and chloroalkene degradation	9	+	+	+	+
Citrate cycle (TCA cycle)	27	+	+	+	+
Cyanoamino acid metabolism	7	+	+	+	+
Cysteine and methionine metabolism	41	+	+	+	+
D-Alanine metabolism	3	+	+	+	+
D-Arginine and D-ornithine metabolism	1	+	+	+	+
D-Glutamine and D-glutamate metabolism	4	+	+	+	+
DNA replication	18	+	+	+	+
Fatty acid biosynthesis	23	+	+	+	+
Flagellar assembly	34	+	+	+	+
Folate biosynthesis	17	+	+	+	+
Fructose and mannose metabolism	19	+	+	+	+
Galactose metabolism	19	+	+	+	+
Glutathione metabolism	11	+	+	+	+
Glycerolipid metabolism	15	+	+	+	+
Glycerophospholipid metabolism	23	+	+	+	+
Glycine, serine and threonine metabolism	34	+	+	+	+
Glycolysis / Gluconeogenesis	48	+	+	+	+
Glyoxylate and dicarboxylate metabolism	32	+	+	+	+
Histidine metabolism	22	+	+	+	+
Homologous recombination	21	+	+	+	+
Inositol phosphate metabolism	4	+	+	+	+
Lipoic acid metabolism	3	+	+	+	+
Lysine biosynthesis	16	+	+	+	+
Lysine degradation	14	+	+	+	+
Methane metabolism	29	+	+	+	+
Mismatch repair	22	+	+	+	+
Naphthalene degradation	3	+	+	+	+
Nicotinate and nicotinamide metabolism	12	+	+	+	+
Nitrogen metabolism	7	+	+	+	+
Novobiocin biosynthesis	3	+	+	+	+
Nucleotide excision repair	11	+	+	+	+
One carbon pool by folate	11	+	+	+	+
Oxidative phosphorylation	45	+	+	+	+
Pantothenate and CoA biosynthesis	19	+	+	+	+
Pentose and glucuronate interconversions	21	+	+	+	+
Pentose phosphate pathway	31	+	+	+	+
Peptidoglycan biosynthesis	23	+	+	+	+
Phenylalanine metabolism	20	+	+	+	+

Table S8 continued

Phenylalanine, tyrosine and tryptophan biosynthesis	20	+	+	+	+
Phosphotransferase system (PTS)	16	+	+	+	+
Porphyrin and chlorophyll metabolism	21	+	+	+	+
Propanoate metabolism	31	+	+	+	+
Protein export	19	+	+	+	+
Purine metabolism	62	+	+	+	+
Pyrimidine metabolism	59	+	+	+	+
Pyruvate metabolism	42	+	+	+	+
Riboflavin metabolism	6	+	+	+	+
Ribosome	82	+	+	+	+
RNA degradation	16	+	+	+	+
RNA polymerase	5	+	+	+	+
Secondary bile acid biosynthesis	1	+	+	+	+
Selenocompound metabolism	17	+	+	+	+
Sesquiterpenoid and triterpenoid biosynthesis	2	+	+	+	+
Starch and sucrose metabolism	34	+	+	+	+
Streptomycin biosynthesis	10	+	+	+	+
Sulfur metabolism	15	+	+	+	+
Sulfur relay system	11	+	+	+	+
Synthesis and degradation of ketone bodies	7	+	+	+	+
Taurine and hypotaurine metabolism	7	+	+	+	+
Terpenoid backbone biosynthesis	15	+	+	+	+
Thiamine metabolism	13	+	+	+	+
Toluene degradation	4	+	+	+	+
Tryptophan metabolism	20	+	+	+	+
Two-component system	105	+	+	+	+
Tyrosine metabolism	10	+	+	+	+
Ubiquinone and other terpenoid-quinone biosynthesis	9	+	+	+	+
Valine, leucine and isoleucine biosynthesis	12	+	+	+	+
Valine, leucine and isoleucine degradation	30	+	+	+	+
Vitamin B6 metabolism	6	+	+	+	+
Xylene degradation	2	+	+	+	+

Bold, unique to *Bacillus* sp. NRRL B-14911; +, present; -, absent; †, numbers indicate the number of genes present in *Bacillus* sp. NRRL B-14911.

Table S9: Comparison of insertion sequence elements between *Bacillus* sp. NRRL B-14911 and other *Bacillus* species.

IS family	*Bacillus* sp. NRRL B-14911	*B. subtilis* strain 168	*B. megaterium* DSM 319	*B. thuringiensis* serovar kurstaki strain HD73	*B. cereus* ATCC 14579
**IS1**	**1**	**0**	**0**	**0**	**0**
IS1;ISMhu11	1	0	0	1	0
IS110	2	1	3	5	3
IS110;IS1111	1	0	1	2	2
IS1182	4	6	9	4	5
IS1380	0	3	1	1	1
**IS1380;IS942**	**1**	**0**	**0**	**0**	**0**
IS1595	0	0	0	0	1
IS1595;ISNwi1	2	0	0	3	1
IS1634	0	3	2	3	3
IS200/IS605	0	0	1	3	5
IS200/IS605;IS1341	0	0	0	4	2
IS200/IS605;IS200	0	0	1	0	0
IS21	1	2	4	3	2
IS256	2	3	3	0	0
IS3	3	5	2	8	5
IS3;IS150	3	4	3	34	25
IS3;IS3	0	0	1	1	0
IS3;IS407	2	0	2	0	0
IS3;IS51	1	1	0	1	0
IS30	0	1	2	0	0
IS4;IS231	1	1	20	35	34
IS4;IS50	0	1	1	0	0
IS4;ISH8	1	0	0	0	0
IS4;ISPepr1	1	1	0	0	0
IS481	3	0	3	0	0
IS5	0	2	0	2	2
IS5;IS427	0	0	0	1	1
**IS5;IS903**	**1**	**0**	**0**	**0**	**0**
IS6	0	0	3	2	7
IS607	0	0	5	6	3
IS630	0	0	1	2	2
IS66	1	1	1	0	3
IS66;ISBst12	1	0	1	1	0
IS91	1	0	0	0	1
IS982	0	0	0	2	1
ISAs1	0	0	0	2	0
ISAzo13	0	1	0	0	1
ISH3	0	1	1	1	1
ISL3	1	0	0	1	1
ISLre2	1	0	1	3	3
ISLre2;ISAzba1	0	1	0	0	0
ISLre2;ISKra4	0	0	0	0	2
ISNCY	0	0	0	1	0
ISNCY;IS1202	0	1	0	0	0
ISNCY;ISLbi1	0	0	0	1	1
**Tn3**	**1**	**0**	**0**	**0**	**0**

Bold, unique to *Bacillus* sp. NRRL B-14911
